# Selective sweep with significant positive selection serves as the driving force for the differentiation of *japonica* and *indica* rice cultivars

**DOI:** 10.1186/s12864-017-3702-x

**Published:** 2017-04-19

**Authors:** Yang Yuan, Qijun Zhang, Shuiyun Zeng, Longjiang Gu, Weina Si, Xiaohui Zhang, Dacheng Tian, Sihai Yang, Long Wang

**Affiliations:** 1 The Applied Plant Genomics Laboratory, College of Agricultural Sciences, Nanjing Agricultural University, Nanjing, 210095 China; 2 State Key Laboratory of Pharmaceutical Biotechnology, School of Life Sciences, Nanjing University, Nanjing, 210023 China; 3 Jiangsu Academy of Agricultural Sciences, Nanjing, 210014 China

**Keywords:** *japonica*, *indica*, Wild rice, Domestication, Resequencing, Functional genes

## Abstract

**Background:**

Asian cultivated rice (*Oryza sativa* L.), including *japonica* and *indica*, is unarguable the most important crop in Asia as well as worldwide. However, a decisive conclusion of its origination and domestication processes are still lacking. Nowadays, the ever-increasing high-throughput sequencing data of numerous rice samples have provided us new opportunities to get close to the answer of these questions.

**Results:**

By compiling 296 whole-genome sequenced rice cultivars and 39 diverse wild rice, two types of domesticated regions (DR-I and DR-II) with strong selective sweep signals between different groups were detected. DR-I regions included 28 blocks which significantly differentiated between *japonica* and *indica* subspecies, while DR-II regions were consisted of another 28 blocks which significantly differentiated between wild and cultivated rice, each covered 890 kb and 640 kb, respectively. In-depth analysis suggested that both DR-Is and DR-IIs could have originated from Indo-China Peninsula to southern China, and DR-IIs might be introgressed from *indica* to *japonica*. Functional bias with significant positive selection has also been detected in the genes of DR-I, suggesting important role of the selective sweep in differentiation of *japonica* and *indica*.

**Conclusions:**

This research promoted a new possible model of the origin of the cultivated rice that DR-Is in *japonica* and *indica* maybe independently originated from the divergent wild rice in the Indo-China Peninsula to southern China, and then followed by frequent introgression. Genes with significant positive selection and biased functions were also detected which could play important roles in rice domestication and differentiation processes.

**Electronic supplementary material:**

The online version of this article (doi:10.1186/s12864-017-3702-x) contains supplementary material, which is available to authorized users.

## Background

As one of the most important cereal grains, rice has been used as a major food source for more than half of the world’s population [1]. Asian cultivated rice is divided into two subspecies, *Oryza sativa* ssp. *japonica* and *Oryza sativa* ssp. *indica*. The *japonica* varieties are adapted to more temperate climates, whereas most *indica* varieties are generally adapted to tropical lowland cultivation [2]. The distinction between the two rice subspecies has been recognized in China since at least the Han dynasty [3].

However, the origin(s) of the two subspecies has long been under scrutiny and debate, particularly on whether the two subspecies were derived from a single or multiple domestications [4]. Numerous studies have suggested that the two subspecies of *indica* and *japonica* were probably independently domesticated from different isolates of the wild rice, *Oryza rufipogon* [5, 6], which was then followed by further differentiation [7]. Londo et al. believed that *indica* might have originated from a region south of the Himalaya mountain range, whereas *japonica* originated from wild rice in southern China [8]. On the other hand, Molina et al. reported that Asian rice might have been domesticated from a single origin [9]. A more recent study supports the viewpoint of a single origin, indicating that Asian rice cultivars might have been domesticated from southern China [10]. The comprehensive and deeper survey of the genetic basis of domestication may contribute to improved domestication strategies of organisms and provide novel approaches in deciphering the process of domestication [11–13]. To date, the debate on the origin of Asian rice cultivars remains elusive, thus requiring additional evidence to resolve this issue.

Ongoing works have already characterized a large number of genes, such as *Sub1A* [14], *S-5* [15], and *NRT1.1B* [16], which were demonstrated to be involved in differentiation between the two subspecies. Similarly, some other genes are related to domestication from wild to cultivated rice, including *Bh4* [17], *PROG1* [18] and *sh4* [19]. It is essential to utilize these genes in distinguishing the two subspecies or wild rice from rice cultivars. However, information on the mechanism underlying the differences in morphological, physiological, and biochemical features, as well as some other aspects between the two subspecies or wild rice and cultivated rice is limited. Moreover, the agronomic traits may be controlled by multiple genes, which were difficult to be identified [20]. Therefore, additional studies that aim to identify genes that are involved in the domestication of rice at the whole-genome scale using new methods such as whole genome sequencing technology are warranted [10, 20].

Recently, a batch of 3,000 rice accessions have been fully sequenced with high coverage [21], which provided unprecedented opportunities to explore the differentiation between *indica* and *japonica* via genomic approaches. By characterizing the two types of domesticated regions according to selection signatures and subsequent phylogenetic analysis, we delimited the potential zone for origins of the two subspecies, that is, the two subspecies may have a common origin in the Indo-China Peninsula. In addition, significant positive selection and particular genes that possibly control important morphological traits between cultivars and wild rice, as well as differentiation between *indica* and *japonica* were identified in these two types of domesticated regions, which suggests that selective sweep with significant positive selection may serve as the driving force for the differentiation of the two rice subspecies.

## Results

### Selection of the rice accessions

Although the 3,000 rice genomes dataset [21] provides an unprecedented resource for detecting the selective sweep regions in rice cultivars, most of which have low-coverage or inadequate sequencing depths that are difficult to employ in the high-resolution detection analysis due to the frequent coverage gaps. Therefore, 330 cultivars with ≥15× sequencing depths were downloaded from the 3,000 samples (Additional file 1: Table S1). To detect the regions with significant differentiation between *japonica* and *indica*, only those cultivars with significant differentiation that was detected by diversity calculation and PCA were retained (Additional file 1: Figure S1 and Table S2 see Methods). Finally, 296 out of the 330 rice cultivars, including 154 *indica* and 142 *japonica* cultivars, from 45 different countries (or regions), were used for further analysis, which have 23.8× average sequencing depth (ranging from 15.0× to 51.1×; Additional file 1: Table S1). The other 34 cultivars were removed due to the potential genetic admixtures of these two subspecies (Additional file 1: Table S1). And the retained cultivars exhibited scattered geographic distribution. About 1/3 of these cultivars were collected from China and India, both of which are geographically larger. Another 1/3 were from countries in Southeast Asia, including Thailand, Burma, Cambodia and so on. The last 1/3 were from other countries, such as Japan, South Korea and so on.

In addition, the sequences of 39 diverse wild rice *O. rufipogon* or *O. nivara* (also referred to annual of *O. rufipogon*) accessions, which were believed to be the immediate progenitor of the Asian cultivated rice of *O. sativa*, were collected from previous reports [10] (Additional file 1: Table S3). These sequences have 11× sequencing depth on average (ranging from 4.0× to 68.6×). On the other hand, the whole-genome sequences of 20 African rice *O. glaberrima* accessions, which are closely related to *O. sativa* and *O. rufipogon*, were downloaded at a 42.0× average sequencing depth (ranging from 5.9× to 120.3×; Additional file 1: Table S3) as outgroups for further analysis.

### Nucleotide diversity/divergence within or between these two subspecies

After trimming and removing low-quality bases, the clean reads of 296 Asian cultivated rice, 39 wild rice, and 20 Africa cultivated rice accessions were mapped to the *Nipponbare* reference genome. Then, joint SNP calling and genotyping of the sequenced samples were performed (see Methods). After stringent filtering, a total of 23,147,437 SNPs across all the 355 various rice samples were called and used to estimate diversity among samples or divergence among groups.

Based on the SNP data, π was estimated at 0.00196 within *japonica* and 0.00265 within *indica* groups (Additional file 1: Figure S2), which were higher than that within *japonica* (0.0006) and *indica* (0.0016) that were estimated in 517 Chinese *indica* and *japonica* landraces [22]. These discrepancies may be due to (i) higher genetic diversity in the worldwide cultivars than that only in Chinese landraces; (ii) an underestimation of the genetic diversity in these 517 Chinese landraces caused by the low-coverage sequencing depth (average 1× for each sample). *D*_*xy*_ between *indica* and *japonica* was about 0.00617 in our selected samples (Additional file 1: Figure S2), suggesting a significant differentiation between these two subspecies, which also had been confirmed by the PCA analysis. As expected, the diversity within these wild rice accessions was about 0.0067 (Additional file 1: Figure S2), which was significantly higher than that in *japonica* or *indica*, suggesting the strong bottleneck during the domestication that has also been reported by other studies [10, 23, 24]. As is known, the short-read sequencing technology could generate massive erroneous SNP calls if not properly handled. However, it was less likely for those false signals to mimic the truly differentiated SNPs. Such erroneous calls would distribute more randomly, both in *japonica* and in *indica*, which rarely resulted in low diversities within each subspecies. Furthermore, we did not observe any inflation in the estimated diversities, or any significant departure in the PCA analysis. This implies a conservative SNP call set, given the high coverage dataset and the stringent criteria used in this study.

### Detection of regions that underwent selective sweep

The large SNP data set detected in our samples provides an opportunity to identify artificial selected regions by comparing polymorphism levels in these cultivated and wild rice accessions. The selective signature from domestication with selective sweep includes a reduction in nucleotide diversity/divergence and altered allele frequency in these domestication loci. As mentioned above, the average diversity within *japonica* or *indica* is about 0.002. Therefore, the value of 0.002 was employed as one cutoff to detect the divergent regions between *japonica* and *indica*. Among these divergent regions, only those which have at least 10-fold lower diversity (0.0002) between the two subspecies can be defined as selective sweep regions both in *japonica* and *indica*. Compared with previous studies, two advantages can be found in our definition: i) the ten-fold-lower diversity is a more stringent criterion than previous study [10]_;_ ii) by using 0.002, we can easily exclude these regions having very low divergence between these two subspecies and very low diversity within each of the two subspecies, which may not be the result of divergence and selection, instead, caused by sequencing or analytical errors.

Therefore, two types of domesticated regions were detected (Tables 1, 2 and 3): domesticated region type I (defined as DR-I), which have undergone selective sweep within each subspecies (π ≤ 0.0002) but have high divergence between these two subspecies (*D*_*xy*_ ≥0.002); domesticated region type II (defined as DR-II), which have low diversity regions shared by both subspecies (not only π ≤ 0.0002 within each subspecies, but also *D*_*xy*_ ≤0.0002 between the two subspecies), but have high diversity within the wild rice (π ≥ 0.001) (See Methods for details).

**Table 1:** Blocks with selective sweep

	DR-I	DR-II
Block numbers	28	28
Average length (kb)	31.8	23
Total length (Mb)	0.89	0.64
Including genes	163	110

DR-I, domesticated regions of type I that have undergone selective sweep within each subspecies (π < 0.0002) but have high divergence between these two subspecies (*D*_*xy*_ >0.002); DR-II, domesticated regions of type II that have low diversity shared by both subspecies (not only π < 0.0002 within each subspecies, but also *D*_*xy*_ <0.0002 between the two subspecies), but have high diversity within the wild rice (π > 0.001)

**Table 2:** Genome-wide identification of selective sweep regions with low diversity (π ≤ 0.0002) within each of the two subspecies, respectively, but with high divergence (*D*_*xy*_ ≥0.002) between these two subspecies, which were defined as DR-I. Six regions showed slightly higher π values (≥0.0002 but ≤0.0003, flagged by * in this table) because these were merged regions (See methods)

	Chr	Range of the blocks	Length (bp)	π_*Jap*_ *10^5^	π_*Tro*_ *10^5^	π_*Tem*_ *10^5^	π_*Ind*_ *10^5^	*D*_*Jap-Ind*_ *10^5^	*D*_*Tro-Ind*_ *10^5^	*D*_*Tmp-Ind*_ *10^5^	π_*Wild*_ *10^5^	*D*_*Jap-Wild*_ *10^5^	*D*_*Ind-Wild*_ *10^5^
1	chr01	35499730–35510003	10274	4	2	6	14	242	246	241	89	178	121
2*	chr01	35839043–35890683	51641	19	10	13	21	468	478	467	232	320	263
3	chr02	13139975–13160003	20029	18	5	6	18	295	305	301	133	209	142
4	chr02	13349983–13360044	10062	13	2	4	11	241	249	246	101	149	131
5	chr02	14944936–14962544	17609	15	3	4	14	250	260	252	121	157	145
6	chr02	27708263–27721727	13465	11	4	11	14	263	271	263	87	143	145
7	chr03	1579192–1602557	23366	16	4	15	14	302	307	311	153	183	193
8*	chr03	2483329–2542460	59132	16	20	9	25	434	444	435	198	275	239
9	chr03	2706518–2724355	17838	13	18	7	18	440	453	440	188	310	210
10	chr03	2832279–2854311	22033	3	3	4	16	304	310	306	214	210	243
11	chr03	2896026–2923841	27816	5	3	5	15	321	327	323	151	178	220
12*	chr03	2997394–3195565	198172	18	11	22	21	578	590	580	260	356	316
13	chr03	3479756–3498823	19068	3	2	3	14	241	245	243	132	180	138
14*	chr03	24189350–24230136	40787	20	14	28	22	504	511	508	289	341	306
15	chr03	28469725–28489493	19769	14	21	7	12	429	437	427	245	327	308
16	chr04	34409908–34420007	10100	13	6	15	19	314	316	319	159	216	176
17	chr04	34469603–34500654	31052	14	6	16	16	262	264	265	123	169	136
18*	chr05	21659599–21670204	10606	16	7	25	21	289	295	291	156	177	202
19	chr05	22789981–22800012	10032	17	6	16	15	319	328	331	472	379	421
20	chr05	22869880–22880236	10357	6	2	3	17	295	305	302	137	155	208
21	chr05	24026607–24070766	44160	20	9	35	18	604	614	616	318	438	349
22	chr05	24300403–24330214	29812	16	8	13	11	343	356	350	348	346	329
23	chr05	26824033–26840483	16451	11	2	4	17	233	243	239	131	172	137
24	chr07	4149981–4163826	13846	9	8	2	18	214	220	217	420	365	365
25	chr07	25598750–25620217	21468	8	10	5	16	323	327	323	155	219	190
26	chr09	22759164–22771433	12270	6	1	3	18	251	258	254	171	164	204
27	chr09	22910542–22930021	19480	14	8	11	18	345	356	349	242	251	256
28*	chr10	21289972–21400480	110509	18	10	12	23	517	534	521	250	336	263
Average			31829	13	7	11	17	344	352	347	203	247	227

**Table 3:** Genome-wide identification of the DR-II type selective sweep regions with low diversity (π ≤ 0.0002) within cultivars, but high diversity (π >0.001) in wild rice

	Chr	Rang of blocks	Length (bp)	π_*Jap*_ *10^5^	π_*Ind*_ *10^5^	D_*Jap-Ind*_ *10^5^	π_*Cultivar*_ *10^5^	π_*Wild*_ *10^5^	D_*Jap-Wild*_ *10^5^	D_*Ind-Wild*_ *10^5^	D_*Cul-Wild*_ *10^5^
1	chr01	8645929–8660126	14198	1	18	16	13	118	104	108	106
2	chr03	26199939–26210003	10065	10	15	14	13	141	140	144	142
3	chr04	25959995–26040007	80013	10	15	14	13	181	228	229	229
4	chr04	26089892–26100078	10187	6	8	7	7	155	250	249	249
5	chr04	26229998–26260103	30106	10	15	15	14	230	327	325	326
6	chr04	26359991–26370003	10013	5	6	6	6	125	168	166	167
7	chr04	26789966–26810053	20088	10	16	14	14	155	251	247	249
8	chr04	26949945–26970083	20139	9	14	13	12	141	191	190	190
9	chr04	27009995–27060009	50015	7	20	17	16	286	360	356	358
10	chr04	32529159–32540000	10842	18	13	16	16	109	104	123	114
11	chr04	33889971–33900005	10035	15	10	20	16	173	228	222	225
12	chr04	33979989–33990038	10050	15	10	16	14	100	135	145	140
13	chr04	34219891–34249999	30109	12	7	12	11	277	316	319	318
14	chr05	29729953–29740204	10252	9	5	7	7	115	115	103	109
15	chr07	2739991–2770008	30018	15	13	18	16	406	456	451	453
16	chr07	2799992–2810002	10011	11	6	9	9	262	311	305	308
17	chr07	2879923–2910069	30147	14	10	13	13	280	437	432	434
18	chr07	2979996–2991842	11847	1	4	3	3	140	104	105	105
19	chr07	3096102–3109999	13898	10	9	11	10	110	116	108	112
20	chr07	3699995–3710053	10059	12	7	10	10	364	394	396	395
21	chr07	3759873–3790006	30134	9	7	10	9	264	273	272	273
22	chr07	4019981–4030093	10113	12	6	12	10	138	131	124	127
23	chr07	4070000–4090006	20007	9	17	15	14	236	256	252	254
24	chr08	23749999–23770091	20093	12	14	14	14	246	269	263	266
25	chr08	23809970–23860036	50067	9	16	15	14	549	500	501	501
26	chr08	23919999–23940003	20005	5	10	9	8	206	283	282	283
27	chr08	23989975–24040080	50106	7	14	11	11	273	315	312	313
28	chr12	24930000–24950016	20017	13	1	11	9	168	124	121	122
Average			22951	10	11	12	11	213	247	246	246

DR-I showed that differentiation has already occurred in these wild rice, and selective sweep was independently imposed on the domestication of *indica* or *japonica*, respectively. Therefore, the *indica*-*japonica* differentiation was driven not only by genetic drifts or possible natural selection of wild rice, but also by artificial selection of cultivated rice. The artificial selection should be much more powerful in accelerating the differentiation progress. For this type of domesticated regions, a total of 28 blocks, including about 890 kb of DNA (average: 31.8 kb; ranging from 10 kb to 198 kb) and 163 genes, were detected (Tables 1 and 2). Meanwhile, since *japonica* had been subdivided into *temperate japonica* and *tropical japonica* [25, 26], π within *temperate japonica* and *tropical japonica*, and *D*_*xy*_ between *temperate japonica* and *indica* and *tropical japonica* and *indica* were calculated (Table 2)*.* All the data showed a similar result, suggesting that the DR-I regions were conserved in *temperate japonica* and *tropical japonica*, and divergent between *temperate japonica* and *indica* or *tropical japonica* and *indica*.

For DR-II, normal diversity was detected within these wild rice accessions (π ≥ 0.001), whereas very low diversity was detected not only within each subspecies (π ≤ 0.0002), but also between the two subspecies (*D*_*xy*_ ≤ 0.0002), suggesting that selective sweep was simultaneously imposed on both *indica* and *japonica*, and these overlapping DR-IIs may have originated only once or as a result of selection and subsequent introgression from one subspecies to another. For this type of domesticated region, a total of 28 blocks, including about 640 kb genomic length (average: 23.0 kb, ranging from 10 kb to 80 kb), was detected (Tables 1 and 3).

### Origin of DR-I

**Fig. 1:**
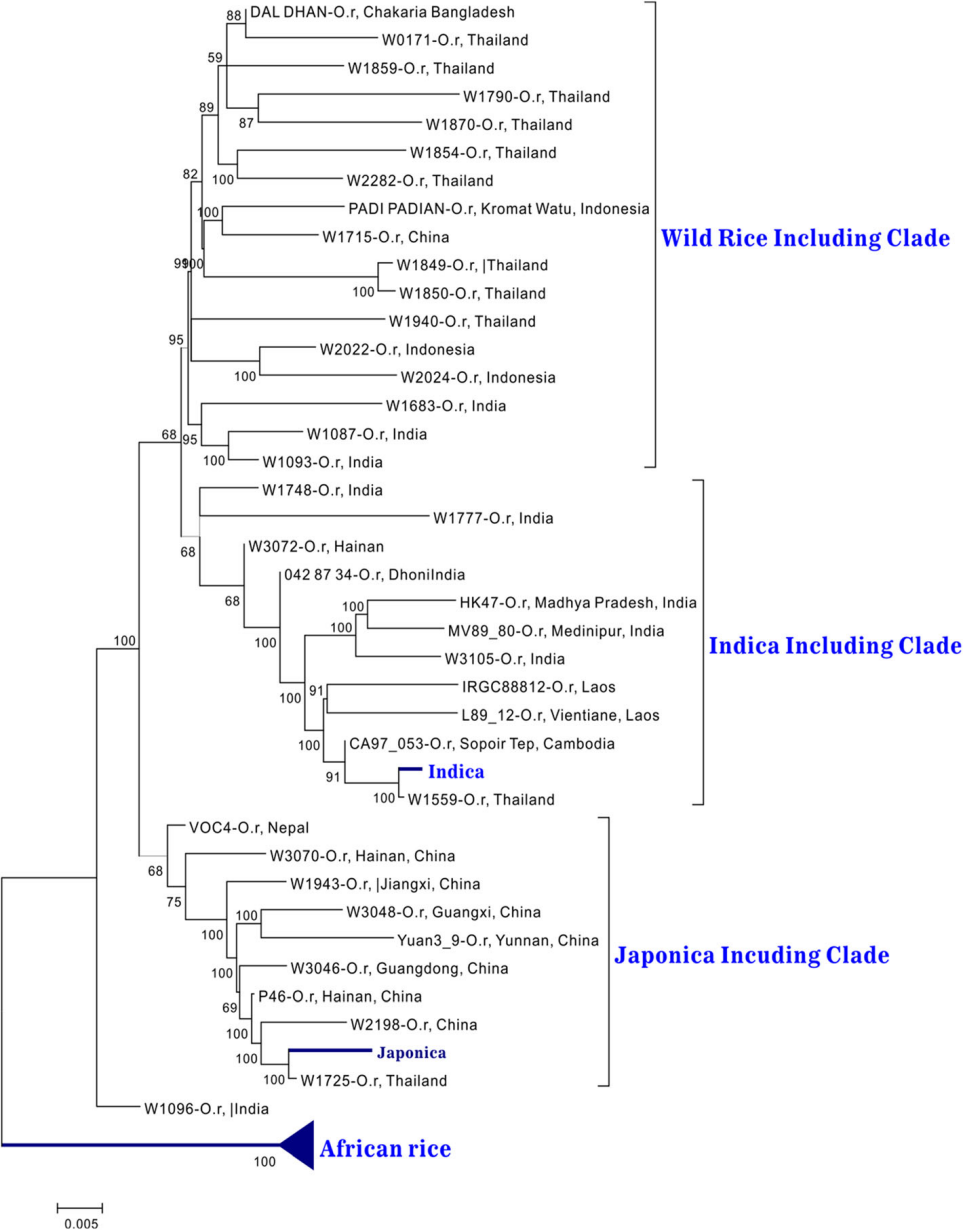
Phylogenetic tree of the 28 DR-I blocks. The low depth wild rice (whole genome depth <4) were not used in this tree

When using the SNPs of DR-I to reconstruct a phylogenetic tree, as expected, all *japonica* or *indica* samples clustered together within its own clade, respectively, whereas the *japonica* clade was distantly located from the *indica* clade (Fig. 1), which was consistent with the results of PCA analysis that the *japonica* varieties clearly segregate from the *indica* individuals (Additional file 1: Figure S1). Furthermore, Tajima’s *D* and F_st_ statistics also indicated significant differences of the genetic structure between *japonica* and *indica* populations in the DR-I region (Table 4). On the other hand, some wild rice accessions were clustered within the *japonica* or *indica* clade, respectively, and some wild rice accessions were scattered outside the two cultivated rice clades, which was also consistent with the findings of previous studies [10].

**Table 4:** Results of the analysis by Tajima’s *D* and F_st_ statistics between the *japonica* and *indica* populations in the DR-I region

	Chr	Range of the blocks	Tajima’s *D*			F_st_ (*Jap* vs *Ind*)
			*Jap.*	*Ind.*	Cultivar	
1	chr01	35499730–35510003	−2.344**	−2.319**	2.564**	0.93668**
2	chr01	35839043–35890683	−2.284**	−2.388**	1.470	0.88126**
3	chr02	13139975–13160003	−1.971**	−1.896	1.050	0.93705**
4	chr02	13349983–13360044	−1.940**	−2.265**	0.953	0.93638**
5	chr02	14944936–14962544	−1.871**	−1.518	1.551	0.8978**
6	chr02	27708263–27721727	−1.921**	−1.917**	2.404**	0.94298**
7	chr03	1579192–1602557	−1.794	−2.420**	1.862*	0.90987**
8	chr03	2483329–2542460	−2.171**	−1.616	1.587*	0.93704**
9	chr03	2706518–2724355	−2.379**	−2.458**	1.533*	0.84626**
10	chr03	2832279–2854311	−2.412**	−2.235*	1.182	0.58542
11	chr03	2896026–2923841	−2.343**	−2.108**	1.708*	0.90956**
12	chr03	2997394–3195565	−2.220**	−1.027	2.526**	0.94684**
13	chr03	3479756–3498823	−2.313**	−0.066	2.738**	0.9228**
14	chr03	24189350–24230136	−2.058**	−2.162**	1.219	0.92474**
15	chr03	28469725–28489493	−2.303**	−1.961**	1.236	0.8877**
16	chr04	34409908–34420007	−2.097**	−1.729	2.262*	0.91761**
17	chr04	34469603–34500654	−1.915**	−2.145**	1.701*	0.93408**
18	chr05	21659599–21670204	−1.757	−2.137**	0.900	0.89436**
19	chr05	22789981–22800012	−1.857	−2.009**	2.331**	0.91203**
20	chr05	22869880–22880236	−1.993**	−2.020**	2.582**	0.85427**
21	chr05	24026607–24070766	−2.116**	−1.458	2.510**	0.74986**
22	chr05	24300403–24330214	−2.017**	−2.275**	2.936**	0.94534**
23	chr05	26824033–26840483	−1.731	−1.795	2.738**	0.93111**
24	chr07	4149981–4163826	−1.766	−1.686	2.518**	0.92099**
25	chr07	25598750–25620217	−2.310**	−2.049**	2.955**	0.89888**
26	chr09	22759164–22771433	−2.131**	−2.444**	0.349	0.93668**
27	chr09	22910542–22930021	−1.892	−2.106**	2.634**	0.88126**
28	chr10	21289972–21400480	−2.120**	−1.634	2.594**	0.93705**

*and **indicate *P*-value < 0.05 and 0.01, respectively

In these 28 DR-I blocks, the average diversity was 0.00013 (ranging from 0.00003 to 0.00020) within *japonica* and 0.00017 (ranging from 0.00011 to 0.00025) within *indica* (Table 2). However, the average divergence between these two groups was 0.0034, which was 11- to 101-fold higher (~27-fold on average) than the diversity within each group (Table 2), suggesting significant differentiation between *indica* and *japonica* in these regions. The average diversity is ~15-fold lower within *japonica* and ~16-fold lower within *indica* than their corresponding genome-wide diversity, suggesting strong selection with selective sweep on these regions. On the other hand, in these blocks, significantly positive correlations were displayed either in the diversity within *indica* vs. *japonica*, or *D*_*ind-jap*_ vs. π_*jap*_ or π_*ind*_, or *D*_*jap-wild*_ and *D*_*ind-wild*_ (Additional file 1: Figure S3). This was consistent with the possibility that *japonica* and *indica* may have undergone strong artificial selection from different isolates of the wild rice *O. rufipogon* during domestication at least in these domesticated blocks.

For this reason, a phylogenetic tree was reconstructed using SNPs of the 28 DR-Is to determine which wild rice was more similar to the 28 domesticated blocks that were fixed or near fixed in *japonica* or *indica*, respectively*.* Similar to the findings of previous studies [10], four clear clades were detected, including the African rice clade (also as an outgroup clade), *japonica* clade with some *O. rufipogon* accessions, *indica* clade with some *O. rufipogon* accessions, and an independent *O. rufipogon* clade (Fig. 1). All *japonica* or *indica* samples were clustered together, whereas all *japonica* samples were far from all of these *indica* samples, which matched the PCA result that the *japonica* varieties clearly segregated from the other groups, suggesting that, at least in these 28 DR-I regions, these two subspecies were probably independently domesticated from different wild rice isolates. Interestingly, the wild rice accessions with the highest similarity to *japonica* varieties were W1725, W2198, P46 and W3046 (Fig. 1), which were collected from Thailand to Southern China, suggesting that these *japonica* domesticated regions may have independently originated from the regions of Southern China [10, 27] to the Indo-China Peninsula. On the other hand, the wild rice accessions with the highest similarity to *indica* were W1559, CA97, L89_12 and IRGC88812, all of which were collected from Indo-China Peninsula, suggesting that these *indica* domesticated regions may have independently originated from the regions of Indo-China Peninsula. Therefore, the Indo-China Peninsula might be the overlapping place with the closest wild relatives both for *japonica* and *indica*.

Due to limited sampling size of wild rice, one possible explanation for the topology of the phylogenetic tree was that the DR-Is are retained by frequent introgression from cultivars to some wild rice. To test this hypothesis, more wild rice samples are necessary for further investigation of the origin of these domesticated regions. We expect if most other wild rice accessions have a similar pattern with above regions, then we can exclude the introgression from cultivars to wild rice in these two regions. Conversely if there were a variety of wild rice accessions exhibiting dissimilar pattern in these two regions, then introgression from cultivars to wild rice may have an impact in our selected wild rice samples. Then, more than 400 whole-genome sequenced wild rice samples were collected to enlarge the sample panel, mostly having low sequence depth (~1×) (Additional file 1: Table S4). Additional file 1: Table S5 repeatedly shows that the wild rice accessions from the Indo-China Peninsula (e.g., W1725 and W1506 from Thailand) or Southern China (e.g., W3093, P46 and W3040) have the highest identity to the genotype of *japonica*, whereas most of the wild rice accessions from the Indo-China Peninsula (e.g., W1559, W1086 and W1930) have the highest identity to the genotype of *indica*. This is consistent with the above result that, at least in these 28 DR-I regions, the closest wild relatives for *japonica* and *indica* have the overlapping place in Indo-China Peninsula, suggesting that both of the two domesticated subspecies may have a common place of the origin.

### Origin of DR-II

In the 28 DR-II blocks, the average diversity was ~0.0001 either within *japonica* or *indica*, even in all of these cultivars, which was about 21-fold (from 6.1 to168-fold) lower than that of wild rice accessions (Table 3), suggesting strong selective sweep both in *indica* and *japonica* accessions. This result also suggested that these 28 DR-II blocks should have a common origin and subsequently introgressed from one subspecies to another [10]. The diversity in these regions within *japonica*, *indica*, or both was significantly lower than the diversity in the 28 DR-I blocks within *japonica* (*t-*test, *P* = 0.011) or *indica* (*t-*test, *P* < 0.0001), suggesting that the DR-I blocks may be independently fixed in *indica* or *japonica* prior to the fixation of the DR-II blocks.

**Fig. 2:**
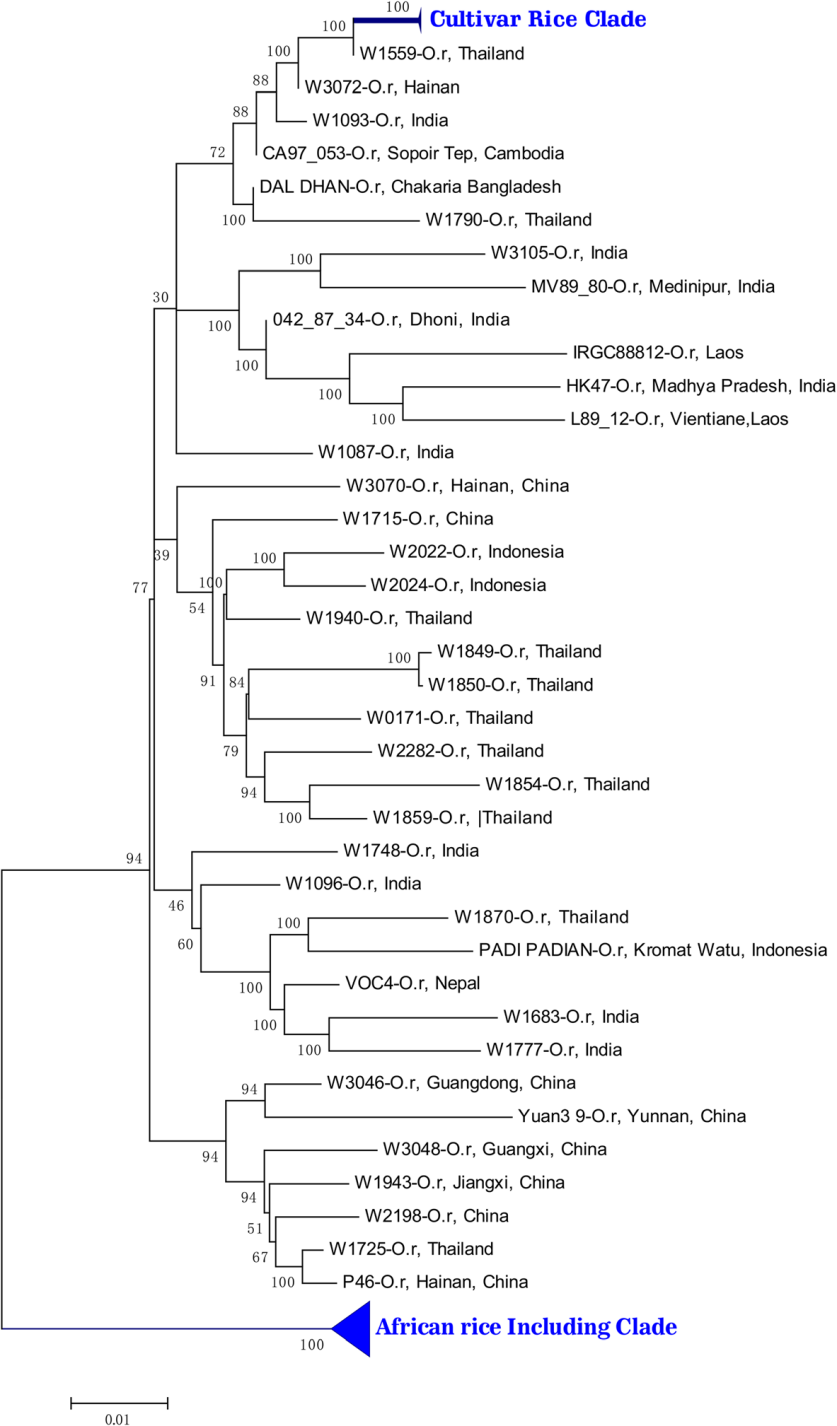
Phylogenetic tree of the 28 DR-II regions

A phylogenetic tree was also reconstructed using the SNPs from the 28 DR-II blocks. As expected, all *japonica* and *indica* samples were clustered together as a cultivar clade (Fig. 2), which was different from the topology of the DR-Is, in that tree *japonica* samples were distantly located from these *indica* samples and in different clades. The wild rice with the highest similarity to *japonica* and *indica* in this tree was also the wild rice of W1559, which had the highest similarity only to *indica* but not *japonica* samples in DR-Is, suggesting that these 28 DR-II blocks might have introgressed from *indica* to *japonica*.

To further investigate the direction of introgression in these DR-IIs, more whole-genome sequenced wild rice samples, including those with low depth, were used as earlier described. The wild rice accessions of W1086 (India), W0178 (Thailand), W1090 (India), CA97_053 (Cambodia), and W0639 (Burma) (Additional file 1: Table S6) have the highest identity to DR-IIs in both *indica* and *japonica*. On the other hand, all these wild rice accessions were clustered within the *indica*-type wild rice clade in the phylogenetic tree. This result further suggested that the 28 DR-IIs were introgressed from *indica* to *japonica*, which was contradictory to the reported 55 major domestication sweeps introgressed from *japonica* to *indica* [10], suggesting that not only introgression from *japonica* to *indica*, but also from *indica* to *japonica* could be detected in our rice cultivars.

### Functional classification and selection pressure of specific genes

In the DR-I and DR-II regions, 163 and 110 genes were detected, respectively (Additional file 1: Table S7 and S8). To determine the functional categories of these genes, we summarized the possible functions of these genes using rice Gene Ontology (GO) annotation (http://geneontology.org/) (Figure S4).

Most of the genes in the DR-I regions have a significant functional enrichment in cell growth, anatomical structure morphogenesis, cellular component organization, DNA metabolic process, reproduction, embryo development, and photosynthesis (Additional file 1: Figure S4); for example, some functions responding to seed or coleoptile development (*Os01g61380*, *Os03g05820*, *Os03g06010*-*Os03g06060*, *Os03g06120*, *Os03g06360*, *Os03g06890*, and *Os05g41030*), flower, pollen, or anther development (*Os03g05140*, *Os05g38990*, *Os07g08170*, and *Os10g39880*), root development (*Os03g43400* and *Os03g43410*) (Additional file 1: Table S7). This functional enrichment analysis was consistent with the findings of previous reports that the differentiation of morphological and physiological traits was an adaptation to distinct climatic, ecogeographic, and cultural conditions between *indica* and *japonica* [28–30].

Because the selective sweep was respectively detected in *indica* and *japonica*, and significant differentiation between these two subspecies was observed, we expected that more genes under positive selection would be detected in the genes of DR-I during the differentiation of *indica* and *japonica*. Therefore, the *Ka/Ks* between *indica* and *japonica* samples was employed to evaluate positive selection of these genes. Interestingly, 43 out of the 163 genes (25.9%) with *Ka/Ks* >1 (or *Ks* = 0, *Ka* > 0) were detected (Additional file 1: Table S7), which showed a significantly higher proportion (*χ* ^2^ with Yates correction = 32.4, d.f. = 1, *P* < 0.0001) than that (2,678 out of 27,384, ~8.7%) of the genome-wide reports [7], suggesting that a large number of the DR-I genes were under positive selection during the differentiation of *indica* and *japonica*, and the differentiation of the two subspecies was driven by both artificial and natural selection with strong positive selection, which directly acted on many characteristics.

Genes in the DR-II regions were enriched in cell differentiation, anatomical structure morphogenesis, cell growth, photosynthesis, awns development, flower development, cell cycle, embryo development, and reproduction (Additional file 1: Figure S5 and Table S8). This functional enrichment was consistent with the reports on the differentiation of morphological and physiological traits for cultivars and wild rice. For example, *sh4* ^19^, a gene related to shattering, which was included in the selective sweep regions in the research of Huang et al. [10], was also found in our DR-II. Remarkably, a more recent research reported a highly conserved region in chromosome 4, which was also included in our DR-II regions [26]. *LABA1* (LOC_Os04g43840) and *GAD1* (LOC_Os08g37890) were also detected in our results, which were associated with long, barbed awns in wild rice (Table S8) [31, 32].

## Discussion

There is currently an ongoing debate both in the genetic and archaeological arenas on the origins of Asian cultivated rice [27]. Numerous studies have suggested that *indica* and *japonica* may have multiple origins and that the two subspecies might have been independently domesticated from different isolates of the wild rice of *O. rufipogon* [8, 27], and *indica* might have originated in eastern India and *japonica* from southern China [8]. However, a recent report has shown that a single origin for domesticated Asia rice was more likely to have occurred than multiple origins, as indicated by the results of Bayesian phylogenetic analyses [9]. Interestingly, a more recent study based on genome-wide variation has reported that *japonica* might have been first domesticated from wild rice of Or-III in southern China, and was subsequently followed by dispersal to Southeast Asia, and then crossed with local wild rice of Or-I to generate *indica* after several cross-differentiation-selection cycles [10]. Another model was recently proposed by Choi et al., in support of separate origins for different rice subspecies, but only a single de novo domestication of rice which occurred in *japonica*. [33]. Other studies have shown that the domestication process of rice might have been initiated multiple times, followed by extensive introgression of strongly selected alleles, e.g., some important domesticated genes of *sh4*, *rc*, and *waxy*, which originated in *japonica* and spread to *indic*a [19, 34, 35]. Therefore, frequent introgression between *indica* and *japonica* is a widely accepted theory for the domestication of rice [27]. However, the geographical discordance relating to the concept that *japonica* was domesticated in Southern China, and *indica* was generated in Southeast Asia or South Asia by *japonica* crossing with local wild rice after its dispersal remains elusive.

Interestingly, our data have shown that *japonica* and *indica* may have a common place of the origin in Indo-China Peninsula in the DR-I and DR-II, which have the highest identity to that of the wild rice from Indo-China Peninsula and South Asia, suggesting that all these regions with strongly selective sweep may have a common place with the highest identity for some accessions of the wild rice. This result was consistent with those of a recently published study [26]. On the other hand, our data also showed that the average diversity in the DR-II regions either within *japonica*, *indica*, or both was significantly lower than that in the DR-I blocks within *japonica* (*t-*test, *P* = 0.011) or *indica* (*t*-test, *P* < 0.0001) (Tables 2 and 3), suggesting that the DR-I blocks were independently fixed in *indica* or *japonica* prior to the fixation of these DR-II blocks. Third, our data also showed that all of the 28 DR-IIs were introgressed from *indica* to *japonica*, which was inconsistent with the findings of previous reports in that 55 major domestication sweeps might have introgressed from *japonica* to *indica* [10], suggesting that not only introgression from *japonica* to *indica* but also from *indica* to *japonica* can be detected in the rice cultivars.

**Fig. 3:**
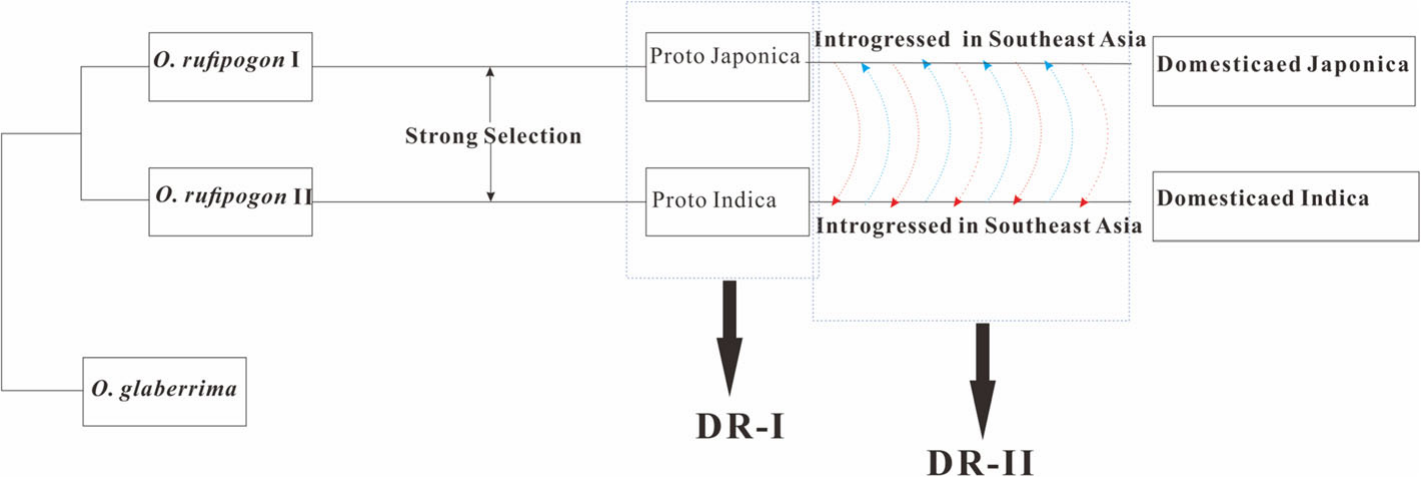
The model of the origin of the cultivar rice *japonica* and *indica*. In this model, the modern *japonica* and *indica* were domesticated from different wild rice

**Fig. 4:**
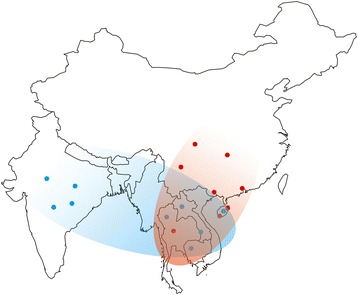
Origin place of the wild rice selected as the *Japonica*-clade (*red dots*) and *Indica*-clade (*blue dots*) by DR-I. As shown in this figure, the two clades overlapped in Southeast Asia. A geographical map was created using maps packages [51] (version 3.0) from CRAN (https://cran.r-project.org/web/packages/maps/index.html)

Therefore, a possible model of the origin of the cultivated rice (Fig. 3) may be that (i) the proto-*indica* and proto-*japonica* might have independently originated from the divergent wild rice in the Indo-China Peninsula, in which the DR-Is with significant divergence between the two subspecies may have been domesticated during this period; (ii) then, followed by frequent introgression from *indica* into proto-*japonica* also in the Indo-China Peninsula (detected in this study), or from *japonica* into proto-*indica* in southern China [10, 27], modern *japonica* and *indica* formed and spread to different regions. During this period, DR-IIs may have been domesticated in all these cultivated rice (Fig. 4).

Rice have undergone a series of similar phenotypic domestication for effective harvest and planting from their wild progenitors, including a reduction in seed shattering and dormancy, synchronization of seed maturation, increase in seed sizes, and decrease in culm number and branches [11, 19, 36–40]. On the other hand, the two major rice subspecies are differentiated by a number of morphological and physiological characters, along with a substantial sterility barrier [41]. Therefore, we expected that the genes with strongly selective sweep should have functional bias.

Interestingly, for the genes found in DR-I regions, on one hand, a significant functional bias was detected in seed or coleoptile development, flower, pollen or anther development, and root development (Additional file 1: Table S7), which was consistent with the differentiation of the morphological and physiological traits, e.g., seed size, between the two subspecies. On the other hand, a large number of these domesticated genes were detected to have undergone positive selection, suggesting that the differentiation of the two subspecies was driven by both natural and artificial selection with strong positive selection in these domesticated genes.

Furthermore, some genes under strong positive selection had a tendency of being clustered within small regions. For instance, a 0.6-Mb block containing 2 DR-Is showed a significantly lower diversity in *japonica* than *indica*, which strongly implied selection sweep and genetic bottleneck (Additional file 1: Figure S6). Within this block, 8 genes (*Os03g05820*, *Os03g06010*–*Os03g06060*, and *Os03g06120*), which may be related to root development and coleoptile development, were clustered [29–31]. In addition, not only these 8 genes, but also the nearby genes were under a significantly positive selection (Additional file 1: Table S7), although the function of the other genes was unclear or apparently showed no relationship with the differentiation of *japonica* and *indica*. Hitchhiking effect may explain such a high positive selection of this region, or that the genes with unknown functions may have played major roles in the differentiation of the two rice subspecies. Similar results were observed in DR-II (Additional file 1: Figure S7 and Table S8), suggesting that these regions may contain a large number of important genes that were related to the domestication of the cultivated rice. Coincidentally, two clustered regions in chr04: 26.0 Mb–27.1 Mb and chr08:23.7 Mb-24.9 Mb were also detected by Hua et al. [31] and Jin et al. [32]. Thus, the present research not only determined the origin of the Asian rice cultivars in higher detail, but also facilitated in the discovery of more genes relative to domestication and breeding. Our method could be applied to molecular breeding and agricultural schemes of various rice cultivars.

We need to notice that, although the samples were only selected according to the coverage threshold, we could not totally rule out sampling bias. In this study, we only focused on those most highly-differentiated *indica* and *japonica* accessions, this could be failed to reflect the truly diversity between *indica* and *japonica*. The somewhat controversial conclusions from different researches [10, 33] might in turn reflect the importance of sampling strategy. However, as we are not going to draw a final conclusion on the debate of rice domestication, we called for more extensive samplings to further test the generalizability of our conclusions.

## Conclusion

By using 296 whole-genome sequenced rice cultivars in 3000 rice project and 39 diverse wild rice of *O. rufipogon*, our research had detected 28 DR-Is and 28 DR-IIs, which covered 890 kb and 640 kb regions, and harbored 163 and 110 genes, respectively. The results suggested that both DR-Is and DR-IIs might have originated from the Indo-China Peninsula to southern China. In addition, functional bias with significant positive selection has been detected in the genes of DR-I, suggesting that the selective sweep with significant positive selection might drive for the differentiation of the *japonica* and *indica* rice subspecies.

## Methods

### Data source

The resequencing data of *japonica* and *indica* individuals was obtained from the 3,000 rice project ^21^ (Additional file 1: Table S1). For wild rice (*O. rufipogon*), 371 rice samples were obtained from Huang et al. [10], 10 were obtained from Xu et al. [25], one was obtained from Ohyanagi et al. [42] and one was obtained from Zhang et al. [43] (Additional file 1: Table S3). The resequencing data of 20 African domesticated rice (*O. glaberrima*) were collected from Wang et al. [44] (Additional file 1: Table S3).

### Analysis of sequencing data

All available *indica* and *japonica* individuals were selected for further analysis based on the sequencing depth (*japonica* ≥15× and *indica* ≥20×). All reads were mapped against the reference genome *Nipponbare* (IRGSP1.0, http://rice.plantbiology.msu.edu/) using BWA-mem (version 0.7.9a-r786) [45] with option “-M”. Picard-MarkDuplicates (version 1.114, http://broadinstitute.github.io/picard/) and GATK-IndelRealigner (version 3.2) [46] were applied to correct mapping results. GATK-UnifiedGenotyper was applied to identify SNPs (single nucleotide polymorphism) of each rice individual. To reduce possible sequencing and mapping errors, SNPs with poor quality (quality <50), low depth (depth < 3) or low frequency (MAF < 5%) were excluded. These retained SNPs were then used for further analysis.

### Selection of *japonica* and *indica* samples

Nucleotide diversity (π) or divergence (*Dxy*), which was defined as the ratio between SNP numbers and the corresponding region length [47], served as criterion for the selection of *indica* and *japonica* individuals. For example, as to one *japonica* individual, we defined π_1_ as the average π value between this individual and all the other *japonica* samples. Similarly, *Dxy*_1_ was the average *Dxy* value between this *japonica* individual and all the *indica* samples. When π_1_ ≥ *Dxy*_1_, this *japonica* individual was considered to be a non-typical *japonica* rice and excluded from the analysis. Finally, principal components analysis (PCA) was also performed with VCFtools (http://vcftools.sourceforge.net/) and GCTA (http://cnsgenomics.com/software/gcta/), using all the SNPs in the whole genome. The first two components of the PCA analysis were adapted to further remove the *japonica*/*indica* individuals that were not clustered.

### Detection of the differentiated/domesticated regions

All the genomes were divided into 37,332 10-kb-windows and π/*Dxy* was applied as the genetic parameter to identify domesticated regions. For each window, when the π values within *japonica* or *indica* individuals were both ≤0.0002, but the *Dxy* between *japonica* and *indica* was ≥0.002, we defined this region of this window as part of the domesticated region type I (DR-I). When the cultivar individuals had a low diversity (π values within the *japonica* and *indica* individuals were both ≤0.0002, and *Dxy* between *japonica* and *indica* was also ≤0.0002), whereas the π within the wild individuals was ≥0.001, we selected this window as part of domesticated region type II (DR-II). Furthermore, using IGV [48], regions with low mapping depth, or low mapping quality, or obvious translocation, or high proportion of repeat sequence, were discarded. In addition, adjacent uniform-type domesticated regions were merged into one block, even when the π value of a part of the block might be a bit higher than 0.0002. Finally, these blocks were used for further analysis as completed domesticated regions (DR-I or DR- II).

### Calculation of the genetic parameters

For all the SNPs within the entire genome of selected *japonica* and *indica* individuals, when most (≥80%) *japonica* individuals shared one genotype whereas most (≥80%) *indica* individuals shared another genotype, we defined this SNP site as a near-fixed marker between *japonica* and *indica*. Then, using these near-fixed markers, non-synonymous (*Ka*) or synonymous (*Ks*) substitutions of the coding sequences between *japonica* and *indica* were calculated using the Nei-Gojobori method with Jukes-Cantor correction. Whole-genome F_st_ values and Tajima’s *D* indices were calculated by VCFtools. Calculation of the F_st_ and its significance in domesticated regions were performed by Arlequin31 [49]. A 5% confidence interval within the whole rice genome was used to identify significance of Tajima’s D.

SNPs in DR-I or DR-II were used to build corresponding neighbor-joining (NJ) trees with Jukes-Cantor model using MEGA v5.0 [50]. Bootstrap analysis with 1,000 replicates was used to estimate the stability of internal nodes and gaps/missing data treatment was performed to identify pairwise deletions.

## Additional file

Additional file 1: Figure S1. PCA plots of the first two components before (a) and after (b) sample selection. **Figure S2.** The proportions of the genome-wide diversity within the groups of *japonica*, *indica* and wild rice and divergence between *japonica* and *indica* group. **Figure S3.** Diversity/divergence relationship between rice groups. **Figure S4.** GO statistic of the DR-I regions. **Figure S5.** GO statistic of the DR-II regions. **Figure S6.** Clustered regions of DR-I. **Figure S7.** Clustered regions of DR-II. **Table S1.** List of 330 rice cultivars downloaded from the 3 K-rice project. **Table S2.** PCA value for each sample. **Table S3.** List of African cultivated rice (*O. glaberrima*) and wild rice (*O. rufipogon* and *O. nivara*) used to generate phylogenetic tree. **Table S4.** List of wild rice obtained from Huang et al. used in this project. **Table S5.** SNP genotype of DR-I. **Table S6.** SNP genotype of DR-II. **Table S7.** List of 163 genes in the 28 DR-I regions. **Table S8.** List of 110 genes in the 28 DR-II regions. (PDF 2465 kb)

## Abbreviations

DR-I: Domesticated regions of type I
DR-II: Domesticated regions of type II
GO: Gene Ontology
PCA: Principle component analysis
SNP: Single nucleotide polymorphism
