# Supplementary material for: Selective sweep with significant positive selection serves as the driving force for the differentiation of japonica and indica rice cultivars
Source: BMC Genomics. 2017 Apr 19;18:307. doi: 10.1186/s12864-017-3702-x (PMC5395770; doi:10.1186/s12864-017-3702-x)
Supplement: Additional file 1: Figure S1. — PCA plots of the first two components before (a) and after (b) sample selection. Figure S2. The proportions of the genome-wide diversity within the groups of japonica, indica and wild rice and divergence between japonica and indica group. Figure S3. Diversity/divergence relationship between rice groups. Figure S4. GO statistic of the DR-I regions. Figure S5. GO statistic of the DR-II regions. Figure S6. Clustered regions of DR-I. Figure S7. Clustered regions of DR-II. Table S1. List of 330 rice cultivars downloaded from the 3 K-rice project. Table S2. PCA value for each sample. Table S3. List of African cultivated rice (O. glaberrima) and wild rice (O. rufipogon and O. nivara) used to generate phylogenetic tree. Table S4. List of wild rice obtained from Huang et al. used in this project. Table S5. SNP genotype of DR-I. Table S6. SNP genotype of DR-II. Table S7. List of 163 genes in the 28 DR-I regions. Table S8. List of 110 genes in the 28 DR-II regions. (PDF 2465 kb) [file 12864_2017_3702_MOESM1_ESM.pdf]

Selective sweep with significant positive selection serves as the driving force for the differentiation of *japonica* and *indica* rice cultivars

Yang Yuan[1,2,4], Qijun Zhang[3,4], Shuiyun Zeng[2,4], Longjiang Gu[2], Weina Si[2], Xiaohui Zhang[2], Dacheng Tian[2], Sihai Yang[2,*], Long Wang[2,*]

[1] The Applied Plant Genomics Laboratory, College of Agricultural Sciences, Nanjing Agricultural University, Nanjing, 210095, China

[2] State Key Laboratory of Pharmaceutical Biotechnology, School of Life Sciences, Nanjing University, Nanjing 210023, China

[3] Jiangsu Academy of Agricultural Sciences, Nanjing 210000, China.

[4] These authors contributed equally to this work

Figure S1 PCA plots of the first two components before (a) and after (b) sample selection.

(a)

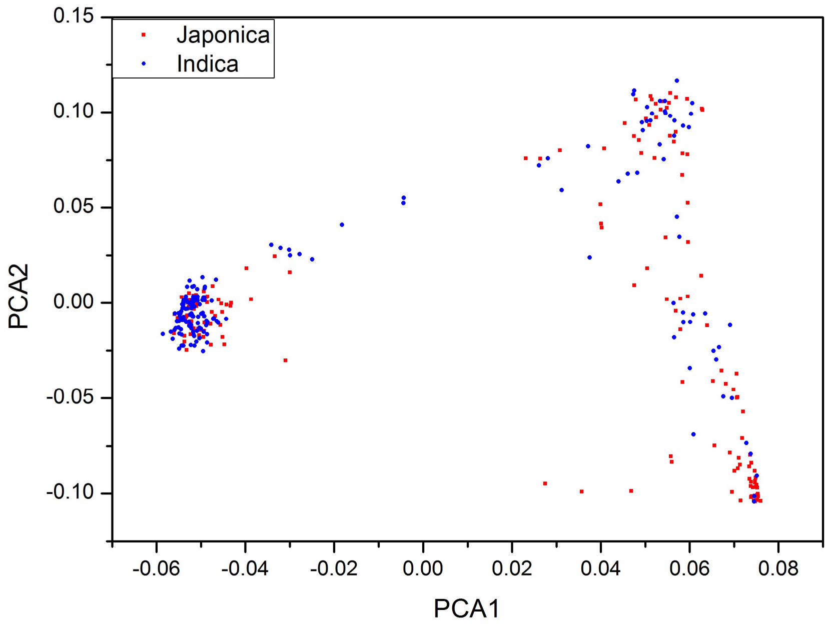

(b)

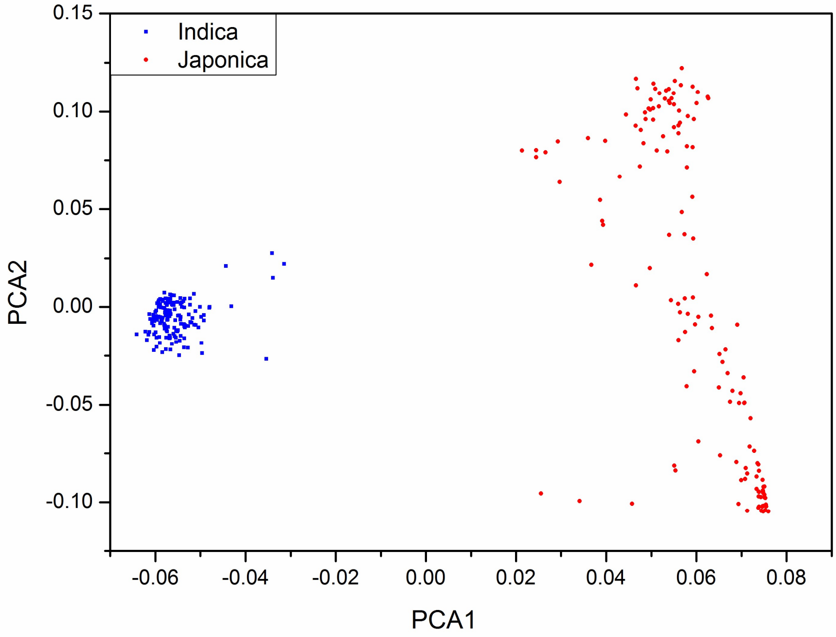

Figure S2 The proportions of the genome-wide diversity within the groups of *japonica*, *indica* and wild rice and divergence between *japonica* and *indica* group.

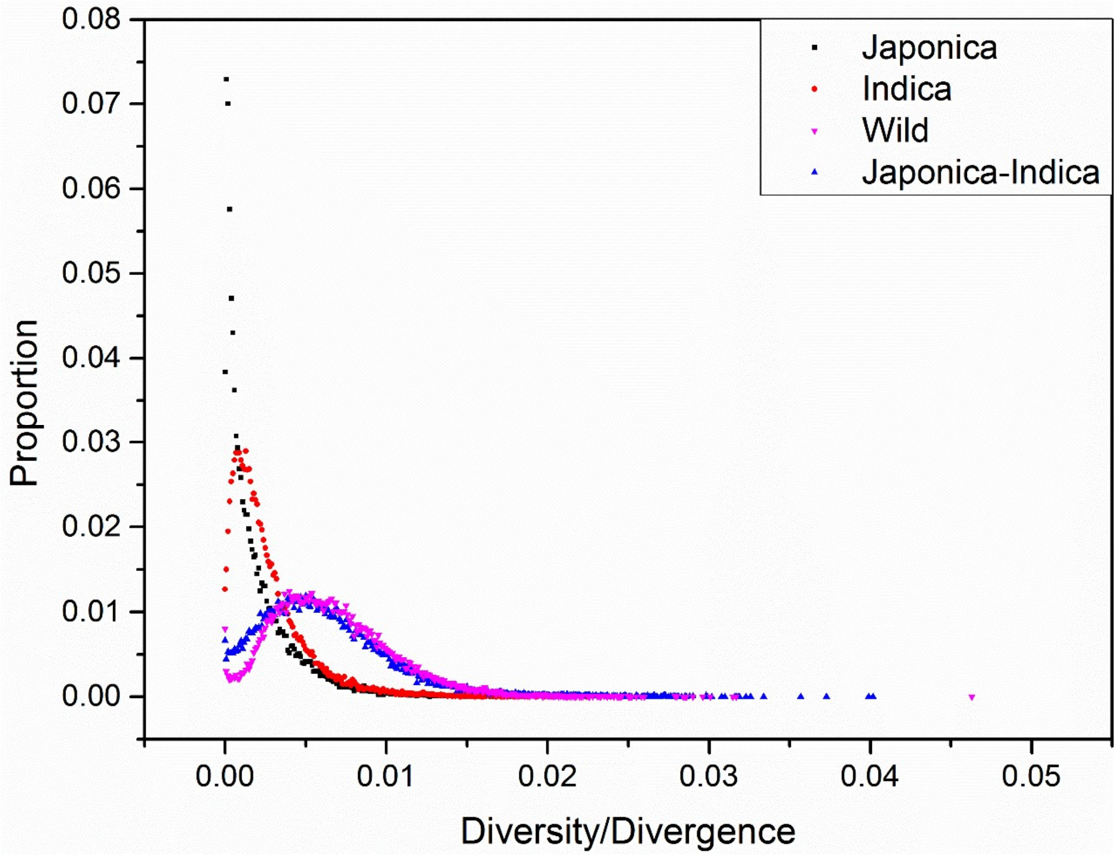

Figure S3 Diversity/divergence relationship between rice groups. (a) Relationship of the diversity within *indica vs. japonica*; (b) Relationship of diversity within *japonica vs.* divergence between *indica* and *japonica*; (c) Relationship of diversity within *indica vs.* divergence between *indica* and *japonica*; (d) Relationship of divergence between $D_{jap\text{-}wild}$ and $D_{ind\text{-}wild}$.

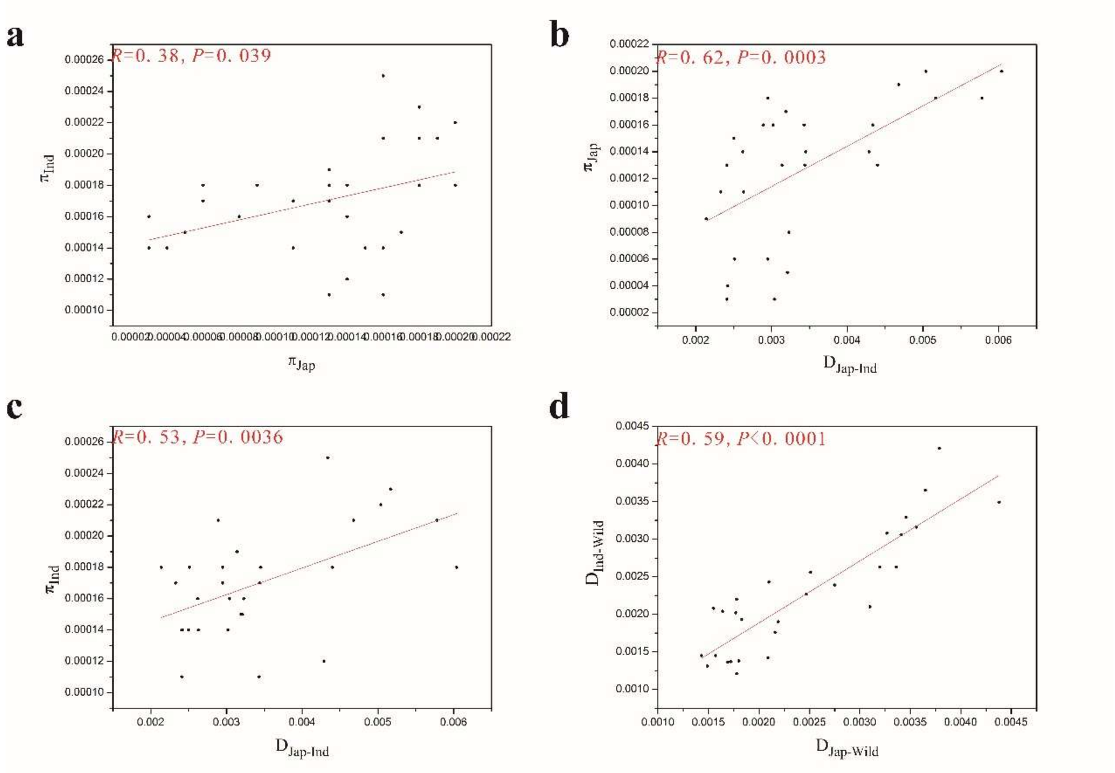

Figure S4 GO statistic of the DR-I regions. The X-axis showed different GO functions and the Y-axis represents the proportion of the regional counts of selected GO function to the whole genome counts. Chi-square test was applied and * stands for FDR<0.05 and ** stands for FDR<0.01.

The words in x-axis were short description of GO numbers. For instance, “Behavior” is the short description of GO:0007610, which means behavioral response to stimulus or behavior, behavioral response to stimulus. The same to **Supplementary Figure S5**.

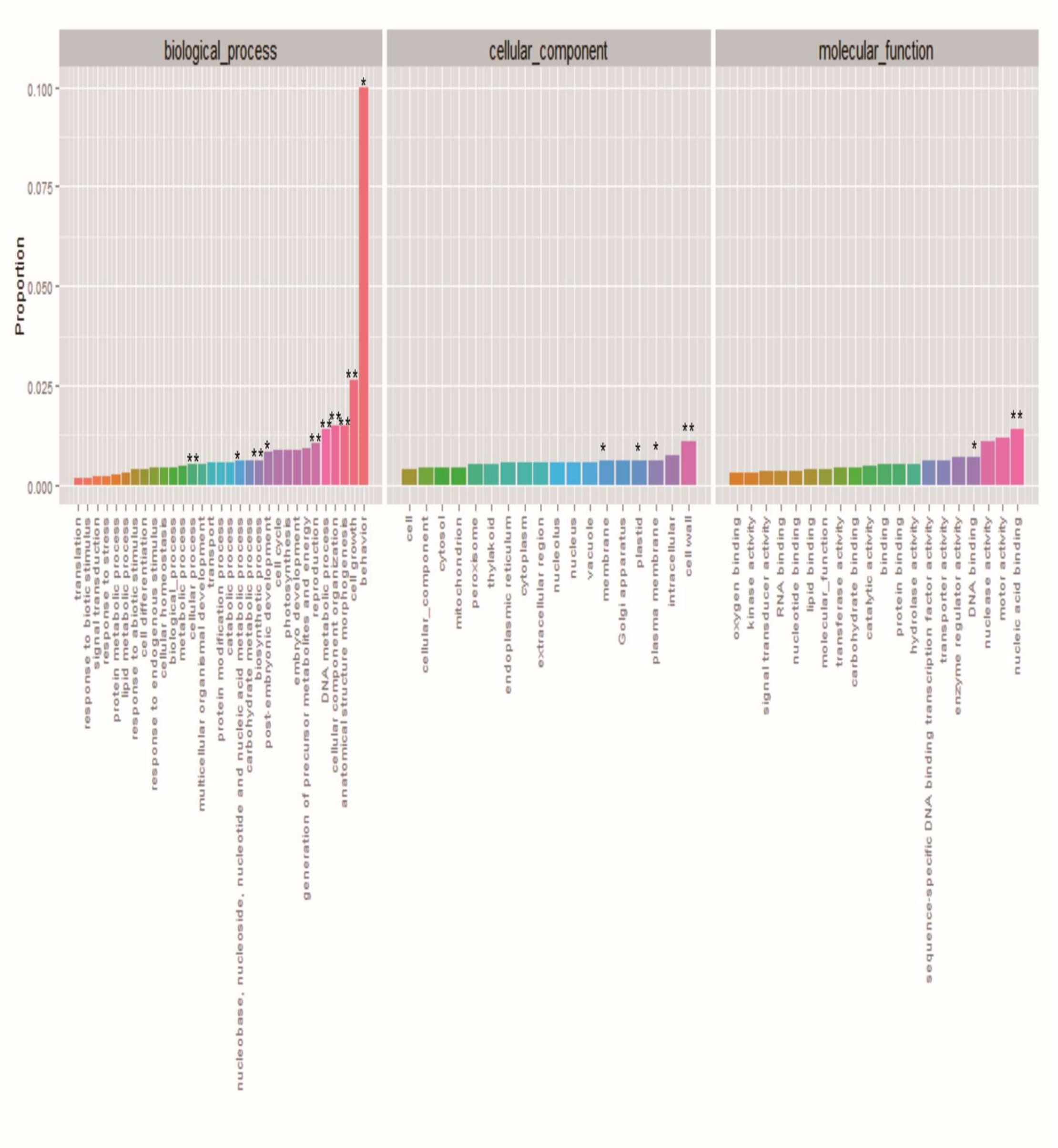

Figure S5 GO statistic of the DR-II regions. The X-axis showed different GO functions and the Y-axis represents the proportion of the regional counts of selected GO function to the whole genome counts. Chi-square test was applied and * stands for FDR<0.05 and ** stands for FDR<0.01

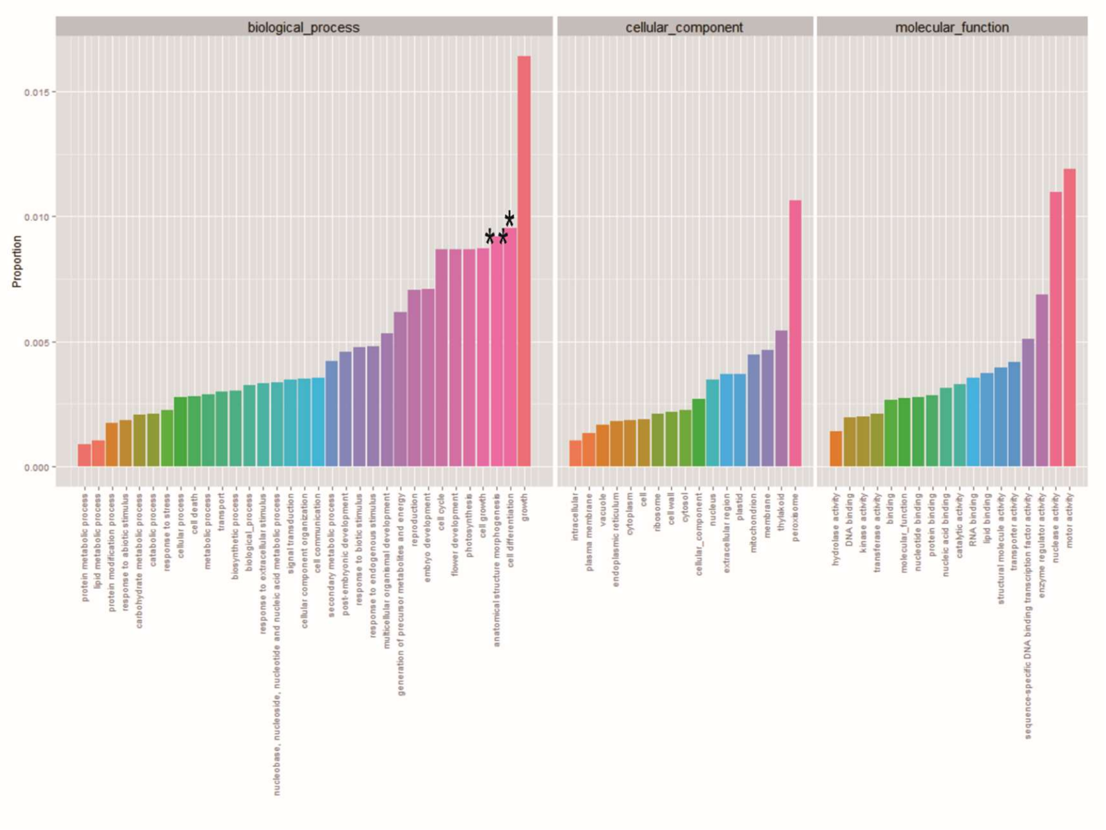

Figure S6 Clustered DR-I regions in chr03. The arrows showed the selected DR-I regions in this research. The dotted line showed the value of π = 0.0002.

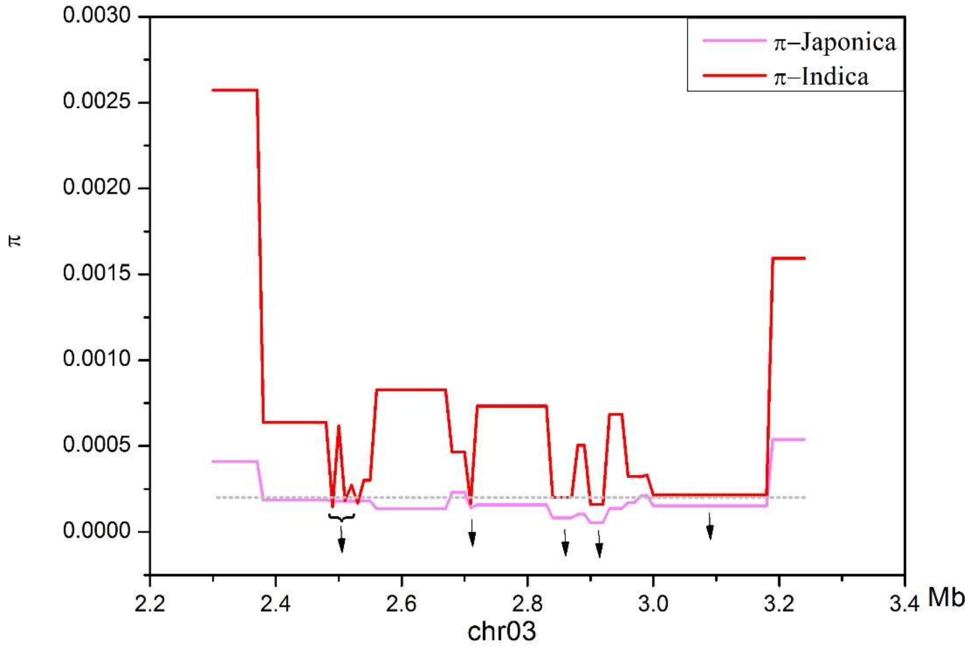

Figure S7 Clustered regions of DR-II. Grey line represents the π value equals 0.0002. The arrows showed the selected regions. The dotted line showed the value of π= 0.0002 (a) 7 regions clustered in chr04:26.0-27.1Mb. (b) Nine regions clustered in chr07:2.7-4.1Mb

(a)

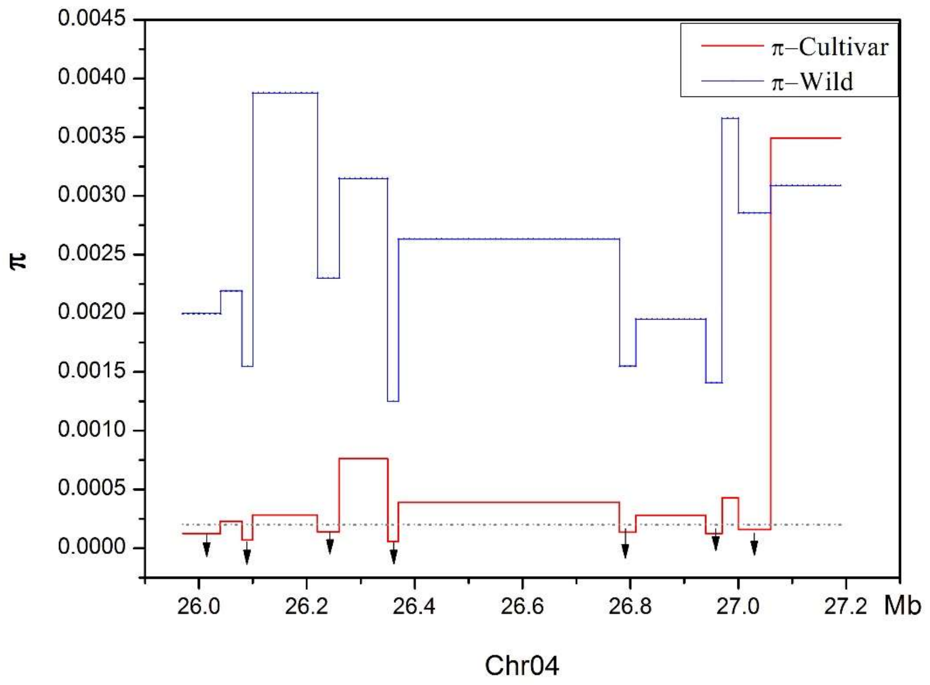

(b)

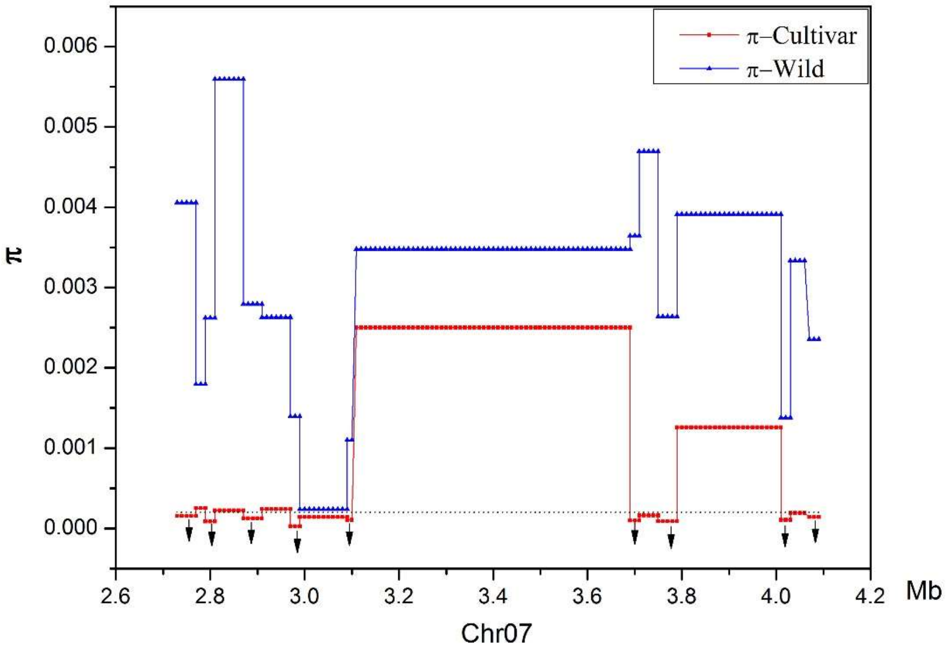

π–Cultivar
π–Wild
π
0.006
0.005
0.004
0.003
0.002
0.001
0.000
2.6
2.8
3.0
3.2
3.4
3.6
3.8
4.0
4.2
Mb
Chr07

Table S1 List of 330 rice cultivars downloaded from the 3K-rice project. In this list, only 296 cultivars were used in this project and 34 cultivars were removed after diversity and PCA analysis.

Sample ID	Variety Group	Area of origin	Depth	Coverage[1]
List of the 296 rice cultivars used in this project				
B001	Temperate_Japonica	China	27.59	0.94
B003	Temperate_Japonica	China	16.66	0.86
B005	Temperate_Japonica	Japan	17.06	0.91
B008	Temperate_Japonica	Vietnam	15.32	0.9
B012	Indica	India	24.14	0.86
B015	Indica	Romania	22.71	0.85
B016	Temperate_Japonica	Hungary	19.47	0.9
B017	Temperate_Japonica	Bulgaria	18.28	0.9
B018	Tropical_Japonica	United States	22.34	0.9
B023	Temperate_Japonica	North Korea	15.96	0.89
B026	Indica	Indonesia	22.79	0.85
B034	Temperate_Japonica	Albania	17.21	0.91
B036	Temperate_Japonica	Brazil	17.55	0.88
B043	Tropical_Japonica	Australia	15.23	0.87
B045	Temperate_Japonica	Japan	20.11	0.92
B046	Temperate_Japonica	Japan	19.53	0.92
B066	Temperate_Japonica	China	18.96	0.89
B070	Temperate_Japonica	China	20.76	0.89
B097	Indica	China	24.8	0.86
B100	Temperate_Japonica	China	17.35	0.89
B111	Temperate_Japonica	China	15.99	0.87
B143	Japonica	China	15.5	0.87
B160	Temperate_Japonica	China	22.43	0.9
B162	Temperate_Japonica	China	22.69	0.89
B167	Temperate_Japonica	North Korea	19.33	0.89
B182	Temperate_Japonica	Japan	24.85	0.9
B183	Temperate_Japonica	Japan	19.81	0.92
B190	Tropical_Japonica	Nigeria	21.76	0.88
B191	Tropical_Japonica	Australia	20.04	0.88
B196	Japonica	China	15.64	0.84
B204	Temperate_Japonica	China	27.22	0.91
B210	Indica	China	25.54	0.84
B212	Temperate_Japonica	China	23.95	0.89
B214	Indica	China	27.48	0.85
B218	Temperate_Japonica	China	16.62	0.89
B225	Temperate_Japonica	China	17.81	0.89

B226	Temperate_Japonica	China	19.01	0.89
B230	Temperate_Japonica	China	16.3	0.87
B241	Tropical_Japonica	China	16.54	0.87
B250	Temperate_Japonica	China	21.76	0.92
B258	Temperate_Japonica	China	16.52	0.88
B261	Indica	China	22.86	0.85
B264	Indica	China	28.91	0.86
B265	Indica	China	32.13	0.87
B266	Tropical_Japonica	China	15.28	0.86
B269	Temperate_Japonica	China	29.58	0.94
CX101	Indica	Taiwan	23.07	0.85
CX102	Indica	Taiwan	22.29	0.85
CX106	Tropical_Japonica	Vietnam	21.69	0.91
CX109	Japonica	Philippines	26.55	0.91
CX111	Tropical_Japonica	Egypt	20.8	0.9
CX138	Japonica	China	19.12	0.9
CX139	Japonica	Portugal	15.11	0.83
CX142	Temperate_Japonica	China	20.64	0.9
CX147	Indica	-	22.8	0.85
CX150	Indica	Philippines	21.94	0.84
CX152	Indica	-	23.49	0.85
CX154	Indica	Thailand	22.85	0.85
CX155	Indica	India	23.24	0.85
CX16	Temperate_Japonica	China	15.22	0.83
CX205	Japonica	Philippines	16.5	0.85
CX220	Tropical_Japonica	Brazil	22.5	0.9
CX225	Indica	Philippines	23.94	0.87
CX241	Japonica	Philippines	15.31	0.82
CX243	Japonica	Philippines	17.81	0.86
CX262	Japonica	China	19.83	0.88
CX269	Tropical_Japonica	Brazil	20.89	0.89
CX285	Japonica	China	18.86	0.89
CX3	Indica	Bangladesh	22.06	0.85
CX306	Temperate_Japonica	China	17.42	0.89
CX315	Temperate_Japonica	North Korea	16.94	0.88
CX329	Temperate_Japonica	China	15.02	0.88
CX353	Japonica	China	21.56	0.88
CX354	Temperate_Japonica	China	16.48	0.85
CX355	Japonica	China	20.03	0.86
CX383	Temperate_Japonica	China	19.57	0.91
CX389	Japonica	China	24.74	0.91
CX391	Temperate_Japonica	China	15.62	0.87
CX98	Indica	India	24.71	0.86

CX99	Indica	India	24.78	0.86
IRIS_313-10010	Indica	Fiji	23.45	0.86
IRIS_313-10097	Temperate_Japonica	South Korea	23.44	0.94
IRIS_313-10171	Indica	China	25.92	0.87
IRIS_313-10177	Indica	China	33.67	0.88
IRIS_313-10235	Indica	Philippines	23.59	0.87
IRIS_313-10440	Temperate_Japonica	Philippines	16.21	0.89
IRIS_313-10511	Indica	Philippines	22.7	0.86
IRIS_313-10541	Tropical_Japonica	Guinea-Bissau	17.03	0.89
IRIS_313-10563	Temperate_Japonica	-	17.68	0.9
IRIS_313-10564	Temperate_Japonica	Japan	16.71	0.93
IRIS_313-10570	Temperate_Japonica	Japan	15.37	0.91
IRIS_313-10577	Tropical_Japonica	Philippines	23.08	0.91
IRIS_313-10578	Tropical_Japonica	Philippines	17.57	0.88
IRIS_313-10582	Tropical_Japonica	Philippines	25.49	0.91
IRIS_313-10609	Indica	Sri Lanka	26.23	0.87
IRIS_313-10617	Temperate_Japonica	-	15.53	0.89
IRIS_313-10642	Temperate_Japonica	Japan	20.68	0.89
IRIS_313-10657	Tropical_Japonica	Laos	18.22	0.9
IRIS_313-10677	Temperate_Japonica	Japan	15.08	0.93
IRIS_313-10725	Indica	Senegal	21.03	0.86
IRIS_313-10744	Japonica	Indonesia	15.34	0.88
IRIS_313-10793	Tropical_Japonica	Indonesia	19.4	0.9
IRIS_313-10798	Tropical_Japonica	Indonesia	17.36	0.9
IRIS_313-10834	Tropical_Japonica	India	15.48	0.89
IRIS_313-10840	Temperate_Japonica	South Korea	16.47	0.92
IRIS_313-10857	Indica	India	26.16	0.87
IRIS_313-10858	Indica	India	33.15	0.89
IRIS_313-10863	Indica	India	26.42	0.87
IRIS_313-10870	Tropical_Japonica	India	22.13	0.91
IRIS_313-10872	Japonica	India	24.86	0.93
IRIS_313-10916	Temperate_Japonica	Cambodia	15.67	0.9
IRIS_313-10918	Japonica	Philippines	26.15	0.92
IRIS_313-10923	Tropical_Japonica	Thailand	16.02	0.88
IRIS_313-10960	Tropical_Japonica	Indonesia	15.86	0.87
IRIS_313-10975	Indica	Bangladesh	25.31	0.87
IRIS_313-10986	Indica	Bangladesh	25.61	0.87
IRIS_313-10994	Japonica	Philippines	16.33	0.87
IRIS_313-10999	Tropical_Japonica	Indonesia	16.05	0.87
IRIS_313-11044	Japonica	Malaysia	21.67	0.91
IRIS_313-11046	Japonica	Malaysia	25.16	0.91
IRIS_313-11083	Indica	Laos	27.82	0.87
IRIS_313-11085	Indica	Laos	24.58	0.87

IRIS_313-11089	Indica	Cambodia	27.17	0.87
IRIS_313-11094	Tropical_Japonica	Laos	22.27	0.91
IRIS_313-11097	Indica	Philippines	27.54	0.88
IRIS_313-11102	Tropical_Japonica	Liberia	29.31	0.92
IRIS_313-11103	Tropical_Japonica	Liberia	24.61	0.92
IRIS_313-11104	Tropical_Japonica	Liberia	20.66	0.91
IRIS_313-11113	Indica	Bangladesh	24.21	0.87
IRIS_313-11118	Indica	Vietnam	25.09	0.87
IRIS_313-11151	Indica	Myanmar	24.4	0.86
IRIS_313-11202	Temperate_Japonica	China	22.88	0.93
IRIS_313-11205	Indica	Bangladesh	23.75	0.85
IRIS_313-11229	Indica	Bangladesh	22.28	0.86
IRIS_313-11234	Indica	Philippines	27.39	0.87
IRIS_313-11238	Tropical_Japonica	Brazil	20.1	0.9
IRIS_313-11239	Indica	Indonesia	28.31	0.88
IRIS_313-11241	Indica	Bangladesh	27.5	0.87
IRIS_313-11242	Indica	India	29.14	0.89
IRIS_313-11244	Indica	India	25.16	0.87
IRIS_313-11245	Indica	India	24.95	0.87
IRIS_313-11247	Indica	India	24.76	0.86
IRIS_313-11251	Indica	Philippines	24.18	0.86
IRIS_313-11253	Indica	Suriname	22.74	0.87
IRIS_313-11256	Indica	India	22.4	0.86
IRIS_313-11260	Indica	India	27.75	0.89
IRIS_313-11263	Indica	India	25.87	0.87
IRIS_313-11266	Indica	India	25.19	0.87
IRIS_313-11267	Indica	India	26.14	0.87
IRIS_313-11273	Indica	India	30.95	0.87
IRIS_313-11279	Indica	India	29.31	0.89
IRIS_313-11395	Indica	Indonesia	22.28	0.86
IRIS_313-11642	Indica	India	24.3	0.87
IRIS_313-11643	Indica	India	34.37	0.88
IRIS_313-11644	Indica	India	28.87	0.88
IRIS_313-11645	Indica	India	34.39	0.88
IRIS_313-11646	Indica	India	35.29	0.88
IRIS_313-11648	Indica	India	26.98	0.87
IRIS_313-11651	Temperate_Japonica	China	33.15	0.96
IRIS_313-11652	Temperate_Japonica	China	26.9	0.93
IRIS_313-11656	Indica	Indonesia	34.74	0.88
IRIS_313-11659	Tropical_Japonica	Sierra Leone	25.83	0.92
IRIS_313-11663	Indica	Zimbabwe	30.14	0.88
IRIS_313-11664	Indica	China	38.39	0.89
IRIS_313-11665	Indica	China	36.32	0.89

IRIS_313-11667	Indica	China	23.62	0.87
IRIS_313-11668	Indica	China	36.08	0.89
IRIS_313-11669	Indica	China	51.09	0.91
IRIS_313-11677	Indica	Thailand	25.01	0.87
IRIS_313-11681	Indica	Thailand	34.8	0.88
IRIS_313-11683	Indica	Thailand	33.38	0.88
IRIS_313-11684	Indica	Thailand	27.42	0.87
IRIS_313-11685	Indica	Thailand	30.53	0.88
IRIS_313-11686	Indica	Thailand	34.35	0.89
IRIS_313-11687	Indica	Thailand	31.7	0.88
IRIS_313-11692	Indica	Taiwan	28.46	0.88
IRIS_313-11700	Indica	Thailand	30.55	0.88
IRIS_313-11704	Indica	Thailand	26.91	0.87
IRIS_313-11705	Indica	Thailand	29.11	0.88
IRIS_313-11707	Indica	Thailand	26.99	0.88
IRIS_313-11708	Indica	Thailand	26.81	0.88
IRIS_313-11709	Indica	Thailand	28.74	0.88
IRIS_313-11710	Indica	Thailand	34.09	0.89
IRIS_313-11716	Indica	Guinea	25.47	0.87
IRIS_313-11717	Indica	Indonesia	34.52	0.88
IRIS_313-11719	Indica	Thailand	28.36	0.88
IRIS_313-11720	Indica	Thailand	25.39	0.87
IRIS_313-11721	Indica	Thailand	27.9	0.88
IRIS_313-11722	Indica	Bangladesh	36.38	0.89
IRIS_313-11723	Indica	Guinea	40.18	0.89
IRIS_313-11727	Indica	China	31.55	0.89
IRIS_313-11728	Indica	China	30.78	0.88
IRIS_313-11730	Indica	China	32.66	0.89
IRIS_313-11731	Indica	China	33.69	0.89
IRIS_313-11732	Indica	China	24.46	0.88
IRIS_313-11733	Indica	China	21.76	0.87
IRIS_313-11734	Indica	China	24.18	0.87
IRIS_313-11740	Indica	Ghana	32.57	0.89
IRIS_313-11744	Indica	China	31	0.89
IRIS_313-11745	Indica	China	31.86	0.89
IRIS_313-11746	Indica	China	28.84	0.88
IRIS_313-11748	Indica	China	27.86	0.88
IRIS_313-11750	Indica	China	26.81	0.88
IRIS_313-11751	Indica	China	23.45	0.87
IRIS_313-11752	Indica	China	31.61	0.88
IRIS_313-11755	Tropical_Japonica	Liberia	32.33	0.93
IRIS_313-11757	Indica	Madagascar	23.71	0.87
IRIS_313-11760	Indica	Madagascar	26.04	0.88

IRIS_313-11761	Indica	Ivory Coast	30.32	0.88
IRIS_313-11762	Indica	Madagascar	27.46	0.88
IRIS_313-11786	Indica	Gambia	31.14	0.87
IRIS_313-11787	Indica	Gambia	21.9	0.85
IRIS_313-11791	Indica	Madagascar	32.25	0.89
IRIS_313-11795	Indica	China	28.3	0.87
IRIS_313-11797	Indica	China	31.86	0.88
IRIS_313-11798	Indica	China	26.52	0.87
IRIS_313-11799	Indica	China	23.45	0.87
IRIS_313-11800	Temperate_Japonica	China	38.45	0.96
IRIS_313-11801	Indica	China	43.1	0.89
IRIS_313-11804	Indica	China	33.06	0.88
IRIS_313-11805	Indica	China	36.96	0.89
IRIS_313-11806	Indica	China	22.04	0.86
IRIS_313-11811	Indica	Kenya	28.06	0.87
IRIS_313-11812	Indica	Kenya	41.86	0.89
IRIS_313-11813	Indica	Kenya	26.96	0.87
IRIS_313-11814	Indica	Kenya	28.41	0.87
IRIS_313-11815	Indica	Kenya	29.2	0.88
IRIS_313-11816	Indica	Myanmar	21.53	0.86
IRIS_313-11817	Indica	Myanmar	29.24	0.89
IRIS_313-11819	Indica	Myanmar	33.9	0.88
IRIS_313-11820	Indica	Myanmar	26.41	0.87
IRIS_313-11821	Indica	India	35.56	0.88
IRIS_313-11822	Indica	India	25.35	0.87
IRIS_313-11823	Indica	India	29.28	0.88
IRIS_313-11900	Tropical_Japonica	Thailand	23.63	0.92
IRIS_313-11924	Tropical_Japonica	Thailand	16.18	0.9
IRIS_313-11929	Tropical_Japonica	Philippines	20.51	0.92
IRIS_313-11994	Tropical_Japonica	Philippines	18.14	0.9
IRIS_313-12045	Tropical_Japonica	Indonesia	15.33	0.88
IRIS_313-12058	Indica	Cambodia	26.57	0.88
IRIS_313-12060	Temperate_Japonica	China	19.58	0.92
IRIS_313-12061	Temperate_Japonica	China	17.51	0.92
IRIS_313-12108	Tropical_Japonica	Malaysia	15.59	0.89
IRIS_313-12129	Tropical_Japonica	Laos	16.04	0.9
IRIS_313-12164	Tropical_Japonica	Cambodia	17.4	0.9
IRIS_313-12228	Tropical_Japonica	Laos	19.23	0.91
IRIS_313-12281	Tropical_Japonica	Madagascar	15.6	0.89
IRIS_313-12321	Tropical_Japonica	Laos	15.06	0.87
IRIS_313-12323	Tropical_Japonica	Laos	15.94	0.89
IRIS_313-12349	Tropical_Japonica	Laos	19.97	0.91
IRIS_313-12351	Tropical_Japonica	Laos	15.49	0.88

IRIS_313-7876	Tropical_Japonica	Philippines	16.11	0.9
IRIS_313-7883	Tropical_Japonica	Indonesia	20.09	0.91
IRIS_313-7902	Tropical_Japonica	Philippines	21.4	0.91
IRIS_313-7909	Tropical_Japonica	Philippines	17.99	0.9
IRIS_313-7914	Japonica	Ivory Coast	23.98	0.91
IRIS_313-7924	Japonica	Bolivia	15.94	0.88
IRIS_313-7933	Tropical_Japonica	Nepal	16.95	0.88
IRIS_313-7994	Japonica	Madagascar	16.68	0.9
IRIS_313-8010	Tropical_Japonica	Philippines	18.43	0.9
IRIS_313-8074	Temperate_Japonica	Australia	15.15	0.89
IRIS_313-8085	Temperate_Japonica	Spain	15.19	0.91
IRIS_313-8118	Temperate_Japonica	Portugal	15.42	0.91
IRIS_313-8119	Temperate_Japonica	Bulgaria	16.99	0.93
IRIS_313-8123	Temperate_Japonica	Portugal	15.35	0.9
IRIS_313-8127	Temperate_Japonica	Bulgaria	16.01	0.92
IRIS_313-8151	Temperate_Japonica	Portugal	19.45	0.93
IRIS_313-8173	Temperate_Japonica	United States	20.32	0.9
IRIS_313-8177	Temperate_Japonica	Italy	15.13	0.91
IRIS_313-8305	Indica	India	25.68	0.87
IRIS_313-8312	Indica	Malaysia	25.78	0.87
IRIS_313-8323	Japonica	United States	17.12	0.91
IRIS_313-8356	Temperate_Japonica	Philippines	17.14	0.89
IRIS_313-8381	Tropical_Japonica	Malaysia	15.17	0.9
IRIS_313-8391	Indica	Burkina Fasso	32.53	0.88
IRIS_313-8407	Indica	Malaysia	22.61	0.86
IRIS_313-8436	Tropical_Japonica	Indonesia	17.43	0.9
IRIS_313-8493	Indica	Indonesia	33.37	0.88
IRIS_313-8606	Indica	-	21.2	0.86
IRIS_313-8627	Temperate_Japonica	United States	15.08	0.9
IRIS_313-8703	Indica	Bangladesh	27.53	0.87
IRIS_313-8768	Tropical_Japonica	Ivory Coast	17.27	0.9
IRIS_313-8925	Indica	Sri Lanka	25.15	0.87
IRIS_313-8930	Indica	Bangladesh	22.89	0.86
IRIS_313-8948	Indica	Philippines	28.36	0.88
IRIS_313-9020	Indica	Thailand	27.96	0.87
IRIS_313-9023	Indica	India	25.66	0.87
IRIS_313-9066	Indica	Bangladesh	33.64	0.9
IRIS_313-9148	Indica	Bangladesh	26.98	0.87
IRIS_313-9262	Indica	Bangladesh	24.49	0.86
IRIS_313-9294	Indica	Gambia	29.43	0.88
IRIS_313-9379	Temperate_Japonica	South Korea	18.33	0.93
IRIS_313-9409	Indica	Malaysia	22.38	0.87
IRIS_313-9470	Tropical_Japonica	Indonesia	18.78	0.91

IRIS_313-9590	Indica	Indonesia	22.1	0.87
IRIS_313-9790	Temperate_Japonica	Uruguay	15.96	0.92
List of the 34 removed cultivars after PCA analysis				
IRIS_313-10114	Indica	Burundi	27.45	0.88
IRIS_313-11467	Temperate_Japonica	Philippines	16.62	0.84
IRIS_313-11493	Tropical_Japonica	India	23.23	0.86
IRIS_313-11622	Tropical_Japonica	China	15.71	0.84
IRIS_313-11657	Tropical_Japonica	Nigeria	33.93	0.88
IRIS_313-11671	Temperate_Japonica	Nepal	42.84	0.89
IRIS_313-11673	Indica	Philippines	33.32	0.93
IRIS_313-11674	Tropical_Japonica	Thailand	27.67	0.88
IRIS_313-11691	Indica	Bhutan	31.45	0.93
IRIS_313-11706	Temperate_Japonica	Thailand	17.16	0.85
IRIS_313-11724	Temperate_Japonica	Guinea	24.29	0.87
IRIS_313-11725	Indica	Japan	32.64	0.97
IRIS_313-11737	Indica	India	30.84	0.89
IRIS_313-11738	Tropical_Japonica	India	28.5	0.88
IRIS_313-11739	Indica	Ghana	22.37	0.92
IRIS_313-11747	Temperate_Japonica	China	26.56	0.88
IRIS_313-11754	Tropical_Japonica	Madagascar	32.66	0.91
IRIS_313-11758	Tropical_Japonica	Ivory Coast	26.23	0.88
IRIS_313-11759	Indica	Ivory Coast	32.78	0.93
IRIS_313-11789	Japonica	Madagascar	31.67	0.88
IRIS_313-11790	Indica	Madagascar	30.49	0.91
IRIS_313-11794	Tropical_Japonica	Madagascar	35.43	0.88
IRIS_313-11796	Tropical_Japonica	China	40.97	0.89
IRIS_313-11802	Temperate_Japonica	China	47.52	0.9
IRIS_313-11809	Indica	Kenya	28.36	0.88
IRIS_313-11810	Tropical_Japonica	Kenya	30.31	0.88
IRIS_313-11833	Tropical_Japonica	Thailand	15.74	0.83
IRIS_313-7646	Tropical_Japonica	Madagascar	18.6	0.88
IRIS_313-7719	Temperate_Japonica	Mali	16.46	0.84
IRIS_313-7725	Temperate_Japonica	Madagascar	16.94	0.85
IRIS_313-7911	Tropical_Japonica	Philippines	17.34	0.84
IRIS_313-8864	Indica	Bangladesh	23.67	0.86
IRIS_313-8911	Tropical_Japonica	Thailand	23.95	0.91
IRIS_313-9160	Temperate_Japonica	Senegal	21.46	0.86

Supplementary Table S2 PCA value for 296 selected samples.

Sub_Group	Sample	PCA1	PCA2
Temperate_Japonica	B001	0.0553578	-0.0837806
Temperate_Japonica	B003	0.0592979	0.0350484
Temperate_Japonica	B005	0.0750891	-0.0975059
Temperate_Japonica	B008	0.0749498	-0.101776
Indica	B012	-0.0431574	0.000278129
Indica	B015	-0.0545468	-0.0173855
Temperate_Japonica	B016	0.070508	-0.0361586
Temperate_Japonica	B017	0.0699643	-0.0886601
Tropical_Japonica	B018	0.0504925	0.114348
Temperate_Japonica	B023	0.055117	-0.0812428
Indica	B026	-0.049645	-0.0238813
Temperate_Japonica	B034	0.0738901	-0.0839482
Temperate_Japonica	B036	0.0578947	0.0713944
Tropical_Japonica	B043	0.0552332	0.115686
Temperate_Japonica	B045	0.074704	-0.102007
Temperate_Japonica	B046	0.0747984	-0.0952057
Temperate_Japonica	B066	0.0708003	-0.0881505
Temperate_Japonica	B070	0.0591536	0.0562264
Indica	B097	-0.0497525	-0.0185254
Temperate_Japonica	B100	0.0709517	-0.0824973
Temperate_Japonica	B111	0.0652985	-0.0760201
Japonica	B143	0.0255563	-0.0955038
Temperate_Japonica	B160	0.0753173	-0.0978473
Temperate_Japonica	B162	0.0751332	-0.10188
Temperate_Japonica	B167	0.074432	-0.104502
Temperate_Japonica	B182	0.0748908	-0.0921166
Temperate_Japonica	B183	0.0738378	-0.094511
Tropical_Japonica	B190	0.0509228	0.111715
Tropical_Japonica	B191	0.0482655	0.083739
Japonica	B196	0.0560336	0.0887642
Temperate_Japonica	B204	0.0735831	-0.080097
Indica	B210	-0.0479426	5.96E-05
Temperate_Japonica	B212	0.0457753	-0.100692
Indica	B214	-0.0542891	-0.00868509
Temperate_Japonica	B218	0.0733802	-0.0931084
Temperate_Japonica	B225	0.0688635	-0.0794412
Temperate_Japonica	B226	0.0747116	-0.0884803
Temperate_Japonica	B230	0.0650246	-0.0412826
Tropical_Japonica	B241	0.0592478	0.0048033
Temperate_Japonica	B250	0.0742912	-0.0973654

Temperate_Japonica	B258	0.0707044	-0.0490536
Indica	B261	-0.0504085	-0.0105025
Indica	B264	-0.0527654	-0.0209963
Indica	B265	-0.053564	-0.00983598
Tropical_Japonica	B266	0.0574943	-0.0128502
Temperate_Japonica	B269	0.075096	-0.0961585
Indica	CX101	-0.052692	-0.00968679
Indica	CX102	-0.0585018	-0.00688339
Tropical_Japonica	CX106	0.0518399	0.109328
Japonica	CX109	0.039157	0.0440516
Tropical_Japonica	CX111	0.0565908	0.113508
Japonica	CX138	0.0712546	-0.0852525
Japonica	CX139	0.0393394	0.0419352
Temperate_Japonica	CX142	0.0623598	0.016713
Indica	CX147	-0.0571931	-0.0101755
Indica	CX150	-0.0615407	-0.0142942
Indica	CX152	-0.0589525	-0.0185201
Indica	CX154	-0.0549844	-0.0119224
Indica	CX155	-0.0572223	-0.00175081
Temperate_Japonica	CX16	0.0712759	-0.104451
Japonica	CX205	0.0539653	0.0368337
Tropical_Japonica	CX220	0.0592047	0.112758
Indica	CX225	-0.0544563	-0.00642995
Japonica	CX241	0.0545365	0.106818
Japonica	CX243	0.0293221	0.084679
Japonica	CX262	0.0517174	0.10262
Tropical_Japonica	CX269	0.0549607	0.109422
Japonica	CX285	0.0550781	0.091974
Indica	CX3	-0.0579651	-0.00944154
Temperate_Japonica	CX306	0.0759967	-0.1048
Temperate_Japonica	CX315	0.0737345	-0.102959
Temperate_Japonica	CX329	0.0754838	-0.102174
Japonica	CX353	0.0669716	-0.0340765
Temperate_Japonica	CX354	0.0578832	-0.0406425
Japonica	CX355	0.0213718	0.0799854
Temperate_Japonica	CX383	0.0718264	-0.0714697
Japonica	CX389_Jinyuan85	0.034133	-0.099428
Temperate_Japonica	CX391	0.0679922	-0.0430324
Indica	CX98	-0.0543558	0.00193439
Indica	CX99	-0.0523137	0.00432207
Indica	IRIS_313-10010	-0.0566857	0.00225119
Temperate_Japonica	IRIS_313-10097	0.0754002	-0.101133
Indica	IRIS_313-10171	-0.0498145	-0.00506118

Indica	IRIS_313-10177	-0.0517861	-0.00576283
Indica	IRIS_313-10235	-0.0580359	-0.00863341
Temperate_Japonica	IRIS_313-10440	0.0693499	-0.100843
Indica	IRIS_313-10511	-0.0558787	0.0021948
Tropical_Japonica	IRIS_313-10541	0.0530303	0.106566
Temperate_Japonica	IRIS_313-10563	0.0720384	-0.0568305
Temperate_Japonica	IRIS_313-10564	0.0747998	-0.104762
Temperate_Japonica	IRIS_313-10570	0.0747346	-0.0939483
Tropical_Japonica	IRIS_313-10577	0.059167	0.0817225
Tropical_Japonica	IRIS_313-10578	0.0469837	0.111994
Tropical_Japonica	IRIS_313-10582	0.0579513	0.082165
Indica	IRIS_313-10609	-0.0492196	-0.00412033
Temperate_Japonica	IRIS_313-10617	0.0733428	-0.0868989
Temperate_Japonica	IRIS_313-10642	0.0752672	-0.104135
Tropical_Japonica	IRIS_313-10657	0.0543453	0.0033344
Temperate_Japonica	IRIS_313-10677	0.0748161	-0.0949095
Indica	IRIS_313-10725	-0.0576939	0.00201877
Japonica	IRIS_313-10744	0.038653	0.0546729
Tropical_Japonica	IRIS_313-10793	0.039814	0.0850117
Tropical_Japonica	IRIS_313-10798	0.0564092	0.0943033
Tropical_Japonica	IRIS_313-10834	0.0634921	-0.0108094
Temperate_Japonica	IRIS_313-10840	0.0738903	-0.0947345
Indica	IRIS_313-10857	-0.0339276	0.0148662
Indica	IRIS_313-10858	-0.0535259	-0.000338279
Indica	IRIS_313-10863	-0.060035	-0.00666279
Tropical_Japonica	IRIS_313-10870	0.0497054	0.0197686
Japonica	IRIS_313-10872	0.0466302	0.0109735
Temperate_Japonica	IRIS_313-10916	0.0705913	-0.0491109
Japonica	IRIS_313-10918	0.0244849	0.080219
Tropical_Japonica	IRIS_313-10923	0.0563629	-0.00279744
Tropical_Japonica	IRIS_313-10960	0.0465452	0.092643
Indica	IRIS_313-10975	-0.0537634	0.00288543
Indica	IRIS_313-10986	-0.0566879	-0.00228256
Japonica	IRIS_313-10994	0.0444462	0.0984275
Tropical_Japonica	IRIS_313-10999	0.0494433	0.101523
Japonica	IRIS_313-11044	0.0512647	0.0799389
Japonica	IRIS_313-11046	0.047706	0.0905509
Indica	IRIS_313-11083	-0.0574042	0.000838275
Indica	IRIS_313-11085	-0.0525579	-0.00387102
Indica	IRIS_313-11089	-0.0583459	-0.00553949
Tropical_Japonica	IRIS_313-11094	0.0574754	0.00432671
Indica	IRIS_313-11097	-0.0443356	0.0207926
Tropical_Japonica	IRIS_313-11102	0.0532991	0.110689

Tropical_Japonica	IRIS_313-11103	0.0625508	0.107482
Tropical_Japonica	IRIS_313-11104	0.0626754	0.106698
Indica	IRIS_313-11113	-0.0558971	0.000926141
Indica	IRIS_313-11118	-0.0606789	-0.00669655
Indica	IRIS_313-11151	-0.0591317	-0.0155124
Temperate_Japonica	IRIS_313-11202	0.0738187	-0.0970006
Indica	IRIS_313-11205	-0.0578238	-0.00293588
Indica	IRIS_313-11229	-0.057753	-0.00159541
Indica	IRIS_313-11234	-0.0560742	0.000871283
Tropical_Japonica	IRIS_313-11238	0.05046	0.0958324
Indica	IRIS_313-11239	-0.0557351	-0.0107224
Indica	IRIS_313-11241	-0.0574763	-0.0118873
Indica	IRIS_313-11242	-0.0565461	-0.00366094
Indica	IRIS_313-11244	-0.0575098	-0.0104994
Indica	IRIS_313-11245	-0.0588548	-0.00520839
Indica	IRIS_313-11247	-0.0575922	-0.0157561
Indica	IRIS_313-11251	-0.0584455	-0.0233533
Indica	IRIS_313-11253	-0.0479907	-0.000537322
Indica	IRIS_313-11256	-0.057312	-0.00232463
Indica	IRIS_313-11260	-0.0568442	-0.0147387
Indica	IRIS_313-11263	-0.0353847	-0.0267939
Indica	IRIS_313-11266	-0.053478	0.00415034
Indica	IRIS_313-11267	-0.0579543	-0.000702487
Indica	IRIS_313-11273	-0.0611082	-0.00626245
Indica	IRIS_313-11279	-0.0501163	-1.04E-05
Indica	IRIS_313-11395	-0.0586061	-0.00680274
Indica	IRIS_313-11642	-0.0561615	0.00135012
Indica	IRIS_313-11643	-0.0556942	-0.0150073
Indica	IRIS_313-11644	-0.0510652	-0.00212366
Indica	IRIS_313-11645	-0.0561029	0.000853498
Indica	IRIS_313-11646	-0.0577142	0.00428712
Indica	IRIS_313-11648	-0.0593358	0.00257624
Temperate_Japonica	IRIS_313-11651	0.0698197	-0.0442622
Temperate_Japonica	IRIS_313-11652	0.0738707	-0.102249
Indica	IRIS_313-11656	-0.0597251	-0.0206218
Tropical_Japonica	IRIS_313-11659	0.0600665	0.104283
Indica	IRIS_313-11663	-0.0514349	0.00659263
Indica	IRIS_313-11664	-0.0618472	-0.0170455
Indica	IRIS_313-11665	-0.0550648	-0.0125847
Indica	IRIS_313-11667	-0.0599328	-0.0129993
Indica	IRIS_313-11668	-0.0543781	-0.014806
Indica	IRIS_313-11669	-0.0513419	-0.00908779
Indica	IRIS_313-11677	-0.0562759	-0.0190649

Indica	IRIS_313-11681	-0.0603104	-0.00479548
Indica	IRIS_313-11683	-0.0599917	-0.00256723
Indica	IRIS_313-11684	-0.0591439	0.00333599
Indica	IRIS_313-11685	-0.0606937	-0.00833032
Indica	IRIS_313-11686	-0.0598586	-0.00636648
Indica	IRIS_313-11687	-0.0595985	0.00185354
Indica	IRIS_313-11692	-0.0576158	-0.0218039
Indica	IRIS_313-11700	-0.0577359	-0.00455133
Indica	IRIS_313-11704	-0.0602728	-0.0141233
Indica	IRIS_313-11705	-0.0568432	-0.00484979
Indica	IRIS_313-11707	-0.058911	0.00400947
Indica	IRIS_313-11708	-0.0573447	0.00141552
Indica	IRIS_313-11709	-0.0598309	-0.00460157
Indica	IRIS_313-11710	-0.0583017	0.00292871
Indica	IRIS_313-11716	-0.0492343	-0.00696437
Indica	IRIS_313-11717	-0.0621507	-0.0127407
Indica	IRIS_313-11719	-0.0592369	-0.00492915
Indica	IRIS_313-11720	-0.0612913	-0.00373235
Indica	IRIS_313-11721	-0.0595883	-0.00661976
Indica	IRIS_313-11722	-0.0578107	-0.00965476
Indica	IRIS_313-11723	-0.0566344	0.0022187
Indica	IRIS_313-11727	-0.053629	-0.00875838
Indica	IRIS_313-11728	-0.061288	-0.0128793
Indica	IRIS_313-11730	-0.055398	-0.0157837
Indica	IRIS_313-11731	-0.0573609	-0.0125102
Indica	IRIS_313-11732	-0.0553522	-0.0181653
Indica	IRIS_313-11733	-0.0509978	-0.00923126
Indica	IRIS_313-11734	-0.0578781	-0.0111842
Indica	IRIS_313-11740	-0.0589021	-0.00840543
Indica	IRIS_313-11744	-0.0521967	-0.00775826
Indica	IRIS_313-11745	-0.0534124	-0.010481
Indica	IRIS_313-11746	-0.0568462	-0.0161076
Indica	IRIS_313-11748	-0.0543389	-0.00514679
Indica	IRIS_313-11750	-0.0597162	-0.0157793
Indica	IRIS_313-11751	-0.0553875	-0.012217
Indica	IRIS_313-11752	-0.0566537	-0.0219159
Tropical_Japonica	IRIS_313-11755	0.0603581	0.10997
Indica	IRIS_313-11757	-0.056698	-0.0157651
Indica	IRIS_313-11760	-0.0579491	0.00719809
Indica	IRIS_313-11761	-0.0563424	0.00601944
Indica	IRIS_313-11762	-0.0567875	-0.00205294
Indica	IRIS_313-11786	-0.0604147	-0.00403758
Indica	IRIS_313-11787	-0.052267	0.00100423

Indica	IRIS_313-11791	-0.0314547	0.0218892
Indica	IRIS_313-11795	-0.05596	-0.0137011
Indica	IRIS_313-11797	-0.0519455	-0.00799381
Indica	IRIS_313-11798	-0.0547223	-0.00437122
Indica	IRIS_313-11799	-0.053409	-0.009285
Temperate_Japonica	IRIS_313-11800	0.0746104	-0.102219
Indica	IRIS_313-11801	-0.0603157	-0.0222415
Indica	IRIS_313-11804	-0.054683	-0.0249345
Indica	IRIS_313-11805	-0.0549679	-0.0044708
Indica	IRIS_313-11806	-0.0535828	-0.0160361
Indica	IRIS_313-11811	-0.0567326	0.00628478
Indica	IRIS_313-11812	-0.0572521	0.00254312
Indica	IRIS_313-11813	-0.0558333	0.00587243
Indica	IRIS_313-11814	-0.0582361	-0.000435592
Indica	IRIS_313-11815	-0.057031	0.00411998
Indica	IRIS_313-11816	-0.0572215	-0.00643219
Indica	IRIS_313-11817	-0.0555537	-0.00678458
Indica	IRIS_313-11819	-0.0641193	-0.0140953
Indica	IRIS_313-11820	-0.058612	-0.00742026
Indica	IRIS_313-11821	-0.0566843	-0.0018108
Indica	IRIS_313-11822	-0.0556755	-0.00118854
Indica	IRIS_313-11823	-0.0589058	0.00131304
Tropical_Japonica	IRIS_313-11900	0.0595196	-0.0330738
Tropical_Japonica	IRIS_313-11924	0.0651088	-0.0241984
Tropical_Japonica	IRIS_313-11929	0.0541067	0.104344
Tropical_Japonica	IRIS_313-11994	0.04663	0.11671
Tropical_Japonica	IRIS_313-12045	0.0550256	0.103727
Indica	IRIS_313-12058	-0.0592107	-7.18E-05
Temperate_Japonica	IRIS_313-12060	0.07373	-0.0806585
Temperate_Japonica	IRIS_313-12061	0.0751204	-0.0918919
Tropical_Japonica	IRIS_313-12108	0.0504326	0.101653
Tropical_Japonica	IRIS_313-12129	0.0559572	0.00157865
Tropical_Japonica	IRIS_313-12164	0.0474951	0.0718551
Tropical_Japonica	IRIS_313-12228	0.0604452	-0.00506658
Tropical_Japonica	IRIS_313-12281	0.0561766	0.100394
Tropical_Japonica	IRIS_313-12321	0.0596933	-0.0089248
Tropical_Japonica	IRIS_313-12323	0.058124	-0.00353759
Tropical_Japonica	IRIS_313-12349	0.063295	-0.00453674
Tropical_Japonica	IRIS_313-12351	0.0560225	-0.0170174
Tropical_Japonica	IRIS_313-7876	0.0485999	0.0994248
Tropical_Japonica	IRIS_313-7883	0.0535622	0.079601
Tropical_Japonica	IRIS_313-7902	0.0360053	0.08642
Tropical_Japonica	IRIS_313-7909	0.0245093	0.0765802

Japonica	IRIS_313-7914	0.0567871	0.122256
Japonica	IRIS_313-7924	0.0265791	0.0791213
Tropical_Japonica	IRIS_313-7933	0.0367327	0.0214813
Japonica	IRIS_313-7994	0.0538843	0.105356
Tropical_Japonica	IRIS_313-8010	0.0487738	0.0960284
Temperate_Japonica	IRIS_313-8074	0.0497948	0.100865
Temperate_Japonica	IRIS_313-8085	0.0690618	-0.00920522
Temperate_Japonica	IRIS_313-8118	0.0604798	-0.0689123
Temperate_Japonica	IRIS_313-8119	0.0674865	-0.0485043
Temperate_Japonica	IRIS_313-8123	0.0658046	-0.0282324
Temperate_Japonica	IRIS_313-8127	0.0694884	-0.0491367
Temperate_Japonica	IRIS_313-8151	0.0664929	-0.0219813
Temperate_Japonica	IRIS_313-8173	0.029693	0.0637315
Temperate_Japonica	IRIS_313-8177	0.0567926	0.0484628
Indica	IRIS_313-8305	-0.0562662	0.0012962
Indica	IRIS_313-8312	-0.054437	0.00452076
Japonica	IRIS_313-8323	0.0538997	0.111495
Temperate_Japonica	IRIS_313-8356	0.0594771	0.0960561
Tropical_Japonica	IRIS_313-8381	0.0581262	0.0976409
Indica	IRIS_313-8391	-0.0543319	0.0015152
Indica	IRIS_313-8407	-0.0586781	-0.000340163
Tropical_Japonica	IRIS_313-8436	0.0429839	0.0665321
Indica	IRIS_313-8493	-0.0601908	-0.00734127
Indica	IRIS_313-8606	-0.0550944	0.00249484
Temperate_Japonica	IRIS_313-8627	0.0560226	0.0927706
Indica	IRIS_313-8703	-0.0572143	-0.00371984
Tropical_Japonica	IRIS_313-8768	0.0498886	0.106088
Indica	IRIS_313-8925	-0.0540726	-8.05E-05
Indica	IRIS_313-8930	-0.0603432	-0.0096649
Indica	IRIS_313-8948	-0.0572042	-0.00518127
Indica	IRIS_313-9020	-0.0597627	-0.0019242
Indica	IRIS_313-9023	-0.0536344	-0.020832
Indica	IRIS_313-9066	-0.0341119	0.0277044
Indica	IRIS_313-9148	-0.0573562	-0.00666078
Indica	IRIS_313-9262	-0.0585375	0.00372362
Indica	IRIS_313-9294	-0.0541136	0.00224468
Temperate_Japonica	IRIS_313-9379	0.0728289	-0.0737546
Indica	IRIS_313-9409	-0.0574398	0.00187411
Tropical_Japonica	IRIS_313-9470	0.0526664	0.0873372
Indica	IRIS_313-9590	-0.0566248	0.00422653
Temperate_Japonica	IRIS_313-9790	0.0573546	0.0372686

Supplementary Table S3 List of African cultivated rice (*O. glaberrima*) and wild rice (*O. rufipogon* and *O. nivara*) used to generate phylogenetic tree.

Sample ID	Data Source	Species	Origin Place	Depth	Coverage[1]
IRGC88812	Zhang et al.,2014	*O. nivara*	Laos	68.61	*0.99*
MV 89-80	Xu et al.,2011	*O. nivara*	Medinipur, India	18.55	*0.96*
HK 47	Xu et al.,2011	*O. nivara*	Madhya Pradesh, India	15.78	*0.95*
042/87/34	Xu et al.,2011	*O. nivara*	Dhoni, India	19.22	*0.98*
CA 97-053	Xu et al.,2011	*O. nivara*	Sopoir Tep, Cambodia	16.86	*0.97*
L 89-12	Xu et al.,2011	*O. nivara*	Vientiane, Laos	17.41	*0.96*
VOC4	Xu et al.,2011	*O. rufipogon*	Nepal	18.85	*0.98*
Yuan 3-9	Xu et al.,2011	*O. rufipogon*	Yunnan,China	13.57	*0.94*
DAL DHAN	Xu et al.,2011	*O. rufipogon*	Chakaria, Bangladesh	19.21	*0.98*
P46	Xu et al.,2011	*O. rufipogon*	Hainan, China, China	15.32	*0.97*
PADI PADIAN	Xu et al.,2011	*O. rufipogon*	Kromat Watu, Indonesia	17.32	*0.96*
W1943	Xu et al.,2011, Ohyanagi et al.,2016 and Yang et al., 2012	*O. rufipogon*	Jiangxi, China	20.8	*0.97*
W3105	Huang et al.,2012	*O. rufipogon*	India	40	*0.86*
W1559	Huang et al.,2012	*O. rufipogon*	Thailand	4.35	*0.06*
W3072	Huang et al.,2012	*O. rufipogon*	Hainan, China	4.73	*0.16*
W1087	Huang et al.,2012	*O. rufipogon*	India	6.25	0.41
W1093	Huang et al.,2012	*O. rufipogon*	India	5.09	0.14
W1096	Huang et al.,2012	*O. rufipogon*	India	4.84	0.09
W1683	Huang et al.,2012	*O. rufipogon*	India	6.75	0.5
W2022	Huang et al.,2012	*O. rufipogon*	Indonesia	4.27	0.06
W2024	Huang et al.,2012	*O. rufipogon*	Indonesia	4.38	0.09
W0171	Huang et al.,2012	*O. rufipogon*	Thailand	6.07	0.48
W1790	Huang et al.,2012	*O. rufipogon*	Thailand	6.72	0.5
W1849	Huang et al.,2012	*O. rufipogon*	Thailand	9.77	0.87
W1850	Huang et al.,2012	*O. rufipogon*	Thailand	4.57	0.12
W1854	Huang et al.,2012	*O. rufipogon*	Thailand	4.73	0.14
W1859	Huang et al.,2012	*O. rufipogon*	Thailand	4.65	0.16
W1870	Huang et al.,2012	*O. rufipogon*	Thailand	4.18	0.07
W1940	Huang et al.,2012	*O. rufipogon*	Thailand	5.04	0.29
W2282	Huang et al.,2012	*O. rufipogon*	Thailand	4.9	0.03
W1715	Huang et al.,2012	*O. rufipogon*	China	5.04	0.22
W2198	Huang et al.,2012	*O. rufipogon*	China	4.36	0.08
W3046	Huang et al.,2012	*O. rufipogon*	Guangdong, China	5.1	*0.15*
W3048	Huang et al.,2012	*O. rufipogon*	Guangxi, China	4.58	*0.08*
W3065	Huang et al.,2012	*O. rufipogon*	Hainan, China	4.02	*0.08*

W3070	Huang et al.,2012	*O. rufipogon*	Hainan, China	4.16	*0.09*
W1748	Huang et al.,2012	*O. rufipogon*	India	6.41	*0.5*
W1777	Huang et al.,2012	*O. rufipogon*	India	4.84	*0.14*
W1725	Huang et al.,2012	*O. rufipogon*	Thailand	6.92	*0.52*
IRGC101049	Wang et al.2014	*O. glaberrima*	Senegal,Africa	116.74	*0.98*
IRGC103469	Wang et al.2014	*O. glaberrima*	Burkina Faso,Africa	11.85	*0.87*
IRGC103472	Wang et al.2014	*O. glaberrima*	Burkina Faso,Africa	11.92	*0.85*
IRGC103520	Wang et al.2014	*O. glaberrima*	Mali,Africa	16.04	*0.88*
IRGC103632	Wang et al.2014	*O. glaberrima*	Mali,Africa	11.69	*0.84*
IRGC103937	Wang et al.2014	*O. glaberrima*	Liberia,Africa	11.15	*0.85*
IRGC104206	Wang et al.2014	*O. glaberrima*	Ghana,Africa	11.37	*0.84*
IRGC104574	Wang et al.2014	*O. glaberrima*	Mali,Africa	8.37	*0.78*
IRGC104955	Wang et al.2014	*O. glaberrima*	Sierra Leone,Africa	7.01	*0.75*
IRGC67563	Wang et al.2014	*O. glaberrima*	Ghana,Africa	117.87	*0.98*
IRGC68939	Wang et al.2014	*O. glaberrima*	Madagascar,Africa	118.03	*0.98*
IRGC68976	Wang et al.2014	*O. glaberrima*	Guyana,Africa	113.12	*0.98*
IRGC75500	Wang et al.2014	*O. glaberrima*	Burkina Faso,Africa	110.92	*0.98*
IRGC96841	Wang et al.2014	*O. glaberrima*	Zimbabwe,Africa	120.34	*0.98*
TOG5457	Wang et al.2014	*O. glaberrima*	Nigeria,Africa	9.38	*0.83*
TOG5467	Wang et al.2014	*O. glaberrima*	Nigeria,Africa	13.86	*0.89*
TOG5923	Wang et al.2014	*O. glaberrima*	Liberia,Africa	5.93	*0.64*
TOG5949	Wang et al.2014	*O. glaberrima*	Liberia,Africa	7.58	*0.77*
TOG7025	Wang et al.2014	*O. glaberrima*	Sierra Leone,Africa	11.58	*0.86*
TOG7102	Wang et al.2014	*O. glaberrima*	Mali,Africa	6.07	*0.62*

1. The percentage of the genome regions that covered by over 5 reads.

Huang X, Kurata N, Wei X, Wang ZX, Wang A, Zhao Q, Zhao Y, Liu K, Lu H, Li W, et al. 2012. A map of rice genome variation reveals the origin of cultivated rice. Nature **490**(7421): 497-501.

Ohyanagi, H., Ebata, T., Huang, X., Gong, H., Fujita, M., Mochizuki, T., Toyoda, A., Fujiyama, A., Kaminuma, E., Nakamura, Y., et al. 2016. OryzaGenome: Genome Diversity Database of Wild Oryza Species. Plant Cell Physiology 57 (1):e1.

Wang M, Yu Y, Haberer G, Marri PR, Fan C, Goicoechea JL, Zuccolo A, Song X, Kudrna D, Ammiraju JS, et al. 2014. The genome sequence of African rice *(Oryza glaberrima*) and evidence for independent domestication. Nat Genet **46**(9): 982-988.

Xu X, Liu X, Ge S, Jensen JD, Hu F, Li X, Dong Y, Gutenkunst RN, Fang L, Huang L, et al. 2012. Resequencing 50 accessions of cultivated and wild rice yields markers for identifying agronomically important genes. *Nature Biotechnology* **30**(1): 105-111

Yang CC, Kawahara Y, Mizuno H, Wu J, Matsumoto T, Itoh T. 2012. Independent domestication of Asian rice followed by gene flow from *japonica* to *indica*. Mol Biol Evol **29**(5): 1471-1479.

Zhang QJ, Zhu T, Xia EH, Shi C, Liu YL, Zhang Y, Liu Y, Jiang WK, Zhao YJ, Mao SY, et al. 2014. Rapid diversification of five *Oryza* AA genomes associated with rice adaptation. Proc Natl Acad Sci U S A **111**(46): E4954-4962.

Table S4 List of wild rice obtained from Huang et al. used in this project

Sample	Species	Clade[1]	Original producing area	Depth
W0610	O. *rufipogon*	Or-I	Burma	0.71
W0626	O. *rufipogon*	Or-I	Burma	2.03
W0627	O. *rufipogon*	Or-I	Burma	1.29
W0630	O. *rufipogon*	Or-I	Burma	0.81
W0631	O. *rufipogon*	Or-I	Burma	0.52
W0632	O. *rufipogon*	Or-I	Burma	1.28
W0633	O. *rufipogon*	Or-I	Burma	0.5
W0638	O. *rufipogon*	Or-I	Burma	1.25
W0639	O. *rufipogon*	Or-I	Burma	0.67
W1295	O. *rufipogon*	Or-I	Cambodia	1.07
W2263	O. *rufipogon*	Or-I	Cambodia	1.8
W2296	O. *rufipogon*	Or-I	Cambodia	1.43
W0101	O. *rufipogon*	Or-I	India	1.42
W0102	O. *rufipogon*	Or-I	India	1.4
W0103	O. *rufipogon*	Or-I	India	1.18
W0106	O. *rufipogon*	Or-I	India	1.81
W0107	O. *rufipogon*	Or-I	India	1.93
W0121	O. *rufipogon*	Or-I	India	1.11
W0123	O. *rufipogon*	Or-I	India	0.26
W0124	O. *rufipogon*	Or-I	India	1.07
W0128	O. *rufipogon*	Or-I	India	2.41
W0130	O. *rufipogon*	Or-I	India	1.07
W0147	O. *rufipogon*	Or-I	India	0.21
W0148	O. *rufipogon*	Or-I	India	1.55
W0151	O. *rufipogon*	Or-I	India	1.94
W0152	O. *rufipogon*	Or-I	India	1.73
W1080	O. *rufipogon*	Or-I	India	1.54
W1082	O. *rufipogon*	Or-I	India	1.42
W1083	O. *rufipogon*	Or-I	India	1.37
W1084	O. *rufipogon*	Or-I	India	1.6
W1086	O. *rufipogon*	Or-I	India	1.49
W1090	O. *rufipogon*	Or-I	India	0.26
W1092	O. *rufipogon*	Or-I	India	1.25
W1105	O. *rufipogon*	Or-I	India	1.5
W1107	O. *rufipogon*	Or-I	India	1.59
W1111	O. *rufipogon*	Or-I	India	1.82
W1112	O. *rufipogon*	Or-I	India	0.76
W1117	O. *rufipogon*	Or-I	India	1.58
W1121	O. *rufipogon*	Or-I	India	1.17

W1142	O. *rufipogon*	Or-I	India	1.05
W1143	O. *rufipogon*	Or-I	India	0.76
W1532	O. *rufipogon*	Or-I	India	0.92
W1533	O. *rufipogon*	Or-I	India	0.89
W1666	O. *rufipogon*	Or-I	India	0.68
W1675	O. *rufipogon*	Or-I	India	1.14
W1676	O. *rufipogon*	Or-I	India	1.12
W1677	O. *rufipogon*	Or-I	India	2.61
W1679	O. *rufipogon*	Or-I	India	1.36
W1681	O. *rufipogon*	Or-I	India	0.9
W1685	O. *rufipogon*	Or-I	India	1.51
W1731	O. *rufipogon*	Or-I	India	1.51
W1735	O. *rufipogon*	Or-I	India	1.71
W1737	O. *rufipogon*	Or-I	India	1.14
W1738	O. *rufipogon*	Or-I	India	1.91
W1740	O. *rufipogon*	Or-I	India	2.02
W1741	O. *rufipogon*	Or-I	India	1.33
W1743	O. *rufipogon*	Or-I	India	1.09
W1747	O. *rufipogon*	Or-I	India	1.34
W1749	O. *rufipogon*	Or-I	India	1.33
W1750	O. *rufipogon*	Or-I	India	1.96
W1751	O. *rufipogon*	Or-I	India	0.72
W1753	O. *rufipogon*	Or-I	India	1.46
W1754	O. *rufipogon*	Or-I	India	2.11
W1756	O. *rufipogon*	Or-I	India	1.08
W1757	O. *rufipogon*	Or-I	India	3.79
W1761	O. *rufipogon*	Or-I	India	1.22
W1762	O. *rufipogon*	Or-I	India	0.88
W1770	O. *rufipogon*	Or-I	India	1.11
W1983	O. *rufipogon*	Or-I	India	2.04
W2193	O. *rufipogon*	Or-I	India	1.54
W3105	O. *rufipogon*	Or-I	India	40
W1970	O. *rufipogon*	Or-I	Indonesia	1.53
W2265	O. *rufipogon*	Or-I	Laos	3.58
W2298	O. *rufipogon*	Or-I	Laos	0.89
W2299	O. *rufipogon*	Or-I	Laos	1.45
W2301	O. *rufipogon*	Or-I	Laos	1.96
W2302	O. *rufipogon*	Or-I	Laos	2.55
W2303	O. *rufipogon*	Or-I	Laos	1.5
W2304	O. *rufipogon*	Or-I	Laos	2.66
W2305	O. *rufipogon*	Or-I	Laos	1.64
W2306	O. *rufipogon*	Or-I	Laos	3.15
W2307	O. *rufipogon*	Or-I	Laos	2.53

W0574	O. *rufipogon*	Or-I	Malaya	2.38
W0589	O. *rufipogon*	Or-I	Malaya	1.21
W0590	O. *rufipogon*	Or-I	Malaya	1.94
W0605	O. *rufipogon*	Or-I	Malaya	0.9
W0168	O. *rufipogon*	Or-I	Thailand	1.57
W0170	O. *rufipogon*	Or-I	Thailand	1.84
W0173	O. *rufipogon*	Or-I	Thailand	1.92
W0176	O. *rufipogon*	Or-I	Thailand	1.39
W0178	O. *rufipogon*	Or-I	Thailand	1.06
W0179	O. *rufipogon*	Or-I	Thailand	1.08
W1546	O. *rufipogon*	Or-I	Thailand	1.09
W1547	O. *rufipogon*	Or-I	Thailand	1.67
W1551	O. *rufipogon*	Or-I	Thailand	1.53
W1559	O. *rufipogon*	Or-I	Thailand	4.35
W1619	O. *rufipogon*	Or-I	Thailand	0.99
W1690	O. *rufipogon*	Or-I	Thailand	1.42
W1695	O. *rufipogon*	Or-I	Thailand	1.16
W1696	O. *rufipogon*	Or-I	Thailand	0.67
W1698	O. *rufipogon*	Or-I	Thailand	1.63
W1700	O. *rufipogon*	Or-I	Thailand	1.08
W1726	O. *rufipogon*	Or-I	Thailand	1.31
W1727	O. *rufipogon*	Or-I	Thailand	1.44
W1787	O. *rufipogon*	Or-I	Thailand	1.76
W1788	O. *rufipogon*	Or-I	Thailand	2.95
W1792	O. *rufipogon*	Or-I	Thailand	2.71
W1794	O. *rufipogon*	Or-I	Thailand	1.89
W1795	O. *rufipogon*	Or-I	Thailand	2.07
W1832	O. *rufipogon*	Or-I	Thailand	1.56
W1852	O. *rufipogon*	Or-I	Thailand	1.57
W1853	O. *rufipogon*	Or-I	Thailand	1.38
W1865	O. *rufipogon*	Or-I	Thailand	1.32
W1866	O. *rufipogon*	Or-I	Thailand	1.62
W1879	O. *rufipogon*	Or-I	Thailand	1.4
W1881	O. *rufipogon*	Or-I	Thailand	1.35
W1893	O. *rufipogon*	Or-I	Thailand	1.59
W1912	O. *rufipogon*	Or-I	Thailand	1.75
W1914	O. *rufipogon*	Or-I	Thailand	3.12
W1921	O. *rufipogon*	Or-I	Thailand	1.25
W1925	O. *rufipogon*	Or-I	Thailand	1.23
W1928	O. *rufipogon*	Or-I	Thailand	1.29
W1935	O. *rufipogon*	Or-I	Thailand	0.97
W2268	O. *rufipogon*	Or-I	Thailand	1.71
W2269	O. *rufipogon*	Or-I	Thailand	1.18

W2271	O. *rufipogon*	Or-I	Thailand	1.61
W2275	O. *rufipogon*	Or-I	Thailand	0.93
W2277	O. *rufipogon*	Or-I	Thailand	1.28
W2278	O. *rufipogon*	Or-I	Thailand	2.73
W0624	O. *rufipogon*	Or-II	Burma	0.77
W0628	O. *rufipogon*	Or-II	Burma	2.31
W0634	O. *rufipogon*	Or-II	Burma	1.13
W0635	O. *rufipogon*	Or-II	Burma	0.52
W2288	O. *rufipogon*	Or-II	Cambodia	2.8
W3003	O. *rufipogon*	Or-II	Guangdong, China	0.39
W3067	O. *rufipogon*	Or-II	Hainan, China	3.7
W3068	O. *rufipogon*	Or-II	Hainan, China	1.75
W3072	O. *rufipogon*	Or-II	Hainan, China	4.73
W0108	O. *rufipogon*	Or-II	India	1.79
W0120	O. *rufipogon*	Or-II	India	0.97
W0132	O. *rufipogon*	Or-II	India	1.4
W0157	O. *rufipogon*	Or-II	India	1.2
W1087	O. *rufipogon*	Or-II	India	6.25
W1093	O. *rufipogon*	Or-II	India	5.09
W1096	O. *rufipogon*	Or-II	India	4.84
W1122	O. *rufipogon*	Or-II	India	3.48
W1124	O. *rufipogon*	Or-II	India	3.1
W1126	O. *rufipogon*	Or-II	India	2.85
W1683	O. *rufipogon*	Or-II	India	6.75
W1687	O. *rufipogon*	Or-II	India	2.31
W1736	O. *rufipogon*	Or-II	India	1.63
W1742	O. *rufipogon*	Or-II	India	1.61
W1780	O. *rufipogon*	Or-II	India	2.32
W1292	O. *rufipogon*	Or-II	Indonesia	0.98
W1971	O. *rufipogon*	Or-II	Indonesia	1.88
W1972	O. *rufipogon*	Or-II	Indonesia	2.71
W1974	O. *rufipogon*	Or-II	Indonesia	2.16
W1975	O. *rufipogon*	Or-II	Indonesia	2.74
W1976	O. *rufipogon*	Or-II	Indonesia	1.83
W1977	O. *rufipogon*	Or-II	Indonesia	3.98
W1978	O. *rufipogon*	Or-II	Indonesia	1.99
W1979	O. *rufipogon*	Or-II	Indonesia	2.27
W1981	O. *rufipogon*	Or-II	Indonesia	1.36
W2017	O. *rufipogon*	Or-II	Indonesia	2.62
W2021	O. *rufipogon*	Or-II	Indonesia	2.64
W2022	O. *rufipogon*	Or-II	Indonesia	4.27
W2024	O. *rufipogon*	Or-II	Indonesia	4.38
W2025	O. *rufipogon*	Or-II	Indonesia	1.21

W2030	O. *rufipogon*	Or-II	Indonesia	2.47
W2197	O. *rufipogon*	Or-II	Indonesia	3.63
W2266	O. *rufipogon*	Or-II	Laos	1.47
W2308	O. *rufipogon*	Or-II	Laos	2.47
W2310	O. *rufipogon*	Or-II	Laos	2.28
W2311	O. *rufipogon*	Or-II	Laos	2.35
W0576	O. *rufipogon*	Or-II	Malaya	3.69
W0587	O. *rufipogon*	Or-II	Malaya	1.37
W0594	O. *rufipogon*	Or-II	Malaya	1.37
W0596	O. *rufipogon*	Or-II	Malaya	1.06
W0600	O. *rufipogon*	Or-II	Malaya	0.97
W0606	O. *rufipogon*	Or-II	Malaya	1.18
W0145	O. *rufipogon*	Or-II	Thailand	0.31
W0163	O. *rufipogon*	Or-II	Thailand	3.38
W0164	O. *rufipogon*	Or-II	Thailand	1.58
W0165	O. *rufipogon*	Or-II	Thailand	1.09
W0166	O. *rufipogon*	Or-II	Thailand	0.76
W0169	O. *rufipogon*	Or-II	Thailand	1.12
W0171	O. *rufipogon*	Or-II	Thailand	6.07
W0174	O. *rufipogon*	Or-II	Thailand	3.07
W0175	O. *rufipogon*	Or-II	Thailand	3.68
W0180	O. *rufipogon*	Or-II	Thailand	3.32
W0234	O. *rufipogon*	Or-II	Thailand	2.05
W1550	O. *rufipogon*	Or-II	Thailand	3.92
W1552	O. *rufipogon*	Or-II	Thailand	1.51
W1553	O. *rufipogon*	Or-II	Thailand	1.09
W1554	O. *rufipogon*	Or-II	Thailand	3.1
W1555	O. *rufipogon*	Or-II	Thailand	0.92
W1556	O. *rufipogon*	Or-II	Thailand	1.12
W1557	O. *rufipogon*	Or-II	Thailand	0.7
W1558	O. *rufipogon*	Or-II	Thailand	2.73
W1790	O. *rufipogon*	Or-II	Thailand	6.72
W1798	O. *rufipogon*	Or-II	Thailand	3.28
W1849	O. *rufipogon*	Or-II	Thailand	9.77
W1850	O. *rufipogon*	Or-II	Thailand	4.57
W1854	O. *rufipogon*	Or-II	Thailand	4.73
W1857	O. *rufipogon*	Or-II	Thailand	3.99
W1858	O. *rufipogon*	Or-II	Thailand	1.31
W1859	O. *rufipogon*	Or-II	Thailand	4.65
W1862	O. *rufipogon*	Or-II	Thailand	2.43
W1870	O. *rufipogon*	Or-II	Thailand	4.18
W1873	O. *rufipogon*	Or-II	Thailand	0.78
W1880	O. *rufipogon*	Or-II	Thailand	2.9

W1882	O. *rufipogon*	Or-II	Thailand	2.12
W1884	O. *rufipogon*	Or-II	Thailand	2.3
W1890	O. *rufipogon*	Or-II	Thailand	3.36
W1891	O. *rufipogon*	Or-II	Thailand	1.71
W1895	O. *rufipogon*	Or-II	Thailand	1.95
W1896	O. *rufipogon*	Or-II	Thailand	2.95
W1916	O. *rufipogon*	Or-II	Thailand	3.61
W1919	O. *rufipogon*	Or-II	Thailand	1.72
W1927	O. *rufipogon*	Or-II	Thailand	1.33
W1939	O. *rufipogon*	Or-II	Thailand	1.61
W1940	O. *rufipogon*	Or-II	Thailand	5.04
W2272	O. *rufipogon*	Or-II	Thailand	1.78
W2276	O. *rufipogon*	Or-II	Thailand	1.35
W2282	O. *rufipogon*	Or-II	Thailand	4.9
W2283	O. *rufipogon*	Or-II	Thailand	2.17
W2284	O. *rufipogon*	Or-II	Thailand	1.12
W3091	O. *rufipogon*	Or-II	Yangzi, China	1.81
W3097	O. *rufipogon*	Or-II	Yangzi, China	1.92
W3098	O. *rufipogon*	Or-II	Yangzi, China	1.79
W1715	O. *rufipogon*	Or-II	China	5.04
W0621	O. *rufipogon*	Or-III	Burma	1.39
W0623	O. *rufipogon*	Or-III	Burma	0.5
W0625	O. *rufipogon*	Or-III	Burma	0.5
W0629	O. *rufipogon*	Or-III	Burma	0.36
W0637	O. *rufipogon*	Or-III	Burma	0.95
W2036	O. *rufipogon*	Or-III	Burma	1.38
W2198	O. *rufipogon*	Or-III	China	4.36
W3000	O. *rufipogon*	Or-III	Guangdong, China	0.97
W3001	O. *rufipogon*	Or-III	Guangdong, China	0.69
W3002	O. *rufipogon*	Or-III	Guangdong, China	2.95
W3004	O. *rufipogon*	Or-III	Guangdong, China	2.11
W3005	O. *rufipogon*	Or-III	Guangdong, China	1.19
W3006	O. *rufipogon*	Or-III	Guangdong, China	1.61
W3007	O. *rufipogon*	Or-III	Guangdong, China	2.62
W3008	O. *rufipogon*	Or-III	Guangdong, China	0.68
W3009	O. *rufipogon*	Or-III	Guangdong, China	1.99
W3010	O. *rufipogon*	Or-III	Guangdong, China	2.67
W3011	O. *rufipogon*	Or-III	Guangdong, China	1.27
W3012	O. *rufipogon*	Or-III	Guangdong, China	1.83
W3013	O. *rufipogon*	Or-III	Guangdong, China	1.78
W3014	O. *rufipogon*	Or-III	Guangdong, China	3.6
W3015	O. *rufipogon*	Or-III	Guangdong, China	1.97
W3016	O. *rufipogon*	Or-III	Guangdong, China	0.57

W3017	O. *rufipogon*	Or-III	Guangdong, China	2
W3018	O. *rufipogon*	Or-III	Guangdong, China	1.57
W3019	O. *rufipogon*	Or-III	Guangdong, China	0.83
W3020	O. *rufipogon*	Or-III	Guangdong, China	2.17
W3021	O. *rufipogon*	Or-III	Guangdong, China	1.43
W3022	O. *rufipogon*	Or-III	Guangdong, China	1.58
W3023	O. *rufipogon*	Or-III	Guangdong, China	1.84
W3024	O. *rufipogon*	Or-III	Guangdong, China	0.66
W3025	O. *rufipogon*	Or-III	Guangdong, China	0.53
W3026	O. *rufipogon*	Or-III	Guangdong, China	0.87
W3029	O. *rufipogon*	Or-III	Guangdong, China	0.67
W3030	O. *rufipogon*	Or-III	Guangdong, China	2.41
W3033	O. *rufipogon*	Or-III	Guangdong, China	1.3
W3034	O. *rufipogon*	Or-III	Guangdong, China	2.01
W3035	O. *rufipogon*	Or-III	Guangdong, China	0.94
W3036	O. *rufipogon*	Or-III	Guangdong, China	0.56
W3037	O. *rufipogon*	Or-III	Guangdong, China	1.05
W3038	O. *rufipogon*	Or-III	Guangdong, China	1.23
W3039	O. *rufipogon*	Or-III	Guangdong, China	1
W3040	O. *rufipogon*	Or-III	Guangdong, China	1.75
W3045	O. *rufipogon*	Or-III	Guangdong, China	2.1
W3046	O. *rufipogon*	Or-III	Guangdong, China	5.1
W3027	O. *rufipogon*	Or-III	Guangxi, China	3.94
W3028	O. *rufipogon*	Or-III	Guangxi, China	2.65
W3031	O. *rufipogon*	Or-III	Guangxi, China	1.32
W3032	O. *rufipogon*	Or-III	Guangxi, China	0.73
W3041	O. *rufipogon*	Or-III	Guangxi, China	3.55
W3042	O. *rufipogon*	Or-III	Guangxi, China	1.63
W3043	O. *rufipogon*	Or-III	Guangxi, China	2.55
W3044	O. *rufipogon*	Or-III	Guangxi, China	3.99
W3047	O. *rufipogon*	Or-III	Guangxi, China	3.88
W3048	O. *rufipogon*	Or-III	Guangxi, China	4.58
W3049	O. *rufipogon*	Or-III	Guangxi, China	3.96
W3050	O. *rufipogon*	Or-III	Guangxi, China	3.77
W3051	O. *rufipogon*	Or-III	Guangxi, China	3.23
W3052	O. *rufipogon*	Or-III	Guangxi, China	3.73
W3053	O. *rufipogon*	Or-III	Hainan, China	1.22
W3054	O. *rufipogon*	Or-III	Hainan, China	1.89
W3055	O. *rufipogon*	Or-III	Hainan, China	1.11
W3056	O. *rufipogon*	Or-III	Hainan, China	0.74
W3057	O. *rufipogon*	Or-III	Hainan, China	1.38
W3058	O. *rufipogon*	Or-III	Hainan, China	1.42
W3059	O. *rufipogon*	Or-III	Hainan, China	1.72

W3060	O. *rufipogon*	Or-III	Hainan, China	1.77
W3061	O. *rufipogon*	Or-III	Hainan, China	1.22
W3062	O. *rufipogon*	Or-III	Hainan, China	2.25
W3063	O. *rufipogon*	Or-III	Hainan, China	1.32
W3064	O. *rufipogon*	Or-III	Hainan, China	0.87
W3065	O. *rufipogon*	Or-III	Hainan, China	4.02
W3066	O. *rufipogon*	Or-III	Hainan, China	3.44
W3069	O. *rufipogon*	Or-III	Hainan, China	2.73
W3070	O. *rufipogon*	Or-III	Hainan, China	4.16
W3071	O. *rufipogon*	Or-III	Hainan, China	3.11
W3073	O. *rufipogon*	Or-III	Hainan, China	3.27
W3074	O. *rufipogon*	Or-III	Hainan, China	2.76
W0125	O. *rufipogon*	Or-III	India	2.27
W0126	O. *rufipogon*	Or-III	India	1.62
W0133	O. *rufipogon*	Or-III	India	0.81
W0134	O. *rufipogon*	Or-III	India	1.17
W0135	O. *rufipogon*	Or-III	India	0.85
W0136	O. *rufipogon*	Or-III	India	0.57
W0137	O. *rufipogon*	Or-III	India	0.89
W0138	O. *rufipogon*	Or-III	India	1.02
W0141	O. *rufipogon*	Or-III	India	1.11
W0149	O. *rufipogon*	Or-III	India	1.58
W0153	O. *rufipogon*	Or-III	India	2.54
W0549	O. *rufipogon*	Or-III	India	1.35
W1102	O. *rufipogon*	Or-III	India	0.85
W1114	O. *rufipogon*	Or-III	India	1.2
W1119	O. *rufipogon*	Or-III	India	1.75
W1534	O. *rufipogon*	Or-III	India	0.63
W1668	O. *rufipogon*	Or-III	India	1.36
W1669	O. *rufipogon*	Or-III	India	3.56
W1732	O. *rufipogon*	Or-III	India	1.39
W1739	O. *rufipogon*	Or-III	India	1.3
W1746	O. *rufipogon*	Or-III	India	2.37
W1748	O. *rufipogon*	Or-III	India	6.41
W1759	O. *rufipogon*	Or-III	India	2.8
W1766	O. *rufipogon*	Or-III	India	3.19
W1777	O. *rufipogon*	Or-III	India	4.84
W1782	O. *rufipogon*	Or-III	India	3.48
W1783	O. *rufipogon*	Or-III	India	0.42
W1784	O. *rufipogon*	Or-III	India	0.54
W1989	O. *rufipogon*	Or-III	India	1.25
W1990	O. *rufipogon*	Or-III	India	1.34
W1991	O. *rufipogon*	Or-III	India	1.84

W1993	O. *rufipogon*	Or-III	India	1.33
W1995	O. *rufipogon*	Or-III	India	0.85
W1998	O. *rufipogon*	Or-III	India	2.24
W2003	O. *rufipogon*	Or-III	India	1.88
W2005	O. *rufipogon*	Or-III	India	1.97
W2007	O. *rufipogon*	Or-III	India	1.53
W2008	O. *rufipogon*	Or-III	India	1.64
W2010	O. *rufipogon*	Or-III	India	1.06
W2012	O. *rufipogon*	Or-III	India	1.08
W2014	O. *rufipogon*	Or-III	India	0.88
W1973	O. *rufipogon*	Or-III	Indonesia	2.74
W2267	O. *rufipogon*	Or-III	Laos	2.09
W0573	O. *rufipogon*	Or-III	Malaya	1.86
W0593	O. *rufipogon*	Or-III	Malaya	0.84
W1542	O. *rufipogon*	Or-III	Malaya	1.06
W0172	O. *rufipogon*	Or-III	Thailand	1.13
W1560	O. *rufipogon*	Or-III	Thailand	1.45
W1725	O. *rufipogon*	Or-III	Thailand	6.92
W3075	O. *rufipogon*	Or-III	Yangzi, China	1.53
W3076	O. *rufipogon*	Or-III	Yangzi, China	0.44
W3077	O. *rufipogon*	Or-III	Yangzi, China	0.65
W3078	O. *rufipogon*	Or-III	Yangzi, China	1.05
W3079	O. *rufipogon*	Or-III	Yangzi, China	1.62
W3080	O. *rufipogon*	Or-III	Yangzi, China	1.69
W3081	O. *rufipogon*	Or-III	Yangzi, China	0.93
W3082	O. *rufipogon*	Or-III	Yangzi, China	1.46
W3092	O. *rufipogon*	Or-III	Yangzi, China	2.7
W3093	O. *rufipogon*	Or-III	Yangzi, China	0.27
W3094	O. *rufipogon*	Or-III	Yangzi, China	2.89
W3095	O. *rufipogon*	Or-III	Yangzi, China	1.66
W3096	O. *rufipogon*	Or-III	Yangzi, China	0.86

1. Clades were classified by Huang et al.,2012

Reference:

Huang X, Kurata N, Wei X, Wang ZX, Wang A, Zhao Q, Zhao Y, Liu K, Lu H, Li W, et al. 2012. A map of rice genome variation reveals the origin of cultivated rice. Nature **490**(7421): 497-501.

Table S5 SNP genotype of DR-I. We genotyped all the SNPs within DR-I regions and classified them as Japonica type or Indica type.

Name	Depth	Clade[1]	Original Place	Japonica_Type		Indica_Type		Hete	Unmap
				SNP num	%	SNP num	%		
W1725	5.7	Or-III	Thailand	2582	0.863	410	0.137	439	261
W1114	1.11	Or-III	India	1568	0.859	258	0.141	3	1863
W1560	1.31	Or-III	Thailand	1798	0.849	319	0.151	18	1557
W3093	0.25	Or-III	Yangzi	524	0.849	93	0.151	0	3075
W3096	0.8	Or-III	Yangzi	1442	0.841	272	0.159	31	1947
W3077	0.63	Or-III	Yangzi	1266	0.841	240	0.159	18	2168
W0637	0.79	Or-III	Burma	1153	0.837	224	0.163	12	2303
W3095	1.56	Or-III	Yangzi	2041	0.825	433	0.175	56	1162
P46	14.78	-	Hainan;China	2465	0.824	526	0.176	664	37
W3081	0.83	Or-III	Yangzi	1471	0.824	315	0.176	36	1870
W2198	3.88	Or-III	China	2637	0.818	586	0.182	62	407
W3040	1.49	Or-III	Guangdong	2017	0.817	453	0.183	114	1108
W3015	1.74	Or-III	Guangdong	2038	0.814	467	0.186	190	997
W3092	2.5	Or-III	Yangzi	2346	0.812	542	0.188	166	638
W3037	0.92	Or-III	Guangdong	1534	0.808	364	0.192	61	1733
W3002	2.52	Or-III	Guangdong	2277	0.806	548	0.194	207	660
W3079	1.39	Or-III	Yangzi	1988	0.806	480	0.194	46	1178
W3039	0.91	Or-III	Guangdong	1554	0.805	376	0.195	39	1723
W3078	0.97	Or-III	Yangzi	1669	0.805	405	0.195	8	1610
W1943	20.8	Or-III	Jiangxi	2852	0.800	715	0.200	116	9
W3046	4.36	Or-III	Guangdong	2408	0.796	616	0.204	281	387
W3013	1.6	Or-III	Guangdong	1964	0.789	526	0.211	68	1134
W3076	0.41	Or-III	Yangzi	957	0.788	258	0.212	2	2475
W3005	1.02	Or-III	Guangdong	1469	0.787	398	0.213	47	1778
W3043	2.32	Or-III	Guangxi	2023	0.785	555	0.215	215	899
W3018	1.44	Or-III	Guangdong	1775	0.781	498	0.219	113	1306
W3080	1.58	Or-III	Yangzi	1979	0.780	558	0.220	181	974
W3075	1.46	Or-III	Yangzi	1990	0.779	564	0.221	10	1128
W3026	0.55	Or-III	Guangdong	928	0.776	268	0.224	40	2456
W3038	1.1	Or-III	Guangdong	1706	0.775	495	0.225	21	1470
W3027	3.49	Or-III	Guangxi	2170	0.766	662	0.234	312	548
W3032	0.63	Or-III	Guangxi	1110	0.763	344	0.237	27	2211
W3042	1.48	Or-III	Guangxi	1658	0.762	518	0.238	102	1414
W3045	1.87	Or-III	Guangdong	1771	0.757	569	0.243	178	1174
W3036	0.46	Or-III	Guangdong	860	0.750	287	0.250	19	2526
Yuan3_9	12.83		Yunnan	2676	0.740	938	0.260	23	55
W3048	3.81	Or-III	Guangxi	2322	0.739	821	0.261	80	469

W3024	0.35	Or-III	Guangdong	578	0.730	214	0.270	21	2879
W3074	2.59	Or-III	Hainan	2076	0.727	778	0.273	48	790
W3044	3.38	Or-III	Guangxi	1892	0.727	711	0.273	549	540
W3030	2.27	Or-III	Guangdong	2128	0.726	802	0.274	176	586
W3050	3.56	Or-III	Guangxi	2118	0.719	827	0.281	247	500
W3000	0.89	Or-III	Guangdong	1224	0.707	508	0.293	116	1844
W3047	3.45	Or-III	Guangxi	1942	0.704	818	0.296	417	515
W3028	2.4	Or-III	Guangxi	1699	0.698	735	0.302	351	907
W3029	0.59	Or-III	Guangdong	967	0.695	425	0.305	30	2270
W3052	3.46	Or-III	Guangxi	1891	0.693	839	0.307	386	576
W3009	1.93	Or-III	Guangdong	1581	0.689	714	0.311	341	1056
W3094	2.75	Or-III	Yangzi	1460	0.684	673	0.316	1015	544
W3041	3.18	Or-III	Guangxi	1866	0.682	871	0.318	329	626
W3082	1.36	Or-III	Yangzi	1675	0.680	787	0.320	59	1171
W3071	2.85	Or-III	Hainan	1760	0.666	884	0.334	350	698
W3025	0.3	Or-III	Guangdong	512	0.664	259	0.336	23	2898
W2036	1.18	Or-III	Burma	1336	0.663	680	0.337	136	1540
W3020	1.46	Or-III	Guangdong	1464	0.657	764	0.343	140	1324
W3016	0.52	Or-III	Guangdong	856	0.652	457	0.348	48	2331
W3051	2.6	Or-III	Guangxi	1674	0.651	896	0.349	268	854
W3070	3.82	Or-III	Hainan	1844	0.650	995	0.350	401	452
W3049	3.45	Or-III	Guangxi	1794	0.649	972	0.351	329	597
W3069	2.48	Or-III	Hainan	1689	0.642	943	0.358	270	790
W3006	1.51	Or-III	Guangdong	1314	0.631	767	0.369	224	1387
W3011	1.25	Or-III	Guangdong	1124	0.628	666	0.372	335	1567
W3066	2.97	Or-III	Hainan	1775	0.626	1061	0.374	224	632
W3021	1.36	Or-III	Guangdong	1271	0.621	776	0.379	318	1327
W3058	1.31	Or-III	Hainan	1471	0.619	906	0.381	33	1282
W3059	1.58	Or-III	Hainan	1509	0.616	939	0.384	75	1169
W3061	1.06	Or-III	Hainan	1298	0.613	820	0.387	20	1554
W3031	1.22	Or-III	Guangxi	1292	0.611	823	0.389	186	1391
VOC4	18.52	-	Nepal	1814	0.610	1158	0.390	678	42
W3063	1.2	Or-III	Hainan	1322	0.606	860	0.394	102	1408
W1766	2.79	Or-III	India	1671	0.601	1109	0.399	71	841
W1973	2.33	Or-III	Indonesia	1187	0.598	799	0.402	11	1695
W3062	2.1	Or-III	Hainan	1637	0.594	1118	0.406	163	774
W3008	0.7	Or-III	Guangdong	932	0.592	641	0.408	19	2100
W1759	2.47	Or-III	India	1526	0.592	1050	0.408	175	941
W3073	2.91	Or-III	Hainan	1630	0.591	1126	0.409	244	692
W3057	1.28	Or-III	Hainan	1321	0.579	961	0.421	64	1346
W1746	2.07	Or-III	India	1413	0.573	1053	0.427	130	1096
W3014	3.2	Or-III	Guangdong	1366	0.573	1018	0.427	744	564
W1102	0.72	Or-III	India	810	0.573	604	0.427	9	2269

W3017	1.76	Or-III	Guangdong	1281	0.573	956	0.427	438	1017
W0625	0.49	Or-III	Burma	597	0.567	456	0.433	2	2637
W0623	0.49	Or-III	Burma	572	0.562	446	0.438		2674
W3055	1	Or-III	Hainan	1058	0.561	829	0.439	99	1706
W3012	1.74	Or-III	Guangdong	1437	0.555	1152	0.445	26	1077
W3034	1.78	Or-III	Guangdong	1365	0.554	1099	0.446	345	883
W3033	1.08	Or-III	Guangdong	1067	0.553	864	0.447	117	1644
W3007	2.58	Or-III	Guangdong	1479	0.551	1206	0.449	222	785
W1096	4.16	Or-II	India	1544	0.550	1262	0.450	317	569
W0629	0.34	Or-III	Burma	401	0.547	332	0.453		2959
W3054	1.8	Or-III	Hainan	1341	0.546	1117	0.454	256	978
W3022	1.56	Or-III	Guangdong	1064	0.544	892	0.456	559	1177
W2005	1.61	Or-III	India	1343	0.543	1130	0.457	10	1209
W1534	0.54	Or-III	India	613	0.539	524	0.461	3	2552
W0133	0.69	Or-III	India	744	0.538	639	0.462	4	2305
W1119	1.49	Or-III	India	1145	0.537	988	0.463	11	1548
W1783	0.39	Or-III	India	521	0.537	450	0.463		2721
W3053	1.13	Or-III	Hainan	1088	0.536	941	0.464	147	1516
W0634	0.83	Or-II	Burma	748	0.535	651	0.465	41	2252
W1782	3.01	Or-III	India	1634	0.535	1423	0.465	17	618
W0593	0.75	Or-III	Malaya	753	0.534	656	0.466	4	2279
W2010	0.92	Or-III	India	963	0.534	839	0.466	2	1888
W0621	1.4	Or-III	Burma	1124	0.534	980	0.466	2	1586
W3001	0.63	Or-III	Guangdong	685	0.534	598	0.466	102	2307
W1976	1.58	Or-II	Indonesia	1308	0.532	1149	0.468	45	1190
W0172	0.95	Or-III	Thailand	876	0.532	770	0.468	2	2044
W1989	1.16	Or-III	India	1095	0.531	966	0.469	3	1628
W0136	0.5	Or-III	India	531	0.530	470	0.470	3	2688
W0134	1.04	Or-III	India	892	0.528	796	0.472	4	2000
W0137	0.81	Or-III	India	810	0.528	724	0.472	1	2157
W2003	1.65	Or-III	India	1325	0.527	1189	0.473	10	1168
W1784	0.49	Or-III	India	602	0.526	542	0.474	6	2542
W2014	0.74	Or-III	India	858	0.526	773	0.474	2	2059
W0138	0.89	Or-III	India	841	0.526	758	0.474	3	2090
W0132	1.21	Or-II	India	1017	0.523	928	0.477	34	1713
W1998	1.97	Or-III	India	1452	0.523	1325	0.477	9	906
W0135	0.72	Or-III	India	762	0.523	696	0.477	3	2231
W3065	3.57	Or-III	Hainan	1510	0.522	1383	0.478	269	530
W3064	0.82	Or-III	Hainan	902	0.521	829	0.479	47	1914
W1990	1.32	Or-III	India	1172	0.520	1081	0.480	8	1431
W2008	1.36	Or-III	India	1175	0.519	1087	0.481	3	1427
W1780	1.92	Or-II	India	1379	0.519	1277	0.481	25	1011
W0549	1.19	Or-III	India	1048	0.518	977	0.482	2	1665

W1542	0.94	Or-III	Malaya	894	0.517	835	0.483	2	1961
W0573	1.57	Or-III	Malaya	1176	0.515	1108	0.485	6	1402
W2007	1.28	Or-III	India	1131	0.515	1067	0.485	4	1490
W1739	1.12	Or-III	India	1007	0.514	952	0.486	4	1729
W0120	0.74	Or-II	India	733	0.513	695	0.487	19	2245
W0141	0.99	Or-III	India	839	0.511	802	0.489	4	2047
W1993	1.24	Or-III	India	1147	0.511	1097	0.489	7	1441
W0153	2.29	Or-III	India	1340	0.510	1286	0.490	9	1057
W1991	1.68	Or-III	India	1240	0.510	1192	0.490	8	1252
W3056	0.7	Or-III	Hainan	722	0.509	697	0.491	28	2245
W1995	0.77	Or-III	India	822	0.507	798	0.493	1	2071
W2024	3.74	Or-II	Indonesia	1620	0.506	1581	0.494	40	451
W1971	1.65	Or-II	Indonesia	1233	0.505	1209	0.495	87	1163
W2197	2.85	Or-II	Indonesia	1488	0.504	1465	0.496	159	580
W0624	0.69	Or-II	Burma	664	0.503	656	0.497	3	2369
W1880	2.56	Or-II	Thailand	1465	0.503	1450	0.497	171	606
W2284	0.95	Or-II	Thailand	939	0.502	931	0.498	8	1814
W1979	1.85	Or-II	Indonesia	1313	0.501	1306	0.499	24	1049
W0125	2.03	Or-III	India	1321	0.500	1320	0.500	12	1039
W2012	0.98	Or-III	India	924	0.498	930	0.502	4	1834
W1977	3.46	Or-II	Indonesia	1504	0.497	1521	0.503	160	507
W1857	3.19	Or-II	Thailand	1397	0.492	1443	0.508	184	668
W2017	1.9	Or-II	Indonesia	1227	0.492	1269	0.508	151	1045
W1891	1.43	Or-II	Thailand	1232	0.491	1278	0.509	52	1130
W0600	0.9	Or-II	Malaya	805	0.490	838	0.510	29	2020
W2308	2.23	Or-II	Laos	1318	0.489	1376	0.511	68	930
W1884	2.07	Or-II	Thailand	1356	0.488	1420	0.512	128	788
W2022	3.71	Or-II	Indonesia	1474	0.486	1560	0.514	208	450
W0149	1.43	Or-III	India	1006	0.486	1065	0.514	98	1523
W1890	2.93	Or-II	Thailand	1533	0.486	1624	0.514	26	509
W3010	2.46	Or-III	Guangdong	1023	0.483	1094	0.517	870	705
W1940	4.82	Or-II	Thailand	1621	0.480	1758	0.520	56	257
W1126	2.46	Or-II	India	1334	0.480	1447	0.520	154	757
W1850	4.02	Or-II	Thailand	1450	0.477	1587	0.523	153	502
W0174	2.84	Or-II	Thailand	1382	0.477	1514	0.523	34	762
W1798	3	Or-II	Thailand	1427	0.477	1564	0.523	212	489
W1669	3.45	Or-III	India	1434	0.477	1572	0.523	307	379
PADI_PADIAN	16.48	-	Kromat Watu;Indonesia	1560	0.476	1717	0.524	320	95
W1978	1.7	Or-II	Indonesia	1181	0.476	1301	0.524	61	1149
W0180	2.74	Or-II	Thailand	1335	0.475	1475	0.525	10	872
W1777	4.35	Or-III	India	1432	0.475	1583	0.525	281	396
W1972	2.37	Or-II	Indonesia	1328	0.475	1469	0.525	109	786

W1919	1.61	Or-II	Thailand	1204	0.474	1335	0.526	53	1100
W0108	1.68	Or-II	India	1128	0.474	1252	0.526	20	1292
W3060	1.63	Or-III	Hainan	1146	0.470	1292	0.530	212	1042
W0628	1.92	Or-II	Burma	1161	0.468	1318	0.532	43	1170
W2282	2.99	Or-II	Thailand	1352	0.468	1538	0.532	209	593
W1981	1.2	Or-II	Indonesia	1020	0.467	1162	0.533	3	1507
W1558	2.36	Or-II	Thailand	1299	0.467	1480	0.533	48	865
W1939	1.44	Or-II	Thailand	1121	0.467	1278	0.533	75	1218
W2025	0.97	Or-II	Indonesia	848	0.466	972	0.534	42	1830
W1790	5.59	Or-II	Thailand	1608	0.466	1845	0.534	13	226
W1849	9.13	Or-II	Thailand	1560	0.465	1793	0.535	203	136
W2272	1.48	Or-II	Thailand	1117	0.465	1286	0.535	15	1274
W2021	2.33	Or-II	Indonesia	1337	0.465	1540	0.535	47	768
W2266	1.16	Or-II	Laos	872	0.465	1005	0.535	17	1798
W3091	1.62	Or-II	Yangzi	1082	0.464	1248	0.536	262	1100
W1683	5.63	Or-II	India	1527	0.463	1770	0.537	104	291
W2288	2.7	Or-II	Cambodia	1370	0.463	1592	0.537	128	602
W2283	1.9	Or-II	Thailand	1190	0.462	1384	0.538	126	992
W0171	5.7	Or-II	Thailand	1499	0.459	1770	0.541	173	250
W2030	2.22	Or-II	Indonesia	1284	0.458	1522	0.542	52	834
W3035	0.82	Or-III	Guangdong	737	0.456	878	0.544	199	1878
W3004	1.75	Or-III	Guangdong	944	0.456	1126	0.544	530	1092
W2267	1.77	Or-III	Laos	1234	0.455	1476	0.545	10	972
W1896	2.83	Or-II	Thailand	1351	0.453	1634	0.547	224	483
W1122	2.97	Or-II	India	1367	0.452	1658	0.548	56	611
W1975	2.5	Or-II	Indonesia	1349	0.452	1638	0.548	13	692
W0157	0.89	Or-II	India	656	0.449	804	0.551	25	2207
W1292	0.86	Or-II	Indonesia	703	0.448	865	0.552	35	2089
W1854	4.24	Or-II	Thailand	1457	0.446	1810	0.554	12	413
W1553	1	Or-II	Thailand	763	0.445	950	0.555	37	1942
W3097	1.83	Or-II	Yangzi	1136	0.445	1419	0.555	151	986
W1093	4.47	Or-II	India	1229	0.444	1537	0.556	500	426
W1974	1.98	Or-II	Indonesia	1176	0.444	1473	0.556	42	1001
W3098	1.67	Or-II	Yangzi	1090	0.442	1378	0.558	175	1049
W0576	3.32	Or-II	Malaya	1229	0.441	1556	0.559	294	613
W1552	1.28	Or-II	Thailand	866	0.440	1101	0.560	76	1649
W0169	0.99	Or-II	Thailand	763	0.440	971	0.560	4	1954
W1732	1.24	Or-III	India	846	0.440	1077	0.560	31	1738
W1862	2.18	Or-II	Thailand	1209	0.437	1555	0.563	152	776
W1916	3.31	Or-II	Thailand	1307	0.432	1715	0.568	248	422
W1882	1.96	Or-II	Thailand	1113	0.424	1509	0.576	235	835
W0175	3.47	Or-II	Thailand	1255	0.424	1702	0.576	181	554
W0596	0.97	Or-II	Malaya	727	0.419	1009	0.581	9	1947

W0166	0.53	Or-II	Thailand	407	0.418	566	0.582	1	2718
W1870	3.76	Or-II	Thailand	1334	0.417	1865	0.583	145	348
W1554	2.14	Or-II	Thailand	1062	0.404	1566	0.596	41	1023
W1715		Or-II		1116	0.397	1695	0.603	513	368
W1556	1.01	Or-II	Thailand	726	0.393	1120	0.607	19	1827
W3023	1.47	Or-III	Guangdong	781	0.391	1216	0.609	450	1245
W1859	4.27	Or-II	Thailand	1154	0.377	1906	0.623	212	420
W1983	1.78	Or-I	India	970	0.375	1618	0.625	11	1093
W1756	1.01	Or-I	India	688	0.371	1168	0.629	1	1835
W0606	1.13	Or-II	Malaya	678	0.365	1179	0.635	36	1799
W1087	5.48	Or-II	India	1086	0.364	1901	0.636	319	386
W0165	0.79	Or-II	Thailand	483	0.363	846	0.637		2363
W1748	5.59	Or-III	India	973	0.360	1729	0.640	661	329
DAL_DHAN	17.78	-	Chakaria;Bangladesh	974	0.354	1777	0.646	886	55
W1550	3.29	Or-II	Thailand	883	0.341	1710	0.659	488	611
W3019	0.67	Or-III	Guangdong	460	0.334	917	0.666	110	2205
W0164	1.4	Or-II	Thailand	687	0.334	1372	0.666	55	1578
W0163	3.3	Or-II	Thailand	957	0.327	1969	0.673	237	529
W1895	1.71	Or-II	Thailand	822	0.322	1728	0.678	91	1051
W3068	1.55	Or-II	Hainan	605	0.319	1293	0.681	440	1354
W0126	1.46	Or-III	India	641	0.317	1384	0.683	247	1420
W0594	1.21	Or-II	Malaya	596	0.311	1322	0.689	97	1677
W1740	1.87	Or-I	India	803	0.300	1878	0.700	4	1007
W0151	1.7	Or-I	India	685	0.295	1634	0.705	7	1366
W1743	1.06	Or-I	India	556	0.289	1365	0.711	1	1770
W0234	1.78	Or-II	Thailand	705	0.288	1744	0.712	11	1232
W1858	1.14	Or-II	Thailand	581	0.286	1451	0.714	37	1623
W1927	1.17	Or-II	Thailand	573	0.278	1489	0.722	109	1521
W0639	0.61	Or-I	Burma	337	0.274	892	0.726	6	2457
W1080	1.04	Or-I	India	501	0.274	1329	0.726	61	1801
W1753	1.28	Or-I	India	607	0.271	1630	0.729	4	1451
W1741	1.19	Or-I	India	569	0.267	1562	0.733	1	1560
W2277	1.13	Or-I	Thailand	554	0.266	1531	0.734	1	1606
HK47	15.54	-	Madhya Pradesh;India	951	0.265	2631	0.735	35	75
W0170	1.6	Or-I	Thailand	554	0.257	1604	0.743	8	1526
W2263	1.47	Or-I	Cambodia	525	0.254	1544	0.746	5	1618
W1762	0.75	Or-I	India	390	0.253	1151	0.747		2151
W1687	1.95	Or-II	India	529	0.252	1570	0.748	331	1262
W0106	1.77	Or-I	India	621	0.252	1846	0.748	7	1218
W1866	1.39	Or-I	Thailand	598	0.252	1778	0.748	14	1302
W1832	1.32	Or-I	Thailand	594	0.251	1769	0.749	4	1325
W1736	1.55	Or-II	India	556	0.251	1657	0.749	12	1467

W1698	1.48	Or-I	Thailand	535	0.251	1599	0.749	2	1556
W0633	0.42	Or-I	Burma	216	0.251	646	0.749	3	2827
W1533	0.79	Or-I	India	380	0.250	1138	0.750	1	2173
W1546	0.93	Or-I	Thailand	428	0.249	1291	0.751	1	1972
W2265	2.51	Or-I	Laos	742	0.249	2239	0.751	4	707
W1551	1.38	Or-I	Thailand	516	0.249	1559	0.751	10	1607
W2303	1.28	Or-I	Laos	567	0.248	1715	0.752	3	1407
W0121	0.88	Or-I	India	332	0.245	1024	0.755	155	2181
W1853	1.19	Or-I	Thailand	522	0.244	1614	0.756	1	1555
W1747	1.2	Or-I	India	528	0.244	1633	0.756	2	1529
W1738	1.75	Or-I	India	568	0.244	1760	0.756	7	1357
W1865	1.1	Or-I	Thailand	495	0.242	1547	0.758	9	1641
W0107	1.85	Or-I	India	604	0.242	1891	0.758	7	1190
W2296	1.29	Or-I	Cambodia	522	0.242	1638	0.758	7	1525
W2310	2.03	Or-II	Laos	667	0.242	2094	0.758	44	887
W1555	0.8	Or-II	Thailand	376	0.241	1182	0.759	10	2124
W1685	1.17	Or-I	India	434	0.240	1372	0.760	3	1883
W1727	1.22	Or-I	Thailand	470	0.240	1487	0.760	4	1731
W1749	1.25	Or-I	India	520	0.239	1653	0.761		1519
W1619	0.9	Or-I	Thailand	387	0.239	1231	0.761		2074
W1912	1.48	Or-I	Thailand	552	0.239	1760	0.761	8	1372
W1726	1.18	Or-I	Thailand	453	0.238	1447	0.762	3	1789
W0630	0.73	Or-I	Burma	313	0.237	1008	0.763		2371
W0168	1.41	Or-I	Thailand	509	0.236	1644	0.764	4	1535
W2275	0.79	Or-I	Thailand	378	0.233	1243	0.767	2	2069
W2304	2.38	Or-I	Laos	696	0.233	2291	0.767	8	697
W0574	1.95	Or-I	Malaya	611	0.232	2017	0.768	9	1055
W3067	3.4	Or-II	Hainan	489	0.232	1617	0.768	1028	558
W1547	1.48	Or-I	Thailand	503	0.231	1670	0.769	4	1515
W3105	8.36	Or-I	India	827	0.231	2757	0.769	14	94
W1700	0.91	Or-I	Thailand	369	0.230	1237	0.770	4	2082
W1921	1.04	Or-I	Thailand	458	0.227	1557	0.773	5	1672
W1754	1.93	Or-I	India	620	0.227	2112	0.773	4	956
W1731	1.31	Or-I	India	459	0.227	1566	0.773	9	1658
W0638	1	Or-I	Burma	366	0.224	1268	0.776	4	2054
W1681	0.85	Or-I	India	341	0.224	1182	0.776	2	2167
W1792	2.42	Or-I	Thailand	649	0.224	2250	0.776	17	776
W2307	2.33	Or-I	Laos	659	0.224	2285	0.776	4	744
W0124	0.86	Or-I	India	332	0.224	1152	0.776	1	2207
W2268	1.5	Or-I	Thailand	544	0.224	1890	0.776	7	1251
W0101	1.37	Or-I	India	475	0.223	1651	0.777	5	1561
W0102	1.19	Or-I	India	424	0.223	1474	0.777	9	1785
W0627	1.01	Or-I	Burma	375	0.223	1304	0.777	4	2009

W1557	0.62	Or-II	Thailand	277	0.223	965	0.777	8	2442
W1532	0.81	Or-I	India	344	0.222	1208	0.778	2	2138
W3072	4.33	Or-II	Hainan	438	0.221	1542	0.779	1363	349
W1925	0.83	Or-I	Thailand	316	0.221	1117	0.779	4	2255
W1757	3.41	Or-I	India	709	0.220	2516	0.780	6	461
W1852	1.36	Or-I	Thailand	495	0.219	1764	0.781	53	1380
W0631	0.4	Or-I	Burma	184	0.217	664	0.783		2844
W2306	2.74	Or-I	Laos	651	0.213	2410	0.787	19	612
W2305	1.44	Or-I	Laos	498	0.212	1850	0.788	12	1332
W0589	1.03	Or-I	Malaya	376	0.212	1400	0.788	1	1915
W0626	1.17	Or-I	Burma	371	0.212	1382	0.788	1	1938
W1787	1.53	Or-I	Thailand	516	0.211	1933	0.789	8	1235
W0130	0.9	Or-I	India	330	0.209	1246	0.791	2	2114
W1121	0.94	Or-I	India	317	0.209	1201	0.791	4	2170
W0148	1.39	Or-I	India	443	0.209	1679	0.791	4	1566
W1083	1.28	Or-I	India	429	0.207	1646	0.793	6	1611
W1084	1.36	Or-I	India	442	0.206	1703	0.794	6	1541
W1142	0.95	Or-I	India	338	0.206	1304	0.794	5	2045
W1105	1.39	Or-I	India	443	0.205	1716	0.795	7	1526
W0123	0.17	Or-I	India	73	0.204	285	0.796		3334
W0605	0.8	Or-I	Malaya	302	0.203	1183	0.797	1	2206
W1676	1.01	Or-I	India	340	0.202	1343	0.798	6	2003
W1928	0.95	Or-I	Thailand	312	0.201	1241	0.799	34	2105
W1879	1.21	Or-I	Thailand	428	0.198	1735	0.802	28	1501
W1770	1.05	Or-I	India	387	0.198	1571	0.802	1	1733
W1143	0.68	Or-I	India	244	0.197	995	0.803		2453
042_87_34	19.15	-	Dhoni;India	527	0.195	2175	0.805	915	75
W1082	1.33	Or-I	India	417	0.194	1729	0.806		1546
W0173	1.7	Or-I	Thailand	456	0.192	1915	0.808	4	1317
W0152	1.57	Or-I	India	420	0.192	1766	0.808	3	1503
L89_12	16.34	-	Vientiane;Laos	682	0.190	2899	0.810	46	65
W1795	1.93	Or-I	Thailand	506	0.190	2154	0.810	10	1022
W1873	0.67	Or-II	Thailand	287	0.190	1223	0.810	26	2156
W1124	2.11	Or-II	India	467	0.190	1995	0.810	6	1224
W2271	1.42	Or-I	Thailand	445	0.189	1913	0.811	2	1332
W2193	1.35	Or-I	India	410	0.189	1764	0.811	8	1510
W0147	0.14	Or-I	India	62	0.182	279	0.818		3351
W1751	0.62	Or-I	India	239	0.177	1111	0.823		2342
MV89_80	18.25	-	Medinipur;India	613	0.172	2944	0.828	41	94
W1295	0.92	Or-I	Cambodia	280	0.171	1358	0.829	16	2038
W2298	0.79	Or-I	Laos	255	0.169	1250	0.831	2	2185
W2299	1.23	Or-I	Laos	348	0.169	1710	0.831	5	1629
W1788	2.7	Or-I	Thailand	492	0.164	2503	0.836	3	694

W0145	0.22	Or-II	Thailand	84	0.164	428	0.836	1	3179
W2302	2.31	Or-I	Laos	469	0.162	2429	0.838	4	790
W1893	1.28	Or-I	Thailand	330	0.159	1744	0.841	1	1617
IRGC88812	71.5	-	Laos	573	0.157	3078	0.843	26	15
W1679	1.21	Or-I	India	301	0.154	1658	0.846	4	1729
W2301	1.68	Or-I	Laos	390	0.153	2151	0.847	12	1139
W1914	2.67	Or-I	Thailand	424	0.152	2370	0.848	13	885
W3003	0.38	Or-II	Guangdong	127	0.144	752	0.856	24	2789
W1695	1.04	Or-I	Thailand	256	0.144	1519	0.856		1917
W1690	1.24	Or-I	Thailand	274	0.144	1629	0.856	2	1787
W1737	1.02	Or-I	India	248	0.141	1506	0.859	5	1933
W0587	1.15	Or-II	Malaya	268	0.141	1631	0.859	34	1759
W1742	1.44	Or-II	India	316	0.140	1942	0.860	47	1387
W0635	0.49	Or-II	Burma	125	0.140	770	0.860		2797
W2276	1.18	Or-II	Thailand	272	0.135	1739	0.865	9	1672
W0176	1.17	Or-I	Thailand	241	0.122	1732	0.878	1	1718
W2311	1.92	Or-II	Laos	264	0.122	1899	0.878	62	1467
W2278	2.33	Or-I	Thailand	300	0.115	2318	0.885	292	782
W1750	1.77	Or-I	India	281	0.108	2323	0.892	11	1077
W1668	1.19	Or-III	India	181	0.096	1699	0.904	20	1792
W1881	1.09	Or-I	Thailand	163	0.090	1651	0.910	41	1837
W0632	1.04	Or-I	Burma	161	0.090	1632	0.910	3	1896
W1761	1.11	Or-I	India	183	0.089	1878	0.911	14	1617
W1735	1.51	Or-I	India	179	0.081	2030	0.919	15	1468
W0610	0.61	Or-I	Burma	97	0.080	1123	0.920	2	2470
W1107	1.42	Or-I	India	170	0.076	2053	0.924	3	1466
W0103	0.94	Or-I	India	119	0.072	1538	0.928	2	2033
W1666	0.58	Or-I	India	80	0.067	1112	0.933	10	2490
W1970	1.22	Or-I	Indonesia	124	0.066	1759	0.934	69	1740
W2269	0.99	Or-I	Thailand	118	0.065	1703	0.935	11	1860
W0128	2.14	Or-I	India	156	0.062	2379	0.938	200	957
W1696	0.54	Or-I	Thailand	60	0.056	1018	0.944	6	2608
W1794	1.68	Or-I	Thailand	133	0.052	2424	0.948	3	1132
W0590	1.71	Or-I	Malaya	99	0.042	2238	0.958	80	1275
W1677	2.09	Or-I	India	73	0.029	2425	0.971	30	1164
W0178	0.85	Or-I	Thailand	39	0.025	1528	0.975	1	2124
W1112	0.66	Or-I	India	27	0.022	1179	0.978		2486
CA97_053	16.62	-	Cambodia	55	0.021	2556	0.979	1008	73
W0179	0.88	Or-I	Thailand	33	0.021	1576	0.979	1	2082
W1935	0.76	Or-I	Thailand	26	0.018	1382	0.982	5	2279
W1092	1.17	Or-I	India	36	0.018	1981	0.982	2	1673
W1111	1.53	Or-I	India	32	0.014	2196	0.986	7	1457
W1559	3.92	Or-I	Thailand	40	0.013	2983	0.987	159	510

W1675	1.05	Or-I	India	5	0.003	1781	0.997	2	1904
W1090	0.24	Or-I	India	1	0.002	588	0.998	3	3100
W1117	1.32	Or-I	India	3	0.002	1974	0.998	3	1712
W1086	1.33	Or-I	India	3	0.001	2166	0.999	2	1521

1, Clades were classified by Huang et al.,2012

Huang X, Kurata N, Wei X, Wang ZX, Wang A, Zhao Q, Zhao Y, Liu K, Lu H, Li W, et al. 2012. A map of rice genome variation reveals the origin of cultivated rice. Nature **490**(7421): 497-501.

Table S6 SNP genotype of DR-II. We genotyped all the SNPs within DR-II regions and classified them as Cultivar type or Wild type.

Individual	Depth	Clade[1]	Original place	Cultivar_Type	%	Hete	Unmap
W1086	1.35	Or-I	India	10506	0.998	87	8243
W0178	0.84	Or-I	Thailand	8112	0.997	45	10677
W1090	0.23	Or-I	India	2757	0.996	6	16086
CA97_053	17.2	-	Sopoir Tep;Cambodia	15086	0.995	3243	461
W0639	0.58	Or-I	Burma	5988	0.995	26	12818
W1092	1.2	Or-I	India	9889	0.995	101	8822
W0638	1.06	Or-I	Burma	8682	0.995	94	10041
W1675	1.08	Or-I	India	9255	0.995	66	9493
W0179	0.92	Or-I	Thailand	8633	0.995	91	10091
W1080	1.11	Or-I	India	9575	0.994	77	9156
W1111	1.61	Or-I	India	11587	0.994	186	7017
W1112	0.72	Or-I	India	6606	0.992	42	12163
W0234	1.9	Or-II	Thailand	12688	0.991	188	5875
W1117	1.41	Or-I	India	10145	0.991	145	8483
W0606	1.2	Or-II	Malaya	9165	0.991	108	9506
W0103	0.97	Or-I	India	7961	0.988	87	10720
W1295	0.89	Or-I	Cambodia	8356	0.988	75	10330
W0632	1.08	Or-I	Burma	8596	0.987	109	10040
W0637	0.77	Or-III	Burma	6981	0.985	27	11750
W1559	3.9	Or-I	Thailand	14556	0.985	972	3108
W1119	1.44	Or-III	India	9946	0.985	160	8599
W3094	2.83	Or-III	Yangzi	14794	0.984	605	3228
W1751	0.68	Or-I	India	7384	0.984	28	11329
042_87_34	18.82	-	Dhoni;India	14394	0.983	3061	1163
W1107	1.48	Or-I	India	10747	0.983	108	7824
W3091	1.64	Or-II	Yangzi	11934	0.983	279	6440
W3072	4.26	Or-II	Hainan	14808	0.983	1053	2741
W1858	1.16	Or-II	Thailand	10749	0.982	99	7817
W2036	1.19	Or-III	Burma	10645	0.982	101	7920
W3023	1.41	Or-III	Guangdong	10739	0.982	299	7626
DAL_DHAN	18.54	-	Chakaria;Bangladesh	15027	0.981	2686	852
W3025	0.43	Or-III	Guangdong	5255	0.979	69	13425
W0594	1.27	Or-II	Malaya	9647	0.979	185	8822
W3067	3.37	Or-II	Hainan	14095	0.979	864	3599
W1725	5.3	Or-III	Thailand	15515	0.978	742	2260
W1741	1.17	Or-I	India	10994	0.978	103	7520
W1102	0.74	Or-III	India	6574	0.978	97	12041
W3041	2.97	Or-III	Guangxi	13675	0.977	200	4669
W3030	2.3	Or-III	Guangdong	14653	0.977	674	3186

W3006	1.47	Or-III	Guangdong	10404	0.976	145	8059
P46	14.95	-	Hainan,China	16238	0.975	1261	938
W1742	1.45	Or-II	India	11367	0.972	191	6974
W3068	1.54	Or-II	Hainan	10444	0.972	324	7788
W1935	0.82	Or-I	Thailand	8015	0.971	99	10508
W3044	3.33	Or-III	Guangxi	13828	0.970	567	4042
W3035	0.84	Or-III	Guangdong	8964	0.970	119	9503
W1666	0.62	Or-I	India	6061	0.970	62	12551
W1735	1.51	Or-I	India	10490	0.970	98	7944
W1668	1.21	Or-III	India	9606	0.969	109	8840
W3043	2.19	Or-III	Guangxi	12018	0.969	377	6082
W1757	3.32	Or-I	India	15101	0.969	260	3016
W3001	0.61	Or-III	Guangdong	6223	0.968	67	12368
W0596	0.98	Or-II	Malaya	8277	0.968	195	10115
W3021	1.37	Or-III	Guangdong	10450	0.968	350	7715
W1093	4.86	Or-II	India	13666	0.968	1131	3611
W3022	1.48	Or-III	Guangdong	10786	0.968	302	7415
W0176	1.17	Or-I	Thailand	9211	0.967	66	9274
W1756	0.92	Or-I	India	9151	0.967	126	9274
W3010	2.47	Or-III	Guangdong	12725	0.967	630	5069
W0610	0.63	Or-I	Burma	6126	0.967	40	12483
W3007	2.59	Or-III	Guangdong	13156	0.967	557	4692
W3002	2.6	Or-III	Guangdong	13473	0.966	504	4407
W3004	1.77	Or-III	Guangdong	11562	0.966	368	6521
W3013	1.55	Or-III	Guangdong	11173	0.966	264	7027
W1740	1.82	Or-I	India	12933	0.966	146	5322
W3050	3.4	Or-III	Guangxi	13895	0.965	542	3918
W1943	20.8	Or-III	Jiangxi	17305	0.964	536	370
W3033	1.08	Or-III	Guangdong	9635	0.964	292	8571
W3045	1.77	Or-III	Guangdong	10871	0.963	239	7332
W3028	2.35	Or-III	Guangxi	12085	0.963	424	5882
W0145	0.25	Or-II	Thailand	2344	0.962	7	16418
W1732	1.21	Or-III	India	8658	0.962	104	9755
W3074	2.49	Or-III	Hainan	12512	0.962	272	5576
W0587	1.24	Or-II	Malaya	9361	0.961	121	9004
W1750	1.77	Or-I	India	12360	0.961	249	5755
W0121	0.87	Or-I	India	7410	0.961	211	10938
W3024	0.47	Or-III	Guangdong	5043	0.961	83	13529
W3079	1.45	Or-III	Yangzi	11289	0.961	257	6852
W3040	1.48	Or-III	Guangdong	11993	0.960	331	6041
W3082	1.36	Or-III	Yangzi	11406	0.960	265	6715
W3081	0.88	Or-III	Yangzi	8981	0.960	137	9366
W3080	1.53	Or-III	Yangzi	11483	0.960	248	6647

W3015	1.68	Or-III	Guangdong	11324	0.959	311	6746
W3078	0.94	Or-III	Yangzi	9313	0.958	120	9024
W3092	2.44	Or-III	Yangzi	13418	0.958	436	4418
W3026	0.7	Or-III	Guangdong	6423	0.958	117	12038
W3052	3.26	Or-III	Guangxi	13176	0.958	851	4251
W3060	1.56	Or-III	Hainan	11446	0.958	465	6442
W2311	2.1	Or-II	Laos	11199	0.957	218	6936
W3042	1.38	Or-III	Guangxi	9272	0.956	272	8893
W3014	3	Or-III	Guangdong	13426	0.956	379	4441
W3039	0.91	Or-III	Guangdong	8843	0.956	80	9532
W2193	1.37	Or-I	India	10266	0.956	124	7996
W1534	0.56	Or-III	India	5710	0.956	60	12826
W2269	1.02	Or-I	Thailand	8815	0.955	147	9486
W1114	1.05	Or-III	India	7781	0.955	68	10646
W3019	0.64	Or-III	Guangdong	6567	0.955	157	11826
W0574	1.96	Or-I	Malaya	11501	0.955	147	6667
W3005	0.95	Or-III	Guangdong	8242	0.954	125	10100
W3049	3.23	Or-III	Guangxi	12484	0.954	857	4912
W1737	0.94	Or-I	India	7565	0.953	69	10858
W3105	8.26	Or-I	India	16803	0.953	264	973
W3095	1.48	Or-III	Yangzi	11107	0.953	186	7020
W3027	3.32	Or-III	Guangxi	13413	0.953	945	3835
W3000	0.88	Or-III	Guangdong	8100	0.952	173	10183
W1983	1.76	Or-I	India	11585	0.952	267	6422
W3046	4.08	Or-III	Guangdong	13470	0.952	963	3745
W3077	0.6	Or-III	Yangzi	6638	0.951	52	11832
W1788	2.58	Or-I	Thailand	13170	0.951	205	4813
W1542	0.94	Or-III	Malaya	7442	0.951	80	10957
W3038	1.12	Or-III	Guangdong	10475	0.951	162	7683
W1715	4.5	Or-II	China	13630	0.950	1187	3332
W3075	1.42	Or-III	Yangzi	11753	0.950	360	6134
W3076	0.41	Or-III	Yangzi	4939	0.950	54	13608
W3037	0.89	Or-III	Guangdong	8675	0.949	165	9554
W1928	1.07	Or-I	Thailand	8625	0.949	268	9503
W3011	1.18	Or-III	Guangdong	8808	0.949	284	9293
W1105	1.38	Or-I	India	9606	0.948	115	8618
W1560	1.25	Or-III	Thailand	9249	0.948	137	8968
W0141	0.91	Or-III	India	6596	0.948	87	11816
W2007	1.3	Or-III	India	9947	0.948	154	8213
W1852	1.35	Or-I	Thailand	10982	0.948	264	7010
W0631	0.4	Or-I	Burma	3893	0.947	12	14740
W1124	2.07	Or-II	India	11457	0.947	157	6602
W2008	1.39	Or-III	India	10050	0.947	152	8093

W3093	0.24	Or-III	Yangzi	2896	0.946	4	15797
W1087	5.94	Or-II	India	13661	0.946	1172	3248
W0130	0.75	Or-I	India	6098	0.945	50	12361
W3020	1.41	Or-III	Guangdong	9626	0.945	493	8180
W3034	1.7	Or-III	Guangdong	12312	0.945	638	5191
W0589	0.98	Or-I	Malaya	8024	0.945	88	10279
W3096	0.82	Or-III	Yangzi	7612	0.945	64	10739
W2198	3.74	Or-III	China	14377	0.945	574	3067
W1669	3.12	Or-III	India	14069	0.944	718	3247
W0157	0.96	Or-II	India	7285	0.944	89	11057
W0175	3.38	Or-II	Thailand	12990	0.944	390	4714
W1121	0.96	Or-I	India	7342	0.944	86	10998
W3047	3.23	Or-III	Guangxi	12566	0.944	1267	4283
W1292	0.83	Or-II	Indonesia	7030	0.943	132	11277
W1762	0.7	Or-I	India	6630	0.943	57	11775
W3009	1.84	Or-III	Guangdong	10820	0.943	367	7018
W0590	1.68	Or-I	Malaya	10472	0.943	198	7554
W0151	1.56	Or-I	India	9891	0.942	128	8228
W3016	0.53	Or-III	Guangdong	6035	0.941	78	12371
W1143	0.65	Or-I	India	5685	0.941	29	12789
W1748	5.42	Or-III	India	13159	0.941	1839	3033
W3051	2.4	Or-III	Guangxi	11380	0.940	723	6029
W0128	2.01	Or-I	India	10630	0.939	639	6906
W1753	1.24	Or-I	India	10131	0.939	166	7907
W1084	1.28	Or-I	India	9338	0.939	178	8738
W1083	1.18	Or-I	India	8851	0.938	193	9234
W1532	0.81	Or-I	India	7149	0.937	44	11188
W3048	3.66	Or-III	Guangxi	13553	0.937	394	3998
W1677	2.07	Or-I	India	11180	0.937	233	6691
W1795	1.87	Or-I	Thailand	11377	0.936	459	6246
W1082	1.25	Or-I	India	9087	0.936	168	8983
W3018	1.33	Or-III	Guangdong	9261	0.936	277	8686
W3036	0.47	Or-III	Guangdong	5307	0.935	93	13095
W0126	1.36	Or-III	India	9039	0.935	121	9072
W1738	1.63	Or-I	India	10096	0.934	194	7862
W3031	1.13	Or-III	Guangxi	9729	0.933	346	8090
W2278	2.39	Or-I	Thailand	12716	0.933	518	4714
W1927	1.22	Or-II	Thailand	9419	0.933	194	8568
W1687	1.82	Or-II	India	9911	0.933	542	7692
W1553	0.97	Or-II	Thailand	7092	0.932	194	11061
W0621	1.27	Or-III	Burma	8375	0.932	80	9792
W0163	3.18	Or-II	Thailand	12663	0.931	889	4376
W1794	1.72	Or-I	Thailand	10445	0.931	349	7292

W1782	2.82	Or-III	India	12773	0.930	299	4832
MV89_80	17.93	-	Medinipur;India	15363	0.929	773	1542
W1676	0.97	Or-I	India	7318	0.928	86	10893
W3017	1.62	Or-III	Guangdong	10311	0.928	427	7324
W1122	2.81	Or-II	India	12087	0.928	600	5235
W3070	3.65	Or-III	Hainan	12727	0.927	936	4198
W0629	0.34	Or-III	Burma	3156	0.927	4	15452
W2310	1.88	Or-II	Laos	11696	0.927	311	5927
W3032	0.63	Or-III	Guangxi	6737	0.926	87	11501
W0148	1.33	Or-I	India	9049	0.925	86	8993
W3069	2.35	Or-III	Hainan	11168	0.925	618	6163
W0625	0.45	Or-III	Burma	4050	0.924	20	14460
W3097	1.74	Or-II	Yangzi	10512	0.924	613	6869
W3029	0.58	Or-III	Guangdong	5760	0.924	81	12543
W3098	1.6	Or-II	Yangzi	10090	0.923	536	7398
W0147	0.14	Or-I	India	1753	0.923	4	16958
W1739	1.09	Or-III	India	8733	0.922	184	9208
W1991	1.6	Or-III	India	9415	0.922	165	8484
W2010	0.9	Or-III	India	7621	0.922	79	10512
W1998	1.83	Or-III	India	10590	0.920	147	7206
W0623	0.43	Or-III	Burma	4045	0.920	15	14447
W1989	1.07	Or-III	India	7796	0.919	127	10250
W1784	0.48	Or-III	India	4625	0.919	72	13755
W3008	0.61	Or-III	Guangdong	5854	0.918	85	12401
W3066	2.84	Or-III	Hainan	12421	0.918	334	4998
W1890	2.92	Or-II	Thailand	12509	0.918	686	4550
W2003	1.64	Or-III	India	10198	0.918	154	7599
W1993	1.13	Or-III	India	8402	0.918	154	9555
W2014	0.73	Or-III	India	6354	0.918	63	11876
W1759	2.26	Or-III	India	10569	0.918	1107	6240
W0173	1.61	Or-I	Thailand	9348	0.917	137	8531
W1761	1.03	Or-I	India	8327	0.917	119	9660
W1556	0.97	Or-II	Thailand	7193	0.917	284	10731
W3065	3.38	Or-III	Hainan	11958	0.916	910	4902
W1783	0.36	Or-III	India	3482	0.916	47	15011
W1995	0.74	Or-III	India	6515	0.915	108	11629
W1746	2.02	Or-III	India	10362	0.913	761	6746
W0573	1.47	Or-III	Malaya	8478	0.912	163	9407
W1990	1.25	Or-III	India	8825	0.912	192	8995
W1914	2.53	Or-I	Thailand	11760	0.912	294	5670
W3073	2.74	Or-III	Hainan	11518	0.912	934	5295
W3061	1.01	Or-III	Hainan	8506	0.912	186	9345
W1882	1.83	Or-II	Thailand	10670	0.912	540	6617

W1696	0.5	Or-I	Thailand	4317	0.911	69	14055
W0138	0.76	Or-III	India	5236	0.911	123	12989
W2012	0.88	Or-III	India	7124	0.911	76	10962
W0633	0.38	Or-I	Burma	3478	0.910	9	15030
W1873	0.68	Or-II	Thailand	6308	0.909	135	11790
W0172	0.91	Or-III	Thailand	6421	0.909	88	11710
W1880	2.33	Or-II	Thailand	11426	0.909	955	5336
W3059	1.49	Or-III	Hainan	10096	0.909	369	7380
W0125	1.9	Or-III	India	9666	0.909	240	7982
W0171	5.25	Or-II	Thailand	13015	0.908	1194	3337
W0153	2.08	Or-III	India	9965	0.907	269	7611
W3003	0.35	Or-II	Guangdong	3451	0.907	53	15004
W0134	0.88	Or-III	India	5658	0.907	141	12480
W0136	0.44	Or-III	India	3375	0.906	62	15075
W1096	3.75	Or-II	India	11100	0.906	1484	5122
W1550	3.04	Or-II	Thailand	11387	0.905	1036	5249
W2276	1.11	Or-II	Thailand	9000	0.905	124	8795
W1891	1.39	Or-II	Thailand	9332	0.905	346	8198
W0634	0.78	Or-II	Burma	5821	0.904	146	12278
IRGC103937	10.03	-	Africa	14472	0.904	940	1915
W0164	1.28	Or-II	Thailand	8311	0.904	205	9463
W3056	0.63	Or-III	Hainan	6157	0.903	144	11901
W1747	1.14	Or-I	India	8664	0.903	177	9086
W0593	0.72	Or-III	Malaya	5496	0.902	69	12701
W0628	1.74	Or-II	Burma	9114	0.902	305	8454
W1766	2.58	Or-III	India	11344	0.902	536	5751
W0576	3.02	Or-II	Malaya	10795	0.902	1259	5632
W2282	2.91	Or-II	Thailand	11200	0.901	927	5504
W2306	2.64	Or-I	Laos	12509	0.901	226	4746
W1862	2.04	Or-II	Thailand	10473	0.900	974	6250
W0133	0.59	Or-III	India	4535	0.900	108	13713
W1940	4.36	Or-II	Thailand	12291	0.899	1414	3780
W0549	1.11	Or-III	India	7310	0.899	160	10572
W1881	1.04	Or-I	Thailand	7822	0.899	72	10086
W0132	1	Or-II	India	6199	0.899	178	11785
W1916	3.03	Or-II	Thailand	12164	0.897	1056	4251
W0137	0.68	Or-III	India	4988	0.897	106	13197
W1857	2.93	Or-II	Thailand	11106	0.897	861	5624
W1790	5.28	Or-II	Thailand	13623	0.897	626	3053
W2265	2.36	Or-I	Laos	12124	0.897	233	5115
W2303	1.21	Or-I	Laos	8801	0.896	110	8930
W1142	0.85	Or-I	India	6108	0.896	63	11981
W1749	1.13	Or-I	India	8259	0.896	99	9544

W0624	0.61	Or-II	Burma	4988	0.896	85	13207
W2030	1.99	Or-II	Indonesia	10302	0.895	405	6943
W3012	1.61	Or-III	Guangdong	9982	0.894	290	7409
W2298	0.71	Or-I	Laos	6112	0.894	42	11984
W0168	1.23	Or-I	Thailand	7980	0.894	38	9899
W3054	1.73	Or-III	Hainan	9937	0.894	620	7128
W0135	0.63	Or-III	India	4664	0.894	99	13546
W1832	1.24	Or-I	Thailand	9098	0.894	101	8582
W3064	0.76	Or-III	Hainan	6357	0.894	191	11558
W1974	1.83	Or-II	Indonesia	10112	0.894	435	7112
W1970	1.26	Or-I	Indonesia	8049	0.894	172	9683
W1533	0.75	Or-I	India	6282	0.893	59	11771
IRGC103472	10.29	-	Africa	14340	0.893	868	1943
W2301	1.54	Or-I	Laos	9797	0.893	161	7730
W1780	1.88	Or-II	India	9799	0.892	863	7013
W2284	0.93	Or-II	Thailand	6913	0.892	230	10881
W2022	3.5	Or-II	Indonesia	11513	0.892	817	5136
W0123	0.18	Or-I	India	2071	0.892	8	16531
W2304	2.21	Or-I	Laos	11679	0.892	228	5537
W3062	1.91	Or-III	Hainan	11255	0.891	434	5802
W1685	1.09	Or-I	India	7033	0.891	76	10893
W1879	1.17	Or-I	Thailand	8723	0.891	233	8834
W2299	1.21	Or-I	Laos	8285	0.890	92	9464
W3058	1.21	Or-III	Hainan	8916	0.890	318	8529
W2296	1.24	Or-I	Cambodia	8612	0.890	176	9012
W2307	2.2	Or-I	Laos	11480	0.890	261	5705
W1770	0.97	Or-I	India	7576	0.890	100	10245
W2271	1.29	Or-I	Thailand	8898	0.889	79	8777
W3071	2.6	Or-III	Hainan	10334	0.889	789	6450
W2283	1.9	Or-II	Thailand	9668	0.889	580	7404
W2024	3.61	Or-II	Indonesia	11792	0.889	1117	4477
W3063	1.16	Or-III	Hainan	9068	0.888	236	8419
W1896	2.69	Or-II	Thailand	11883	0.888	555	4929
W1558	2.24	Or-II	Thailand	10113	0.888	571	6903
IRGC103632	10.13	-	Africa	13885	0.887	793	2420
W0149	1.32	Or-III	India	7997	0.887	165	9683
W3053	0.99	Or-III	Hainan	7687	0.887	338	9858
W0170	1.47	Or-I	Thailand	8027	0.887	68	9744
W0635	0.44	Or-II	Burma	4043	0.887	25	14277
W1736	1.39	Or-II	India	8037	0.887	161	9636
W0166	0.55	Or-II	Thailand	3959	0.886	49	14346
W1977	3.22	Or-II	Indonesia	11290	0.886	1253	4870
IRGC104574	7.35	-	Africa	13549	0.886	767	2800

TOG7102	6.25	-	Africa	12125	0.886	271	4902
W1912	1.4	Or-I	Thailand	9187	0.886	157	8332
W2288	2.55	Or-II	Cambodia	10926	0.886	821	5702
IRGC88812	78.63	-	Laos	15359	0.885	629	884
W1126	2.29	Or-II	India	10281	0.885	662	6585
W1893	1.25	Or-I	Thailand	8092	0.885	97	9622
W2305	1.36	Or-I	Laos	9111	0.885	149	8418
W1787	1.36	Or-I	Thailand	8867	0.885	196	8645
W1853	1.11	Or-I	Thailand	8732	0.885	100	8893
W2021	2.15	Or-II	Indonesia	10019	0.885	726	6810
W0106	1.53	Or-I	India	8643	0.885	109	8982
W0630	0.67	Or-I	Burma	5045	0.884	34	13123
W0165	0.75	Or-II	Thailand	5198	0.884	74	12908
W1557	0.55	Or-II	Thailand	4703	0.884	68	13473
W2302	2.22	Or-I	Laos	11089	0.883	344	5964
Yuan3_9	12.46	-	Yunnan	14304	0.883	938	1724
IRGC103469	12.12	-	Africa	13215	0.883	373	3521
W1859	4.02	Or-II	Thailand	11336	0.883	1395	4623
W0605	0.71	Or-I	Malaya	5494	0.883	41	12595
TOG5949	7.75	-	Africa	13030	0.882	344	3745
IRGC104206	9.72	-	Africa	13806	0.882	610	2598
W3055	0.92	Or-III	Hainan	7684	0.882	338	9811
TOG5923	6.05	-	Africa	12739	0.882	328	4089
W1754	1.78	Or-I	India	10503	0.882	185	6765
W0102	1.08	Or-I	India	6721	0.882	69	11170
W1895	1.63	Or-II	Thailand	9535	0.882	593	7453
VOC4	18.27	-	Nepal	12534	0.882	2644	2000
W2005	1.6	Or-III	India	9048	0.882	121	8477
W1619	0.81	Or-I	Thailand	6148	0.882	51	11836
TOG5457	9.63	-	Africa	13196	0.881	471	3420
W1866	1.29	Or-I	Thailand	9402	0.881	121	8072
W1695	0.95	Or-I	Thailand	6503	0.881	176	11305
TOG5467	14.23	-	Africa	13480	0.881	518	3039
W1792	2.26	Or-I	Thailand	10829	0.881	246	6320
W1743	0.96	Or-I	India	7540	0.881	105	10194
W0124	0.83	Or-I	India	5856	0.881	43	12168
IRGC104955	6.67	-	Africa	13607	0.880	600	2805
W2263	1.48	Or-I	Cambodia	8364	0.880	72	9288
W0107	1.62	Or-I	India	8747	0.880	80	8842
W2197	2.7	Or-II	Indonesia	11058	0.880	828	5465
W1981	1.2	Or-II	Indonesia	8067	0.880	372	9320
W1925	0.92	Or-I	Thailand	6597	0.880	104	11258
W3057	1.25	Or-III	Hainan	9005	0.880	421	8203

W1690	1.19	Or-I	Thailand	7434	0.879	116	10290
W0180	2.49	Or-II	Thailand	10287	0.879	292	6869
W0152	1.44	Or-I	India	7922	0.879	100	9750
W0626	1.08	Or-I	Burma	6836	0.879	89	10996
W0101	1.24	Or-I	India	7531	0.879	52	10242
W1921	0.96	Or-I	Thailand	7377	0.879	92	10377
TOG7025	11.7	-	Africa	13111	0.879	377	3566
W2275	0.75	Or-I	Thailand	6241	0.879	48	11711
W0120	0.73	Or-II	India	5706	0.879	168	12199
W1865	1.04	Or-I	Thailand	8364	0.877	121	9207
W0627	0.9	Or-I	Burma	6122	0.877	44	11839
W1546	0.87	Or-I	Thailand	6191	0.877	51	11753
W1681	0.78	Or-I	India	5468	0.877	72	12555
W1679	1.17	Or-I	India	7042	0.877	83	10746
W1854	3.99	Or-II	Thailand	12226	0.877	444	4469
IRGC103520	14.03	-	Africa	13955	0.876	605	2327
W0174	2.5	Or-II	Thailand	10142	0.876	739	6545
W2268	1.42	Or-I	Thailand	9424	0.876	151	7952
W1727	1.15	Or-I	Thailand	7097	0.876	109	10649
W1798	2.78	Or-II	Thailand	11365	0.875	578	5288
W2277	1.08	Or-I	Thailand	7908	0.874	166	9648
W1975	2.23	Or-II	Indonesia	10384	0.874	346	6634
W1547	1.36	Or-I	Thailand	8059	0.874	86	9552
W1726	1.07	Or-I	Thailand	6792	0.873	86	10998
W2266	1.18	Or-II	Laos	7462	0.873	112	10202
W1731	1.23	Or-I	India	7128	0.873	112	10582
W1700	0.86	Or-I	Thailand	5709	0.872	74	12243
W1551	1.23	Or-I	Thailand	7717	0.871	111	9890
W1849	8.42	Or-II	Thailand	13546	0.870	831	2456
W1552	1.19	Or-II	Thailand	7646	0.869	164	9896
IRGC67563	112.31	-	Africa	14369	0.869	732	1587
IRGC68939	118.26	-	Africa	14385	0.868	739	1551
W2025	0.88	Or-II	Indonesia	7074	0.868	158	10553
W0169	0.87	Or-II	Thailand	5623	0.868	56	12324
IRGC101049	114.35	-	Africa	14111	0.867	706	1886
W2272	1.42	Or-II	Thailand	9217	0.867	160	8069
IRGC75500	112.8	-	Africa	14323	0.866	733	1596
IRGC68976	113.32	-	Africa	14152	0.866	703	1821
W1698	1.36	Or-I	Thailand	7692	0.866	135	9845
W1973	2.09	Or-III	Indonesia	7761	0.865	177	9712
IRGC96841	117.5	-	Africa	14102	0.865	713	1844
HK47	14.44	-	Madhya Pradesh;India	13643	0.862	804	2239
L89_12	15.57	-	Vientiane;Laos	13699	0.862	834	2140

W1884	1.9	Or-II	Thailand	9927	0.862	885	6462
W0600	0.84	Or-II	Malaya	5879	0.862	221	11821
W1850	3.93	Or-II	Thailand	11568	0.861	505	4914
W1919	1.49	Or-II	Thailand	9220	0.860	429	7709
W1554	1.92	Or-II	Thailand	9230	0.856	171	7912
W1777	4.1	Or-III	India	11889	0.851	618	4269
W1971	1.5	Or-II	Indonesia	8348	0.850	503	8538
PADI_PADIAN	15.75	-	Kromat Watu;Indonesia	11952	0.844	1925	2776
W2267	1.72	Or-III	Laos	9157	0.842	237	7755
W1979	1.79	Or-II	Indonesia	8739	0.842	384	8103
W1870	3.48	Or-II	Thailand	11213	0.840	964	4555
W1978	1.57	Or-II	Indonesia	8053	0.835	521	8696
W2017	1.8	Or-II	Indonesia	8654	0.835	417	8079
W2308	2.11	Or-II	Laos	9256	0.835	327	7445
W1976	1.49	Or-II	Indonesia	8328	0.830	242	8586
W1683	5.09	Or-II	India	11018	0.825	900	4613
W1972	2.29	Or-II	Indonesia	9285	0.825	495	7115
W1939	1.36	Or-II	Thailand	8253	0.825	218	8636
W0108	1.47	Or-II	India	7470	0.822	278	9491
W1555	0.7	Or-II	Thailand	4959	0.818	63	12739

Table S7 List of 163 genes in the 28 DR-I regions.

#Region	Gene	Ka	Ks	Ka/Ks	Possible Function
chr01:35499730-35510003	LOC_Os01g61380	0.002	0.007	0.337	seed
chr01:35499730-35510003	LOC_Os01g61390	0.001	0.002	0.707	
chr01:35499730-35510003	LOC_Os01g61400	0.001	0	-	
chr01:35499730-35510003	LOC_Os01g61410	0	0	-	
chr01:35839043-35890683	LOC_Os01g61920	0	0.012	0	
chr01:35839043-35890683	LOC_Os01g61930	0.001	0.002	0.629	
chr01:35839043-35890683	LOC_Os01g61940	0.001	0	-	
chr01:35839043-35890683	LOC_Os01g61950	0.010	0.013	0.777	
chr01:35839043-35890683	LOC_Os01g61960	0	0.005	0	
chr01:35839043-35890683	LOC_Os01g61970	0.001	0	-	
chr01:35839043-35890683	LOC_Os01g61980	0.001	0	-	
chr01:35839043-35890683	LOC_Os01g61990	0.002	0	-	
chr01:35839043-35890683	LOC_Os01g62000	0	0.004	0	
chr02:13139975-13160003	LOC_Os02g22084	0	0	-	
chr02:13139975-13160003	LOC_Os02g22090	0.001	0.001	0.614	
chr02:13139975-13160003	LOC_Os02g22100	0.003	0.007	0.465	
chr02:13349983-13360044	LOC_Os02g22370	0.001	0.002	0.616	
chr02:14944936-14962544	LOC_Os02g25580	0.001	0.005	0.144	
chr02:14944936-14962544	LOC_Os02g25590	0	0	-	
chr02:27708263-27721727	LOC_Os02g45540	0.003	0.004	0.638	
chr02:27708263-27721727	LOC_Os02g45550	0	0	-	
chr03:1579192-1602557	LOC_Os03g03600	0	0.013	0	
chr03:1579192-1602557	LOC_Os03g03610	0.001	0	-	
chr03:2483329-2542460	LOC_Os03g05100	0.002	0.017	0.093	
chr03:2483329-2542460	LOC_Os03g05110	0.001	0.010	0.072	
chr03:2483329-2542460	LOC_Os03g05120	0.010	0.010	1.040	
chr03:2483329-2542460	LOC_Os03g05130	0	0	-	
chr03:2483329-2542460	LOC_Os03g05140	0.002	0.001	1.854	anther;shoot
chr03:2483329-2542460	LOC_Os03g05150	0	0	-	
chr03:2483329-2542460	LOC_Os03g05160	0	0	-	
chr03:2483329-2542460	LOC_Os03g05170	0.004	0	-	
chr03:2483329-2542460	LOC_Os03g05180	0.002	0	-	
chr03:2483329-2542460	LOC_Os03g05200	0.003	0.003	0.953	
chr03:2483329-2542460	LOC_Os03g05210	0.001	0.003	0.390	
chr03:2483329-2542460	LOC_Os03g05220	0	0	-	
chr03:2483329-2542460	LOC_Os03g05225	0	0	-	
chr03:2483329-2542460	LOC_Os03g05240	0.002	0.011	0.206	
chr03:2483329-2542460	LOC_Os03g05250	0.004	0.007	0.611	
chr03:2706518-2724355	LOC_Os03g05470	0	0.006	0	
chr03:2706518-2724355	LOC_Os03g05480	0.004	0.002	1.815	

chr03:2706518-2724355	LOC_Os03g05490	0	0.006	0	
chr03:2706518-2724355	LOC_Os03g05500	0	0	-	
chr03:2832279-2854311	LOC_Os03g05680	0.001	0	-	
chr03:2832279-2854311	LOC_Os03g05690	0.003	0	-	
chr03:2832279-2854311	LOC_Os03g05700	0	0	-	
chr03:2832279-2854311	LOC_Os03g05710	0	0	-	
chr03:2832279-2854311	LOC_Os03g05720	0	0.005	0.101	
chr03:2832279-2854311	LOC_Os03g05730	0.001	0	-	
chr03:2896026-2923841	LOC_Os03g05800	0.004	0	-	
chr03:2896026-2923841	LOC_Os03g05806	0.001	0	-	
chr03:2896026-2923841	LOC_Os03g05812	0.001	0.002	0.614	
chr03:2896026-2923841	LOC_Os03g05820	0.001	0.011	0.084	seed
chr03:2896026-2923841	LOC_Os03g05830	0.003	0	-	
chr03:2997394-3195565	LOC_Os03g06000	0	0.011	0	
chr03:2997394-3195565	LOC_Os03g06010	0	0	-	coleoptile
chr03:2997394-3195565	LOC_Os03g06020	0.002	0	-	coleoptile
chr03:2997394-3195565	LOC_Os03g06030	0.005	0.016	0.338	coleoptile
chr03:2997394-3195565	LOC_Os03g06040	0	0.017	0	coleoptile
chr03:2997394-3195565	LOC_Os03g06050	0.002	0	-	coleoptile
chr03:2997394-3195565	LOC_Os03g06060	0.006	0	-	coleoptile
chr03:2997394-3195565	LOC_Os03g06070	0.001	0.005	0.178	
chr03:2997394-3195565	LOC_Os03g06080	0.003	0	-	
chr03:2997394-3195565	LOC_Os03g06090	0.001	0	-	
chr03:2997394-3195565	LOC_Os03g06100	0.002	0	-	
chr03:2997394-3195565	LOC_Os03g06110	0.001	0	-	
chr03:2997394-3195565	LOC_Os03g06120	0	0.003	0	seed
chr03:2997394-3195565	LOC_Os03g06139	0.001	0	-	
chr03:2997394-3195565	LOC_Os03g06160	0.001	0	-	
chr03:2997394-3195565	LOC_Os03g06170	0	0	-	
chr03:2997394-3195565	LOC_Os03g06180	0.001	0.008	0.157	
chr03:2997394-3195565	LOC_Os03g06190	0.001	0	-	
chr03:2997394-3195565	LOC_Os03g06200	0	0.006	0	
chr03:2997394-3195565	LOC_Os03g06210	0.005	0.005	1.060	
chr03:2997394-3195565	LOC_Os03g06220	0	0.003	0	
chr03:2997394-3195565	LOC_Os03g06230	0.004	0.003	1.208	
chr03:2997394-3195565	LOC_Os03g06240	0.004	0.004	0.977	
chr03:2997394-3195565	LOC_Os03g06250	0.002	0	-	
chr03:2997394-3195565	LOC_Os03g06260	0	0	-	
chr03:2997394-3195565	LOC_Os03g06270	0	0	-	
chr03:2997394-3195565	LOC_Os03g06280	0.005	0	-	
chr03:2997394-3195565	LOC_Os03g06290	0.004	0.003	1.320	
chr03:2997394-3195565	LOC_Os03g06330	0	0	-	
chr03:2997394-3195565	LOC_Os03g06340	0.001	0.003	0.325	

chr03:2997394-3195565	LOC_Os03g06350	0	0	-	
chr03:2997394-3195565	LOC_Os03g06360	0.002	0	-	seed
chr03:2997394-3195565	LOC_Os03g06370	0.003	0	-	
chr03:2997394-3195565	LOC_Os03g06379	0.005	0	-	
chr03:2997394-3195565	LOC_Os03g06390	0	0	-	
chr03:3479756-3498823	LOC_Os03g06880	0.001	0.006	0.168	
chr03:3479756-3498823	LOC_Os03g06890	0.001	0.006	0.193	seed
chr03:3479756-3498823	LOC_Os03g06900	0	0	-	
chr03:24189350-24230136	LOC_Os03g43390	0.001	0.007	0.221	
chr03:24189350-24230136	LOC_Os03g43400	0.002	0.005	0.376	root
chr03:24189350-24230136	LOC_Os03g43410	0	0.006	0	root
chr03:24189350-24230136	LOC_Os03g43420	0.001	0.004	0.349	
chr03:24189350-24230136	LOC_Os03g43430	0	0	-	
chr03:24189350-24230136	LOC_Os03g43440	0.001	0	-	
chr03:28469725-28489493	LOC_Os03g49940	0.001	0.006	0.175	
chr03:28469725-28489493	LOC_Os03g49960	0.003	0	-	
chr04:34409908-34420007	LOC_Os04g57780	0.002	0	-	
chr04:34409908-34420007	LOC_Os04g57790	0.002	0	-	
chr04:34409908-34420007	LOC_Os04g57800	0.001	0.002	0.384	
chr04:34469603-34500654	LOC_Os04g57880	0.002	0.003	0.694	
chr04:34469603-34500654	LOC_Os04g57890	0.001	0	-	
chr04:34469603-34500654	LOC_Os04g57900	0.001	0	-	
chr05:21659599-21670204	LOC_Os05g37070	0	0	-	
chr05:21659599-21670204	LOC_Os05g37080	0.003	0	-	
chr05:22789981-22800012	LOC_Os05g38850	0.001	0.006	0.101	
chr05:22869880-22880236	LOC_Os05g38984	0	0.027	0	
chr05:22869880-22880236	LOC_Os05g38990	0.003	0	-	flower
chr05:22869880-22880236	LOC_Os05g39000	0.001	0.007	0.078	
chr05:24026607-24070766	LOC_Os05g40990	0.003	0	-	
chr05:24026607-24070766	LOC_Os05g41000	0	0.003	0	
chr05:24026607-24070766	LOC_Os05g41010	0.001	0	-	
chr05:24026607-24070766	LOC_Os05g41030	0.002	0	-	seed storage
chr05:24026607-24070766	LOC_Os05g41040	0	0	-	
chr05:24026607-24070766	LOC_Os05g41050	0	0.008	0	
chr05:24026607-24070766	LOC_Os05g41060	0.002	0	-	
chr05:24026607-24070766	LOC_Os05g41070	0.005	0.037	0.142	
chr05:24026607-24070766	LOC_Os05g41080	0.006	0	-	
chr05:24300403-24330214	LOC_Os05g41510	0	0.001	0.278	
chr05:24300403-24330214	LOC_Os05g41520	0	0.004	0	
chr05:24300403-24330214	LOC_Os05g41530	0	0	-	
chr05:24300403-24330214	LOC_Os05g41540	0	0.006	0	
chr05:24300403-24330214	LOC_Os05g41550	0.008	0.016	0.503	
chr05:26824033-26840483	LOC_Os05g46250	0.004	0	-	

chr05:26824033-26840483	LOC_Os05g46260	0.001	0.002	0.642	
chr05:26824033-26840483	LOC_Os05g46270	0	0.004	0	
chr05:26824033-26840483	LOC_Os05g46280	0	0.014	0	
chr05:26824033-26840483	LOC_Os05g46290	0.001	0.003	0.297	
chr07:4149981-4163826	LOC_Os07g08170	0.002	0	-	pollen
chr07:4149981-4163826	LOC_Os07g08180	0.002	0.004	0.645	
chr07:25598750-25620217	LOC_Os07g42750	0.002	0.004	0.586	
chr07:25598750-25620217	LOC_Os07g42760	0.003	0.003	0.993	
chr07:25598750-25620217	LOC_Os07g42770	0.002	0.003	0.572	
chr09:22759164-22771433	LOC_Os09g39660	0.002	0.005	0.296	
chr09:22759164-22771433	LOC_Os09g39670	0.001	0.004	0.325	
chr09:22759164-22771433	LOC_Os09g39680	0	0.005	0	
chr09:22910542-22930021	LOC_Os09g39960	0.002	0	-	
chr09:22910542-22930021	LOC_Os09g39970	0.001	0.004	0.308	
chr09:22910542-22930021	LOC_Os09g39980	0.002	0	-	
chr10:21289972-21400480	LOC_Os10g39780	0	0.007	0	
chr10:21289972-21400480	LOC_Os10g39790	0.003	0	-	
chr10:21289972-21400480	LOC_Os10g39800	0.003	0.009	0.307	
chr10:21289972-21400480	LOC_Os10g39810	0	0	-	plant cuticular wax
chr10:21289972-21400480	LOC_Os10g39820	0.002	0	-	light
chr10:21289972-21400480	LOC_Os10g39830	0	0.006	0	
chr10:21289972-21400480	LOC_Os10g39840	0.001	0	-	
chr10:21289972-21400480	LOC_Os10g39850	0.002	0.002	1.001	
chr10:21289972-21400480	LOC_Os10g39860	0.004	0.019	0.224	
chr10:21289972-21400480	LOC_Os10g39870	0	0	-	
chr10:21289972-21400480	LOC_Os10g39880	0.012	0.006	1.931	light
chr10:21289972-21400480	LOC_Os10g39890	0	0.032	0	
chr10:21289972-21400480	LOC_Os10g39900	0.001	0.004	0.203	
chr10:21289972-21400480	LOC_Os10g39910	0.001	0.005	0.152	
chr10:21289972-21400480	LOC_Os10g39920	0.008	0	-	
chr10:21289972-21400480	LOC_Os10g39930	0.002	0.002	0.670	
chr10:21289972-21400480	LOC_Os10g39932	0	0	-	
chr10:21289972-21400480	LOC_Os10g39934	0.003	0	-	
chr10:21289972-21400480	LOC_Os10g39936	0.004	0.005	0.736	
chr10:21289972-21400480	LOC_Os10g39940	0.011	0	-	
chr10:21289972-21400480	LOC_Os10g39950	0.002	0.003	0.945	

Table S8 List of 110 genes in the 28 DR-II regions.

Gene_ID	Possible Function	Gene_ID	Possible Function
LOC_Os01g15438		LOC_Os07g05740	
LOC_Os01g15448		LOC_Os07g05750	
LOC_Os01g15460	rice resistance	LOC_Os07g05820	
LOC_Os03g46320		LOC_Os07g05830	
LOC_Os03g46325		LOC_Os07g05840	
LOC_Os03g46330	root	LOC_Os07g05950	
LOC_Os03g46340		LOC_Os07g05960	
LOC_Os04g43840	*LABA1*, awns	LOC_Os07g05970	
LOC_Os04g43850		LOC_Os07g05984	
LOC_Os04g43860		LOC_Os07g06000	
LOC_Os04g43870		LOC_Os07g06010	
LOC_Os04g43880		LOC_Os07g06130	
LOC_Os04g43890		LOC_Os07g06274	
LOC_Os04g43900		LOC_Os07g06380	
LOC_Os04g43910		LOC_Os07g06390	
LOC_Os04g43916		LOC_Os07g06400	
LOC_Os04g43922		LOC_Os07g07410	
LOC_Os04g44030		LOC_Os07g07420	light
LOC_Os04g44050		LOC_Os07g07430	
LOC_Os04g44060		LOC_Os07g07540	dwarf
LOC_Os04g44290		LOC_Os07g07550	
LOC_Os04g44300		LOC_Os07g07560	cell growth and shape formation
LOC_Os04g44320		LOC_Os07g07570	
LOC_Os04g44330		LOC_Os07g07974	
LOC_Os04g44340		LOC_Os07g07990	
LOC_Os04g44530		LOC_Os07g08030	
LOC_Os04g44540		LOC_Os07g08040	
LOC_Os04g45320		LOC_Os07g08050	
LOC_Os04g45330	flower	LOC_Os07g08060	
LOC_Os04g45340		LOC_Os07g08070	
LOC_Os04g45550		LOC_Os08g37490	response to environmental stress, pathogens and light conditions
LOC_Os04g45560		LOC_Os08g37500	
LOC_Os04g45570		LOC_Os08g37520	
LOC_Os04g45580		LOC_Os08g37530	
LOC_Os04g45665		LOC_Os08g37595	
LOC_Os04g45670		LOC_Os08g37600	metal

LOC_Os04g45690		LOC_Os08g37605	
LOC_Os04g45700	root	LOC_Os08g37610	
LOC_Os04g45710		LOC_Os08g37630	
LOC_Os04g45720		LOC_Os08g37640	
LOC_Os04g45730		LOC_Os08g37650	
LOC_Os04g54670		LOC_Os08g37660	
LOC_Os04g54680		LOC_Os08g37670	
LOC_Os04g54710		LOC_Os08g37760	
LOC_Os04g56850	growth	LOC_Os08g37770	
LOC_Os04g56995		LOC_Os08g37780	
LOC_Os04g57010	resistance	LOC_Os08g37790	
LOC_Os04g57020		LOC_Os08g37874	
LOC_Os04g57520		LOC_Os08g37890	*GAD1*, awn, grain number/length
LOC_Os04g57530	*sh4*, seed shattering	LOC_Os08g37904	
LOC_Os04g57540		LOC_Os08g37920	
LOC_Os04g57550		LOC_Os08g37930	
LOC_Os04g57560		LOC_Os08g37940	
LOC_Os05g51790		LOC_Os08g37950	
LOC_Os07g05720		LOC_Os12g40279	
